# Virtual reality-based versus standard cognitive behavioral therapy for paranoia in schizophrenia spectrum disorders: a randomized controlled trial

**DOI:** 10.1038/s41591-025-03880-8

**Published:** 2025-08-13

**Authors:** Ulrik N. Jeppesen, Ditte L. Vernal, Anne Sofie Due, Lise S. Mariegaard, Amy E. Pinkham, Stephen F. Austin, Maarten Vos, Mads J. Christensen, Nina K. Hansen, Lisa C. Smith, Carsten Hjorthøj, Wim Veling, Merete Nordentoft, Louise B. Glenthøj

**Affiliations:** 1 VIRTU Research Group, Mental Health Center Copenhagen, Copenhagen University Hospital – Mental Health Services CPH, Copenhagen, Denmark; 2 Department of Psychology, University of Copenhagen, Copenhagen, Denmark; 3 Psychiatry, Aalborg University Hospital, Aalborg, Denmark; 4 Department of Clinical Medicine, The Faculty of Medicine, Aalborg University, Aalborg, Denmark; 5 School of Behavioral and Brain Sciences, University of Texas at Dallas, Richardson, TX USA; 6 Mental Health Services East, Copenhagen University Hospital – Psychiatry Region Zealand, Roskilde, Denmark; 7 University of Groningen, Faculty of Medical Sciences, University Medical Center Groningen, Groningen, the Netherlands; 8 Department of Clinical Medicine, Faculty of Health and Medical Sciences, University of Copenhagen, Copenhagen, Denmark; 9 Section of Epidemiology, Department of Public Health, University of Copenhagen, Copenhagen, Denmark

**Keywords:** Randomized controlled trials, Schizophrenia, Psychology, Psychosis

## Abstract

Paranoia is a distressing and prevalent symptom in schizophrenia spectrum disorders. Virtual reality-based cognitive behavioral therapy for paranoia (VR-CBTp) has been proposed to augment behavioral interventions by providing controlled and safe virtual environments in which social situations inducing paranoid anxiety can be manipulated, allowing for new therapeutical possibilities such as gradual exposure and repetition. This assessor-masked, randomized parallel group superiority trial investigated the efficacy of VR-CBTp compared to standard CBTp. Participants were randomized to receive ten sessions of VR-CBTp or CBTp, both on top of treatment as usual. Intention-to-treat analyses included 254 participants (VR-CBTp: *n* = 126, CBTp: *n* = 128). Outcomes were assessed at baseline, treatment cessation and follow-up (6 months after treatment cessation). The primary outcome was Ideas of Persecution subscale from the Green Paranoid Thoughts Scale, measured at treatment cessation. There was not a statistically significant between-group difference on the primary outcome at endpoint (effect estimate: 2% in favor of VR-CBTp; 95% confidence interval: −11% to +17%; Cohen’s *d* = 0.04; *P* = 0.77, based on exponentiated log-transformed data). No deaths or violent incidents involving law enforcement occurred during the study. In conclusion, VR-CBTp was not superior to CBTp in reducing schizophrenia-spectrum-disorders-related paranoia. ClinicalTrials.gov registration: NCT04902066.

## Abstract (web summary)

A randomized controlled trial found no difference in paranoid ideations between virtual reality-based and gold-standard cognitive behavioral therapy for patients with schizophrenia spectrum disorders, in contrast to previous trials using wait-list or passive controls.

## Main

Schizophrenia spectrum disorders (SSD) (*International Classification of Diseases, Tenth Revision* (ICD-10), F-20-29) have profound impacts, imposing substantial costs on affected individuals, their families and society at large^1^. Globally, SSD is the 18th leading cause of years lived with disability among all diseases, injuries and risk factors^2^. Paranoia is a common and highly distressing symptom in SSD affecting at least 70% of patients^3–5^. Paranoia encompasses ideas of social self-reference and persecution. Ideas of social self-reference refer to exaggerated experiences of feeling observed or receiving unusual attention from others, often accompanied by a sense of being subjected to judgemental looks, gossip or heightened scrutiny. Persecutory ideas add threat beliefs to these experiences, whereby others are perceived as intentionally seeking to cause harm. For instance, the feeling of observation can be perceived as surveillance aimed at theft or attempts on one’s life. Paranoia ranges in severity from paranoid ideation, a milder condition that does not reach delusional intensity and is observed across various disorders, such as SSD and certain personality disorders (as defined in both ICD-10 and *Diagnostic and Statistical Manual of Mental Disorders, Fifth Edition*), to fixed delusions, which are defined by persistent, false beliefs that remain unchanged despite contradictory evidence^6^.

Paranoia contributes to social avoidance and loneliness in individuals with SSD. These factors are closely linked to poorer social functioning, reduced quality of life and adverse long-term health outcomes^7–9^. Moreover, one-third of patients experiencing a first-episode psychosis continue to exhibit psychotic symptoms, including paranoia, 1 year after initiating treatment with antipsychotic medication^10–12^. This underscores the need for effective, adjunctive treatments to further address paranoia.

Over the past three decades, interest in applying cognitive behavioral therapy for paranoia (CBTp) and other psychotic symptoms in SSD has grown notably^13–19^. CBTp differs from CBT for conditions such as depression by addressing elements specific for paranoia. These include the misinterpretation of others’ intentions, psychosis-specific cognitive biases and the patient’s experiential world, which may involve other psychotic symptoms like hearing voices. Further, the therapeutic alliance requires particular care, as mistrust often extends to the therapist. Treatment success is primarily defined by a reduction in belief intensity and associated distress, rather than the complete elimination of paranoia. A previous umbrella review of meta-analyses^20^ concluded that CBTp for delusions and other psychotic symptoms yields small to medium effect sizes compared to treatment as usual (TAU) at treatment cessation; however, these effects were not maintained after 6–12 months.

A symptom-specific approach, targeting a single symptom such as paranoia rather than a broad range of psychotic symptoms, may enhance the efficacy of interventions for psychotic symptoms in SSD. By enabling more precise interventions and outcome measurements, this approach has shown promise. A systematic review of randomized clinical trials found that paranoia-focused interventions yielded effect sizes approaching a moderate level^21^.

While targeting specific symptoms may enhance treatment precision, another important barrier to efficacy is patients’ frequent reliance on avoidance and safety behaviors to cope with paranoid threats^22^. Although exposure and other behavioral components are considered effective in reducing these maladaptive behaviors, implementation can be challenging due to the difficulty of organizing and controlling real-life scenarios^22^.

To overcome these challenges, immersive virtual reality (VR) has been suggested as a promising tool within therapist-guided CBT specifically targeting paranoia (VR-CBTp)^23^. VR employs computer-generated simulations to immerse users in interactive three-dimensional environments, typically via a headset that tracks movement and dynamically adjusts the scene in real time. This modality allows for controllable, repeatable and interactive experiences tailored to individual therapeutic goals. One of the advantages of VR over standard CBT approaches is the ability to precisely design and manipulate social environments to match specific paranoid fears, for example, crowded public spaces, unfamiliar individuals or ambiguous social cues. This level of control allows therapists to adjust the intensity and nature of exposure in a graded manner, ensuring that patients face realistic but manageable scenarios, provided that they experience the sense of presence in the VR environment. Moreover, VR environments reduce external unpredictability, which can make traditional in vivo exposure more difficult for both therapists and patients. The structured and predictable nature of VR scenarios may also facilitate patient engagement, particularly among individuals who might otherwise refuse or avoid exposure-based exercises in real-world settings^23^. Consequently, VR-CBTp can facilitate faster, more consistent delivery of behavioral interventions, allowing for more session time to be spent on therapeutic work^23^.

Preliminary studies have shown promising effects for therapist-guided VR interventions for paranoia when compared to VR exposure alone or wait-list controls^24,25^. Furthermore, automated VR therapies have also been investigated, and while these show feasibility, shorter interventions have not demonstrated superiority over comparators^26,27^. Together, these findings suggest that VR may enhance the behavioral effectiveness of CBTp and offer a scalable pathway for symptom-specific treatment.

Building on these studies, we initiated the FaceYourFears randomized controlled, superiority trial to evaluate the efficacy of symptom-specific, therapist-guided VR-CBTp. The primary hypothesis tested whether VR-CBTp would be more effective than the current gold-standard, symptom-specific CBTp in reducing paranoia. Specifically, the comparison focused on changes in the Ideas of Persecution subscale from the Green Paranoid Thoughts Scale (GPTS) at the end of treatment^6^. Secondary hypotheses posited that VR-CBTp would be more effective than CBTp in reducing ideas of social self-reference, social anxiety and safety behaviors, and in improving emotion recognition and psychosocial functioning in patients with SSD.

## Results

### Patient disposition

**Fig. 1:**
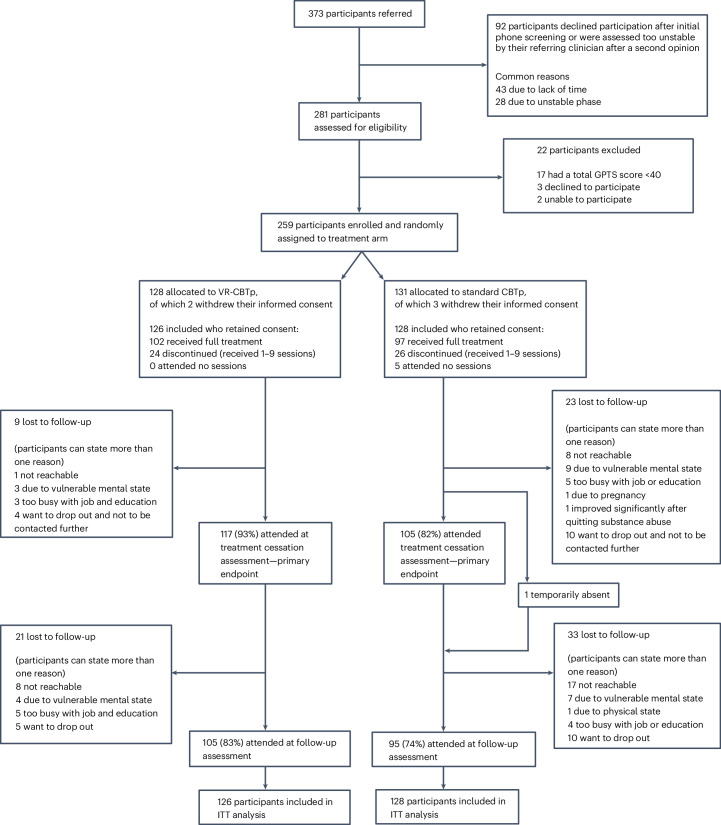
CONSORT diagram of all participants who were assessed for eligibility for the trial, randomized to VR-CBTp + TAU or CBTp + TAU and followed up to 6 months posttreatment cessation. Treatment cessation, mean = 4.5 months (95% confidence interval (CI) 4.3–4.6) after baseline; follow-up, mean = 10.5 months (95% CI 10.3–10.7) after baseline. Source data

Between 26 March 2021 and 30 September 2023, a total of 373 referrals were screened (Fig. 1). Of these, 92 potential participants either declined participation after initial phone screening or were deemed too unstable by their referring clinician following a second opinion. Of the 281 individuals who were assessed for eligibility criteria, 22 were subsequently excluded; 17 did not meet the inclusion criteria of a GPTS total score ≥40 (that is, the sum score of Ideas of Persecution and Ideas of Social Self-reference), three declined to participate during or shortly after baseline assessment and two were deemed unable to participate due to their psychiatric condition, as they were acutely admitted shortly thereafter. Enrollment of the first participant took place on 9 April 2021 and the last enrollment occurred on 15 November 2023. A total of 259 participants completed the informed consent process and were enrolled and randomized. However, five withdrew their consent later in the study (VR-CBTp: *n* = 2, CBTp: *n* = 3), including two who withdrew late in the study period, preventing us from reaching the target sample size of 256. Of the enrolled participants who retained their consent, 126 were randomly assigned to the VR-CBTp group and 128 were randomly assigned to the CBTp group. Altogether 254 patients were included in analyses.

Sociodemographic characteristics were balanced at baseline (Table 1). Prespecified outcomes were balanced except for the following exploratory outcomes, where differences were evaluated as clinically relevant: Intentionality Bias Task (IBT) Automatic^28^, where VR-CBTp scored lower than CBTp; and two items from the Trauma and Life Events checklist (TALE)^29^, items 4 (sudden change in life circumstances) and 8 (physical abuse—familiar perpetrator), where VR-CBTp scores were higher (Table 2).

**Table 1:** Clinical and sociodemographic characteristics of the ITT population across both trial arms at baseline

Variable	VR-CBTp	CBTp	Total
Age, years	27.7 (23.0–35.0) *n* = 126	26.5 (22.7–31.8) *n* = 128	26.8 (22.8–33.1) *n* = 254
Sex			
Female	72 (57.1%)	74 (57.8%)	146 (57.5%)
Male	54 (42.9%)	54 (42.2%)	108 (42.5%)
Education			
Primary (in Denmark up until age 14)	38 (30.2%)	44 (34.4%)	82 (32.3%)
Secondary	43 (34.1%)	51 (39.8%)	94 (37.0%)
Vocational education	17 (13.5%)	12 (9.4%)	29 (11.4%)
University degree	25 (19.9%)	18 (14.1%)	43 (16.9%)
Other^a^	3 (2.4%)	3 (2.3%)	6 (2.4%)
Occupational status			
Employment (full time or part time)	17 (13.5%)	27 (21.1%)	44 (17.3%)
Student	19 (15.1%)	28 (21.9%)	47 (18.5%)
Unemployment	58 (46.0%)	52 (40.6%)	110 (43.3%)
Disability retirement (or retired)	25 (19.8%)	17 (13.3%)	42 (16.5%)
Housewife or husband	1 (0.8%)	0	1 (0.4%)
Internship as unemployed	6 (4.8%)	4 (3.1%)	10 (3.9%)
Diagnosis			
F20 Schizophrenia	94 (74.6%)	90 (70.3%)	184 (72.4%)
F21 Schizotypal disorder	20 (15.9%)	27 (21.1%)	47 (18.5%)
F22 Paranoid psychoses	6 (4.7%)	4 (3.1%)	10 (3.9%)
F25 Schizoaffective psychosis	5 (4.0%)	3 (2.3%)	8 (3.2%)
F29 Nonorganic psychosis	1 (0.8%)	4 (3.1%)	5 (2.0%)
Outpatient routine care setting			
Early intervention services (OPUS)	59 (46.8%)	63 (49.2%)	122 (48.0%)
Flexible Assertive Community teams (F-ACT)	67 (53.2%)	62 (48.4%)	129 (50.8%)
Other	0	3 (2.3%)	3 (1.2%)
Olanzapine equivalent (mg d^−1^)	10.1 (3.4–20) *n* = 126	10.1 (5.0–18.2) *n* = 128	10.1 (0–18.9) *n* = 254
TALE (answering yes)^b^			
Exposure to war (item 1)	1 (0.8%) *n* = 125	4 (3.1%) *n* = 124	5 (2.0%) *n* = 249
Permanent separation from primary caregiver (item 2)	56 (44.8%) *n* = 125	63 (49.2 %) *n* = 128	119 (47.0%) *n* = 253
Period of separation from primary caregiver (item 3)	50 (40.0%) *n* = 125	51 (39.8%) *n* = 128	101 (39.9%) *n* = 253
Sudden change in life circumstances (item 4)	66 (52.8%) *n* = 125	51 (39.8%) *n* = 128	117 (46.2%) *n* = 253
Bullying (item 5)	83 (66.4%) *n* = 125	91 (71.1%) *n* = 128	174 (68.8%) *n* = 253
Discrimination (item 6)	45 (36.0%) *n* = 125	53 (41.4%) *n* = 128	98 (38.7%) *n* = 253
Emotional abuse (item 7)	67 (53.6%) *n* = 125	55 (43.0%) *n* = 128	122 (48.2%) *n* = 253
Physical abuse—familiar perpetrator (item 8)	53 (42.4%) *n* = 125	38 (29.7%) *n* = 128	91 (36.0%) *n* = 253
Witnessing abuse at home (item 9)	43 (34.4%) *n* = 125	52 (40.6%) *n* = 128	95 (37.5%) *n* = 253
Physical abuse—nonfamiliar perpetrator (item 10)	47 (37.6%) *n* = 125	49 (38.6%) *n* = 127	96 (38.1%) *n* = 252
Emotional neglect (item 11)	63 (50.4%) *n* = 125	60 (46.9%) *n* = 128	123 (48.6%) *n* = 253
Physical neglect (item 12)	16 (12.8%) *n* = 125	13 (10.2%) *n* = 128	29 (11.5%) *n* = 253
Sexual abuse before the age of 16 (item 13)	31 (24.8%) *n* = 125	35 (27.3%) *n* = 128	66 (26.1%) *n* = 253
Sexual abuse since the age of 16 (item 14)	45 (36.0%) *n* = 125	44 (34.4%) *n* = 128	89 (35.2%) *n* = 253
‘Unusual’ experiences (the questionnaire exemplifies typical psychotic experiences) (item 15)	107 (87.0%) *n* = 123	106 (82.8%) *n* = 128	213 (84.9%) *n* = 251
‘Unusual’ behavior (the questionnaire illustrates certain bizarre behaviors) (item 16)	39 (31.2%) *n* = 125	35 (27.3%) *n* = 128	74 (29.2%) *n* = 253
Distressing events during interaction with mental health services (item 17)	32 (25.6%) *n* = 125	30 (23.4%) *n* = 128	62 (24.5%) *n* = 253
Criminal justice (item 18)	23 (18.4%) *n* = 125	19 (14.8%) *n* = 128	42 (16.6%) *n* = 253
Noninterpersonal (item 19)	45 (36.0%) *n* = 125	47 (36.7%) *n* = 128	92 (36.4%) *n* = 253
Use of psychoactive substances during the past week (answering yes)	6 (4.8%) *n* = 126	8 (6.5%) *n* = 123	14 (5.6%) *n* = 249
Alcohol intake, units during the past week	0 (0–3) *n* = 126	0 (0–4) *n* = 127	0 (0–4) *n* = 253
Self-harm during the past week (answering yes)	14 (11.1%) *n* = 126	12 (9.4%) *n* = 128	26 (10.2%) *n* = 254
Big-5^c^			
Openness	3.14 (2.99–3.30%) *n* = 126	3.14 (3.01–3.27%) *n* = 127	3.14 (3.04–3.24%) *n* = 253
Conscientiousness	3.29 (3.13–3.44%) *n* = 126	3.29 (3.14–3.44%) *n* = 127	3.29 (3.18–3.40% *n* = 253
Extraversion	2.20 (2.08–2.33%) *n* = 126	2.15 (2.02–2.29%) *n* = 127	2.18 (2.09–2.27%) *n* = 253
Agreeableness	3.60 (3.48–3.72%) *n* = 126	3.51 (3.39–3.63%) *n* = 127	3.55 (3.47–3.64%) *n* = 253
Neuroticism	4.4 (3.8–4.6) *n* = 126	4.4 (3.8–4.8) *n* = 127	4.4 (3.8–4.8) *n* = 253

Values are presented as *n* (*n*%), indicating the frequency and its corresponding percentage of the total; *n* (*n*–*n*%) indicates the mean value and its 95% CI; *n* (*n*–*n*) indicates the median and its 25th and 75th percentiles in non-normal distributed samples; and *n* = indicates the number of observations.

^a^Education: ‘Other’ primarily refers to the lack of completion of primary education.

^b^TALE: During a review of ‘Other trauma’ (item 20), cases have been allocated to other items wherever possible.

^c^Big-5: scored on a 5-point Likert scale.

**Table 2:** Between-group adjusted mean difference and estimated effect size on exploratory outcomes

Variable	VR-CBTp mean (95% CI)	CBTp mean (95% CI)	Adjusted mean difference (standard error)	95% CI for the adjusted mean difference; *P* value	Cohen’s *d*
SAPS Global					
Baseline	7.8 (7.4–8.3%) *n* = 126	7.7 (7.1–8.2%) *n* = 128	–	–	–
Treatment cessation	6.3 (6.0–6.7%)	6.5 (6.1–6.8%)	0.12 (0.26)	(−0.40–0.63%); 0.65	0.09
Follow-up	5.7 (5.2–6.2%)	5.9 (5.3–6.4%)	0.15 (0.37)	(−0.58–0.89%); 0.68	0.07
SAPS Composite					
Baseline	22.9 (20.6–25.0%) *n* = 126	21.8 (19.7–23.9%) *n* = 128	–	–	–
Treatment cessation	16.6 (15.5–17.8%)	17.3 (16.1–18.6%)	0.70 (0.86)	(−1.00–2.40%); 0.42	0.10
Follow-up	14.4 (13.0–15.8%)	15.2 (13.7–16.8%)	0.81 (1.06)	(−1.28–2.90%); 0.45	0.10
BNSS total					
Baseline	23.4 (21.7–25.2%) *n* = 126	23.4 (21.4–25.3%) *n* = 126	–	–	–
Treatment cessation	20.5 (19.3–21.6%)	21.2 (20.1–22.4%)	0.77 (0.82)	(−0.85–2.40%); 0.35	0.11
Follow-up	19.5 (18.0–21.0%)	18.5 (16.7–20.3%)	−1.02 (1.20)	(−3.39–1.35%); 0.40	−0.11
CDSS total					
Baseline	6.1 (5.4–6.8%) *n* = 124	5.7 (5.1–6.3%) *n* = 126	–	–	–
Treatment cessation	4.3 (3.7–4.9%)	5.0 (4.4–5.6%)	0.99^a^ (0.23)	(0.63–1.57%)^a^; 0.97	0^b^
Follow-up	4.7 (4.0–5.4%)	4.8 (4.0–5.8%)	0.13 (0.56)	(−0.97–1.22%); 0.82	0.02
COGDIS total					
Baseline	18.1 (16.3–19.8%) *n* = 126	17.7 (16.0–19.3%) *n* = 127	–	–	–
Treatment cessation	15.7 (14.4–17.1%)	18.3 (16.8–19.9%)	2.58 (1.03)	(0.55–4.62%); 0.013^c^	0.31
Follow-up	15.8 (13.9–17.6%)	16.8 (14.8–18.9%)	1.06 (1.41)	(−1.73–3.85%); 0.45	0.09
Trustworthiness task					
Baseline	−0.22 (−0.33 to −0.13%) *n* = 126	−0.30 (−0.42 to −0.18%) *n* = 128	–	–	–
Treatment cessation	−0.17 (−0.27 to −0.07%)	−0.10 (−0.21–0.01%)	0.07 (0.08)	(−0.08–0.22%); 0.38	0.11
Follow-up	−0.14 (−0.25 to −0.02%)	−0.10 (−0.24–0.03%)	0.03 (0.09)	(−0.14–0.21%); 0.71	0.06
SSPA (SCOPE variables^48^)					
Baseline	4.09 (3.99–4.20%) *n* = 125	4.14 (4.05–4.24%) *n* = 126	–	–	–
Treatment cessation	4.38 (4.31–4.46%)	4.30 (4.20–4.39%)	−0.09 (0.06)	(−0.21−0.04%); 0.17	−0.16
Follow-up	4.42 (4.35–4.49%)	4.39 (4.29–4.49%)	−0.03 (0.06)	(−0.15–0.10%); 0.66	−0.06
IBT total					
Baseline	0.53 (0.51–0.56%) *n* = 124	0.57 (0.55–0.60%) *n* = 127	–	–	–
Treatment cessation	0.52 (0.50–0.54%)	0.54 (0.52–0.56%)	0.02 (0.02)	(−0.01–0.06%); 0.16	0.15
Follow-up	0.52 (0.49–0.54%)	0.54 (0.51–0.56%)	0.02 (0.02)	(−0.02–0.06%); 0.41	0.13
IBT automatic					
Baseline	0.58 (0.54–0.63%) *n* = 124	0.65 (0.61–0.70%) *n* = 127	–	–	–
Treatment cessation	0.56 (0.52–0.60%)	0.61 (0.57–0.65%)	0.05 (0.03)	(−0.01–0.11%); 0.12	0.03
Follow-up	0.56 (0.52–0.61%)	0.58 (0.53–0.63%)	0.01 (0.04)	(−0.06–0.08%); 0.79	0.01
IBT control					
Baseline	0.38 (0.34–0.43%) *n* = 124	0.38 (0.34–0.42%) *n* = 127	–	–	–
Treatment cessation	0.42 (0.37–0.46%)	0.40 (0.35–0.45%)	−0.02 (0.03)	(−0.08–0.05%); 0.61	−0.08
Follow-up	0.39 (0.34–0.43%)	0.38 (0.33–0.43%)	−0.01 (0.03)	(−0.07–0.06%); 0.85	−0.04
SIDAS					
Baseline	5 (1–15)^d^ *n* = 126	7 (0–14)^d^ *n* = 127	–	–	–
Treatment cessation	6.3 (4.9–7.6%)	8.5 (7.1–9.9%)	1.51^a^ (0.37)	(0.73–3.11%)^a^; 0.26	0.14^b^
Follow-up	6.8 (5.2–8.3%)	8.6 (6.8–10.3%)	1.80 (1.20)	(−0.56–4.17%); 0.13	0.19
BCSS—negative self					
Baseline	10.1 (9.2–11.0%) *n* = 126	10.2 (9.4–11.1%) *n* = 128	–	–	–
Treatment cessation	7.8 (7.1–8.4%)	8.6 (7.9–9.4%)	0.87 (0.50)	(−0.12–1.87%); 0.09	0.20
Follow-up	7.1 (6.4–7.9%)	8.1 (7.2–8.9%)	0.94 (0.58)	(−0.21–2.01%); 0.11	0.22
BCSS—negative others					
Baseline	7.5 (6.7–8.4%) *n* = 126	8.7 (7.8–9.6%) *n* = 128	–	–	–
Treatment cessation	5.7 (5.0–6.4%)	6.6 (5.9–7.4%)	1.16^a^ (0.23)	(0.73–1.84%)^a^; 0.52	0.081^b^
Follow-up	6.3 (5.5–7.1%)	6.0 (5.1–6.8%)	−0.33 (0.60)	(−1.52–0.86%); 0.59	−0.06
BCSS—positive self					
Baseline	8.1 (7.1–9.0%) *n* = 126	7.5 (6.7–8.3%) *n* = 128	–	–	–
Treatment cessation	8.8 (0.4%)	8.7 (8.0–9.5%)	–0.05 (0.51)	(−1.05–0.95%); 0.92	−0.02
Follow-up	9.3 (8.4–10.1%)	9.7 (8.8–10.7%)	0.46 (0.65)	(−0.82–1.73%); 0.48	0.08
BCSS—positive others					
Baseline	9.1 (8.4–9.9%) *n* = 126	8.5 (7.8–9.1%) *n* = 128	–	–	–
Treatment cessation	10.2 (9.5–10.9%)	9.8 (9.1–10.6%)	–0.34 (0.52)	(−1.38–0.67%); 0.50	−0.08
Follow-up	10.4 (9.5–11.2%)	10.2 (9.2–11.2%)	−0.17 (0.68)	(−1.51–1.16%); 0.80	−0.04
DACOBS—jumping to conclusion					
Baseline	22.3 (21.3–23.2%) *n* = 126	22.1 (21.2–23.0%) *n* = 128	–	–	–
Treatment cessation	21.9 (21.1–22.7%)	21.5 (20.6–22.3%)	−0.42 (0.60)	(−1.61–0.77%); 0.49	−0.08
Follow-up	22.0 (21.1–22.9%)	21.5 (20.6–22.5%)	−0.48 (0.67)	(−1.80–0.85%); 0.48	−0.09
DACOBS—belief inflexibility					
Baseline	21.0 (20.1–22.0%) *n* = 126	21.2 (20.3–22.2%) *n* = 128	–	–	–
Treatment cessation	20.5 (19.7–21.2%)	19.9 (19.1–20.7%)	−0.55 (0.56)	(−1.66–0.56%); 0.33	−0.13
Follow-up	19.9 (19.1–20.8%)	19.6 (18.7–20.5%)	−0.32 (0.61)	(−1.53–0.89%); 0.60	−0.06
DACOBS—attention for threat					
Baseline	30.9 (30.0–31.8%) *n* = 126	30.3 (29.4–31.3%) *n* = 128	–	–	–
Treatment cessation	27.0 (26.1–27.9%)	27.8 (26.8–28.7%)	0.78 (0.66)	(−0.52–2.07%); 0.24	0.15
Follow-up	26.3 (25.1–27.4%)	26.8 (25.5–28.0%)	0.47 (0.86)	(−1.24–2.17%); 0.59	0.08
DACOBS—external attribution					
Baseline	21.9 (20.8–22.9%) *n* = 126	22.3 (21.3–23.4%) *n* = 128	–	–	–
Treatment cessation	20.2 (19.5–21.4%)	20.6 (19.8–21.4%)	0.40 (0.55)	(−0.70–1.49%); 0.48	0.07
Follow-up	20.0 (19.1–20.9%)	19.4 (18.4–20.3%)	−0.65 (0.65)	(−1.94–0.63%); 0.32	−0.11
DACOBS—social cognitive problems					
Baseline	29.4 (28.4–30.4%) *n* = 126	29.7 (30.4%) *n* = 128	–	–	–
Treatment cessation	26.0 (25.1–26.9%)	26.7 (25.7–27.6%)	0.66 (0.68)	(−0.68–1.99%); 0.34	0.13
Follow-up	25.4 (24.3–26.6%)	26.4 (25.2–27.6%)	0.99 (0.83)	(−0.65–2.64%); 0.24	0.15
DACOBS—subjective cognitive problems					
Baseline	28.4 (27.3–29.6%) *n* = 126	29.4 (28.4–30.4%) *n* = 128	–	–	–
Treatment cessation	26.8 (25.9–27.8%)	27.3 (26.3–28.4%)	0.48 (0.71)	(−0.93–1.89%); 0.50	0.09
Follow-up	26.3 (25.2–27.4%)	27.1 (25.8–28.4%)	0.80 (0.88)	(−0.93–2.53%); 0.36	0.12
DACOBS—safety behavior					
Baseline	23.0 (21.8–24.3%) *n* = 126	22.7 (21.5–24.0%) *n* = 128	–	–	–
Treatment cessation	20.3 (19.2–21.3%)	20.6 (19.5–21.7%)	0.29 (0.76)	(−1.21–1.79%); 0.70	0.05
Follow-up	19.3 (18.1–20.4%)	18.4 (17.2–19.7%)	−0.86 (0.86)	(−2.55–0.84%); 0.32	−0.13
SFS—social engagement/withdrawal					
Baseline	94.9 (93.3–96.5%) *n* = 126	95.5 (93.8–97.2%) *n* = 126	–	–	–
Treatment cessation	97.7 (96.4–99.0%)	96.6 (95.2–98.0%)	−1.07 (0.97)	(−2.98–0.85%); 0.27	−0.14
Follow-up	98.1 (96.3–99.8%)	99.3 (97.5–101.2%)	1.26 (1.31)	(−1.33–3.86%); 0.34	0.12
SFS—interpersonal behavior					
Baseline	109.3 (106.5–112.1%) *n* = 126	111.0 (108.1–113.9%) *n* = 127	–	–	–
Treatment cessation	113.8 (111.5–116.1%)	112.0 (109.6–114.4%)	−1.82 (1.67)	(−5.10–1.47%); 0.28	−0.14
Follow-up	113.7 (111.0–116.4%)	115.2 (112.1–118.3%)	1.55 (2.12)	(−2.64–5.74%); 0.47	0.09
SFS—recreation					
Baseline	107.3 (104.7–110.0%) *n* = 124	104.3 (101.8–106.8%) *n* = 127	–	–	–
Treatment cessation	107.5 (105.8–109.3%)	107.8 (105.8–109.7%)	0.26 (1.35)	(−2.40–2.93%); 0.85	0.02
Follow-up	107.3 (105.0–109.7%)	110.4 (107.7–113.0%)	3.03 (1.79)	(−0.51–6.57%); 0.09	0.21
SFS—independence-competence					
Baseline	99.5 (97.5–101.5%) *n* = 123	97.9 (96.0–99.8%) *n* = 127	–	–	–
Treatment cessation	103.5 (102.0–104.9%)	102.1 (100.5–103.7%)	−1.37 (1.13)	(−3.59–0.86%); 0.23	−0.16
Follow-up	103.9 (101.9–106.0%)	104.2 (102.0–106.4%)	0.33 (1.49)	(−2.62–3.28%); 0.83	0.02
SFS—independence-performance					
Baseline	97.0 (95.0–99.0%) *n* = 126	96.8 (94.8–98.7%) *n* = 127	–	–	–
Treatment cessation	100.1 (98.7–101.5%)	99.6 (98.1–101.1%)	−0.54 (1.04)	(−2.59–1.52%); 0.61	−0.06
Follow-up	101.3 (99.5–103.1%)	101.5 (99.6–103.5%)	0.24 (1.35)	(−2.42–2.92%); 0.85	0.02
SFS—employment-occupation					
Baseline	101.6 (99.1–104.0%) *n* = 120	104.7 (102.4–107.0%) *n* = 126	–	–	–
Treatment cessation	104.0 (102.3–105.7%)	104.4 (102.6–106.3%)	0.45 (1.28)	(−2.07–2.97%); 0.72	0.04
Follow-up	104.9 (102.7–107.1%)	104.8 (102.3–107.4%)	−0.04 (1.70)	(−3.39–3.31%); 0.98	−0.01
GSE					
Baseline	21.1 (20.0–22.2%) *n* = 126	21.0 (20.0–21.9%) *n* = 127	–	–	–
Treatment cessation	24.4 (23.6–25.3%)	23.3 (22.3–24.2%)	−1.19 (0.66)	(−2.49–0.10%); 0.07	−0.10
Follow-up	24.4 (23.4–25.5%)	24.7 (23.5–25.8%)	0.23 (0.78)	(−1.30–1.77%); 0.77	0.05
EQ-5D-5L					
Baseline	0.50 (0.45–0.55%) *n* = 126	0.51 (0.46–0.56%) *n* = 124	–	–	–
Treatment cessation	0.62 (0.58–0.66%)	0.57 (0.53–0.62%)	−0.05 (0.03)	(−1.11–0.13%); 0.12	−0.20
Follow-up	0.64 (0.60–0.69%)	0.64 (0.59–0.69%)	−0.004 (0.03)	(−0.07–0.06%); 0.81	0
EQ VAS					
Baseline	53.8 (49.9–57.7%) *n* = 121	50.5 (46.9–54.1%) *n* = 124	–	–	–
Treatment cessation	59.6 (56.0–63.3%)	55.9 (51.9–59.9%)	−3.7 (2.8)	(−9.2–1.7%); 0.18	−0.17
Follow-up	60.1 (56.6–65.2%)	59.2 (54.7–63.7%)	−1.7 (3.1)	(−7.9–4.5%); 0.60	−0.04
WHO-5					
Baseline	33.3 (30.2–36.4%) *n* = 126	30.6 (27.6–33.5%) *n* = 127	–	–	–
Treatment cessation	40.3 (37.0–43.6%)	40.6 (36.9–44.3%)	0.35 (2.50)	(−4.57–5.28%); 0.89	0.02
Follow-up	43.4 (39.4–47.5%)	44.0 (39.3–48.7%)	0.60 (3.17)	(−5.65–6.86%); 0.85	0.02
R-GPTS—Ideas of Persecution					
Baseline	15.1 (13.5–16.6%) *n* = 126	14.9 (13.2–16.6%) *n* = 128	–	–	–
Treatment cessation	8.3 (7.1–9.5%)	8.7 (7.4–10.0%)	1.00^a^ (0.28)	(0.57–1.76%)^a^; 0.99	0^b^
Follow-up	7.9 (6.4–9.3%)	8.4 (6.8–10.1%)	0.51 (1.11)	(−1.67–2.69%); 0.64	0.06
R-GPTS—Ideas of Social Self-reference					
Baseline	13.3 (12.1–14.4%) *n* = 126	14.4 (13.2–15.6%) *n* = 128	–	–	–
Treatment cessation	8.7 (7.8–9.6%)	8.9 (8.0–9.9%)	0.25 (0.67)	(−1.07–1.56%); 0.71	0.04
Follow-up	8.2 (7.1–9.3%)	8.9 (7.8–10.1%)	0.75 (0.80)	(−0.84–2.33%); 0.35	0.11
GPTS total					
Baseline	85.2 (80.7–89.6%) *n* = 126	86.3 (81.3–91.0%) *n* = 128	–	–	–
Treatment cessation	63.1 (59.6–66.5%)	65.1 (61.4–68.8%)	2.06 (2.57)	(−3.02–7.13%); 0.43	0.10
Follow-up	61.6 (57.2–65.9%)	64.5 (59.8–69.3%)	2.94 (3.31)	(−3.59–9.48%); 0.38	0.11
R-GPTS total					
Baseline	28.3 (25.9–30.8%) *n* = 126	29.3 (26.6–31.9%) *n* = 128	–	–	–
Treatment cessation	17.0 (15.1–18.8%)	17.6 (15.6–19.6%)	0.65 (1.38)	(−2.06–3.37%); 0.64	0.05
Follow-up	16.1 (13.9–18.4%)	17.3 (14.8–19.8%)	1.14 (1.71)	(−2.24–4.51%); 0.51	0.10
CSQ					
Treatment cessation	26.9 (26.2–27.6%) *n* = 112	26.1 (25.4–26.9%) *n* = 100	0.26 (0.54)	(−0.80–1.32%); 0.62	0.19

Between-group adjusted mean difference after adjusting for biological sex assigned at birth, study site and dichotomized symptom severity of GPTS subscale Ideas of Persecution (≥45 or <45 at baseline), along with the estimated effect size. All analyses are conducted without adjustment for baseline imbalances. All analyses are linear regression models based on the ITT principle and handled with multiple imputations. All analyses are adjusted for baseline measurement of each outcome, except for the Client Satisfaction Questionnaire (CSQ) that was not administered at baseline. For CSQ, a linear regression model adjusted for biological sex assigned at birth, study site and dichotomized symptom severity of GPTS subscale Ideas of Persecution (≥45 or <45 at baseline) was used and the number of observations in the analysis was 212. Values are presented as *n* (*n*–*n*%), indicating the mean value and its 95% CI, and *n* = indicates the number of observations. Time points: treatment cessation, mean = 4.5 months (95% CI 4.3–4.6) after baseline; follow-up, mean = 10.5 months (95% CI 10.3–10.7) after baseline. BNSS, Brief Negative Symptoms Scale; DACOBS, Davos Assessment of Cognitive Biases Scale; EQ VAS, EuroQol Visual Analog Scale; GSE, General Self-Efficacy scale; SAPS, Scale for the Assessment of Positive Symptoms; SCOPE, Social Cognition Psychometric Evaluation; SFS, Social Functioning Scale; SSPA, Social Skills Performance Assessment; WHO-5, World Health Organization-Five well-being index.

^a^A log transformation was applied, and the reported result is therefore an exponentiated, back-transformed value.

^b^Cohen’s *d* was calculated using the log-transformed mean and s.d. for the two groups.

^c^*P* < 0.05.

^d^The numbers presented are the median and its 25th and 75th percentiles in a non-normal distributed sample.

First assessment at treatment cessation was 29 June 2021 and the final follow-up assessment occurred on 10 August 2024, when we reached a total of 256 participants. At treatment cessation, when the primary outcome was measured, 9 participants (7%) in the VR-CBTp group and 23 participants (18%) in the CBTp group were lost to follow-up, a difference that was statistically significant (*P* = 0.009) (Fig. 1). At follow-up, 21 participants (17%) were lost to follow-up in the VR-CBTp group and 33 (26%) in the CBTp group, which was not statistically significant (*P* = 0.076) (Fig. 1).

Participants in the VR-CBTp group completed an average of 9.0 sessions (95% CI 8.6–9.4) compared to 8.5 sessions in the CBTp group (95% CI 8.0–9.0). In the VR-CBTp group, 24 participants (19%) discontinued treatment (that is, attended 1 to 9 sessions before dropping out) and 102 (81%) completed all sessions. In the CBTp group, 5 (4%) participants attended no sessions, 26 participants (20%) discontinued and 97 (76%) completed full treatment (Fig. 1). As a result, the intervention groups did not statistically differ in the treatment adherence status (*P* = 0.09). Common reasons for discontinuation included a lack of energy or time, therapy unsuitability and, in seven cases, excessive paranoid anxiety about traveling for treatment, despite taxi transport being offered. For further details see Supplementary Table 1.

The mean number of days between baseline and treatment cessation assessments (expected 3 months postbaseline) in the VR-CBTp group was 128 days (95% CI 123–134), and in the CBTp group was 140 days (95% CI 130–149). This represents approximately 4.5 months postbaseline in both groups, rather than the intended 3 months specified in the study design (Supplementary Table 2). The difference in timeline between groups was statistically significant (*P* = 0.049) but was driven by an extreme outlier in the CBTp group. After removing this outlier, the difference was no longer statistically significant (*P* = 0.10) (Supplementary Table 2). The mean number of days between baseline and follow-up (expected 9 months postbaseline under the circumstance that treatment cessation was 3 months postbaseline) in the VR-CBTp group was 309 days (95% CI 302–316) and in the CBTp group it was 322 days (95% CI 310–334), that is, close to 6 months in both groups between treatment cessation and follow-up and in accordance with the study design. The difference between groups was not statistically significant (Supplementary Table 2). No group differences in adjunctive psychosocial treatment were observed (Supplementary Table 3).

### Primary outcomes

**Fig. 2:**
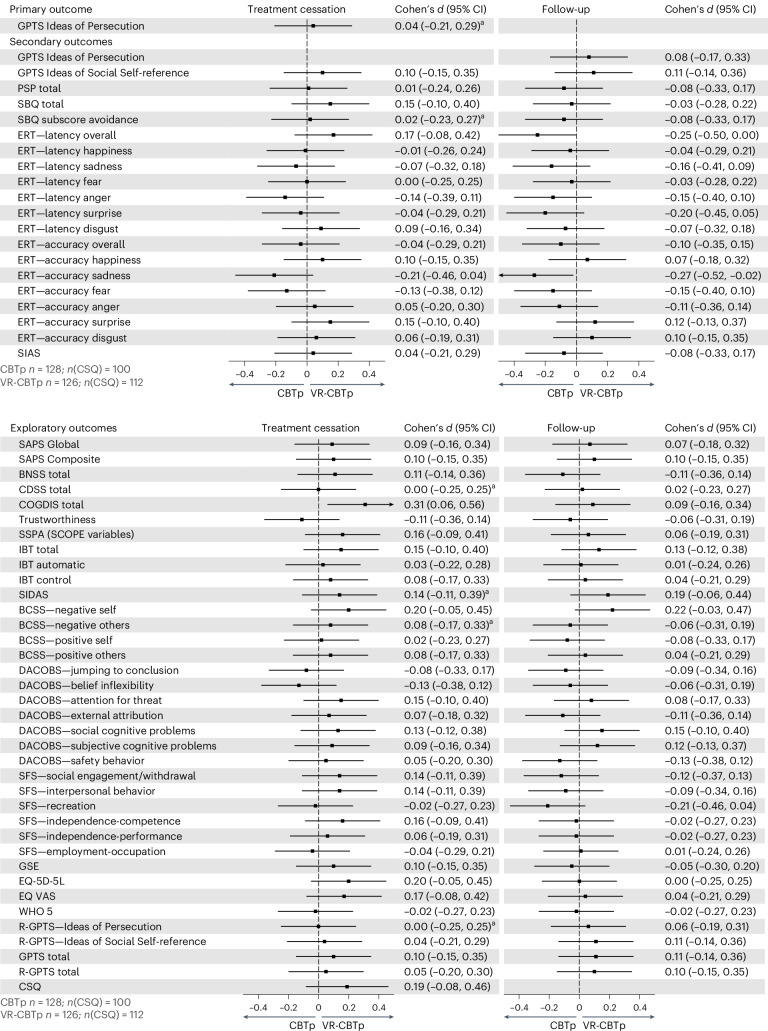
Effect size estimates with 95% CIs on primary, secondary and exploratory outcomes of the primary analyses at treatment cessation and follow-up. The effect sizes (center points) with 95% CIs (error bars) for each outcome are shown. Effect sizes were calculated based on the primary analyses presented in Tables 2, 3 and 4. Using the effect size and number of observations, 95% CIs were calculated. As analyses were handled with multiple imputations, number of observations is a constant throughout all analyses (VR-CBTp: 126; CBTp: 128), except for the CSQ that was not administered at baseline (VR-CBTp: 112; CBTp: 100). Time points: treatment cessation, mean = 4.5 months (95% CI 4.3–4.6) after baseline; follow-up, mean = 10.5 months (95% CI 10.3–10.7) after baseline. ^a^Cohen’s *d* has been calculated by using the log-transformed mean and s.d. values for the two groups.

A forest plot showing the estimated effect sizes with 95% CIs for the primary, secondary and exploratory outcomes of the primary analyses at treatment cessation and follow-up is presented in Fig. 2. As residual plots indicated a non-normal distribution on the primary outcome, the GPTS subscale Ideas of Persecution at treatment cessation, we applied log transformation, which improved model fit. There was not a statistically significant between-group difference on the primary outcome. The exponentiated log-transformed effect estimate showed a 2% lower (that is better) score in the VR-CBTp group (95% CI 11% lower for CBTp to 17% lower for VR-CBTp; Cohen’s *d* = 0.04; *P* = 0.77) (Table 3). The Mann–Whitney *U* nonparametric test further confirmed the nonsignificant finding (*P* = 0.70) (Supplementary Table 4). The lack of significance was maintained in the sensitivity analysis with adjustment for baseline imbalances (Supplementary Table 5). The time-by-group interaction was also not statistically significant (*P* = 0.82).

**Table 3:** Between-group adjusted mean difference and the estimated effect size on the primary outcome

	VR-CBTp mean (95% CI)	CBTp mean (95% CI)	Adjusted mean difference (standard error)	95% CI for adjusted mean difference; *P* value	Cohen’s *d*
GPTS—Ideas of Persecution					
Baseline	41.1 (38.6–43.7%) *n* = 126	41.1 (38.2–43.9%) *n* = 128	–	–	–
Treatment cessation	29.7 (27.8–31.7%)	30.8 (28.8–32.9%)	1.02^a^ (0.19)	(0.89 to 1.17%)^a^; 0.77	0.04^b^

Between-group adjusted mean difference after adjusting for biological sex assigned at birth, study site and dichotomized symptom severity of GPTS subscale Ideas of Persecution (≥45 or <45 at baseline), along with the estimated effect size. The analysis is a linear regression model based on the ITT principle and handled with multiple imputations. Analysis is adjusted for baseline measurement of GPTS subscale Ideas of Persecution. Analysis is conducted without adjustment for baseline imbalances. Values are presented as *n* (*n*–*n*%) indicating the mean value and its 95% CI and *n* = indicates the number of observations at baseline. Time points: treatment cessation, mean = 4.5 months (95% CI 4.3–4.6) after baseline; follow-up, mean = 10.5 months (95% CI 10.3–10.7) after baseline.

^a^Due to the non-normal distribution of the residual plots, a log transformation was applied, which improved the model fit; the reported result is therefore an exponentiated, back-transformed value.

^b^Cohen’s *d* has been calculated by using the log-transformed mean and s.d. values for the two groups.

### Secondary outcomes

There was no statistically significant between-group difference on the GPTS subscale Ideas of Persecution at follow-up (adjusted mean difference 1.20, 95% CI −2.43 to 4.83; Cohen’s *d* = 0.08; *P* = 0.52) (Fig. 2 and Table 4). Similarly, no statistically significant between-group differences were found at any time point for the GPTS subscale Ideas of Social Self-reference, the Safety Behavior Questionnaire (SBQ) total and avoidance scores^30^, the Personal and Social Performance Scale (PSP) total score^31^ or the Social Interaction Anxiety Scale (SIAS)^32^ (Fig. 2 and Table 4). The SBQ avoidance analysis was log transformed to improve model fit at treatment cessation and the following nonparametric Mann–Whitney *U* test supported no statistically significant findings observed in this analysis (*P* = 0.89) (Supplementary Table 4). Furthermore, no statistically significant difference was seen in sensitivity analyses with adjustment for baseline imbalances in any of the abovementioned outcome measures (Supplementary Table 6). On the Cambridge Neuropsychological Test Automated Battery Emotion Recognition Task (ERT)^33,34^, no statistically significant differences were observed at treatment cessation. However, CBTp showed statistically significant better ERT sadness accuracy at follow-up both in the primary analysis (adjusted mean difference 0.85, 95% CI 0.06–1.63; Cohen’s *d* = 0.27; *P* = 0.034) and in the sensitivity analysis with adjustment for baseline imbalances (Fig. 2, Table 4 and Supplementary Table 6). Additionally, the CBTp group had at follow-up, in the sensitivity analysis with adjustment for baseline imbalances, a shorter overall latency than VR-CBTp (adjusted mean difference −365.4 ms, 95% CI: −724.1 to −6.7 ms; Cohen’s *d* = −0.25; *P* = 0.046) (Supplementary Table 6). Similarly, all time-by-group interactions were not statistically significant except for ERT overall latency (*P* = 0.005) and ERT sadness accuracy (*P* = 0.01).

**Table 4:** Between-group adjusted mean difference and the estimated effect size on secondary outcomes

	VR-CBTp mean (95% CI)	CBTp mean (95% CI)	Adjusted mean difference (standard error)	95% CI for adjusted mean difference; *P* value	Cohen’s *d*
GPTS—Ideas of Persecution					
Follow-up	29.2 (26.8–31.8%)	30.4 (27.8–33.0%)	1.20 (1.84)	(−2.43–4.83%); 0.52	0.08
GPTS—Ideas of Social Self-reference					
Baseline	44.0 (41.7–46.3%) *n* = 126	45.2 (43.0–47.4%) *n* = 128	–	–	–
Treatment cessation	33.3 (31.5–35.1%)	34.4 (32.4–36.3%)	1.10 (1.33)	(−1.53–3.72%); 0.41	0.10
Follow-up	32.5 (30.3–34.7%)	33.9 (31.6–36.3%)	1.46 (1.65)	(−1.81–4.72%); 0.38	0.11
PSP total					
Baseline	42.7 (40.5–44.9%) *n* = 126	45.3 (43.0–47.5%) *n* = 128	–	–	–
Treatment cessation	49.0 (47.7–50.2%)	48.9 (47.5–50.2%)	−0.07 (0.94)	(−1.92–1.78%); 0.92	−0.01
Follow-up	50.9 (49.0–52.9%)	51.89 (49.5–54.2%)	0.94 (1.57)	(−2.15–4.04%); 0.55	0.08
SBQ total					
Baseline	55.1 (51.0–59.2%) *n* = 126	54.2 (50.0–58.4%) *n* = 128	–	–	–
Treatment cessation	36.3 (33.7–39.0%)	38.6 (35.8–41.5%)	2.28 (1.97)	(−1.62–6.18%); 0.25	0.15
Follow-up	34.6 (31.1–38.1%)	34.0 (29.8–38.3%)	−0.56 (0.84)	(−5.97–4.86%); 0.84	−0.03
SBQ subscore avoidance					
Baseline	38.9 (37.1–40.8%) *n* = 126	38.3 (36.4–40.2%) *n* = 128	–	–	–
Treatment cessation	9.3 (8.1–10.4%)	9.3 (8.0–10.6%)	1.06^a^ (0.30)	(0.59–1.90%)^a^; 0.85	0.02^b^
Follow-up	8.9 (7.5–10.3%)	8.1 (6.4–9.9%)	−0.77 (1.11)	(−2.97–1.44%); 0.49	−0.08
ERT—latency overall					
Baseline	3,219 (2,833–3,604%) *n* = 124	2,882 (2,568–3,196%) *n* = 126	–	–	–
Treatment cessation	2,307 (2,106–2,508%)	2,512 (2,297–2,726%)	204.5 (147.9)	(−87.2–496.2%); 0.17	0.17
Follow-up	2,341 (2,073–2,609%)	1,993 (1,770–2,216%)	−348.3 (176.1)	(−696.6 to −0.04%); 0.05	−0.25
ERT—latency happiness					
Baseline	1,170 (1,072–1,267%) *n* = 124	1,077 (1,027–1,127%) *n* = 126	–	–	–
Treatment cessation	1,009 (946–1,072%)	1,007 (944–1,072%)	−1.86 (45.2)	(−91.1–87.4%); 0.97	−0.01
Follow-up	1,038 (959–1,117%)	1,020 (939–1,100%)	−18.8 (58.4)	(−134.4–96.8%); 0.75	−0.04
ERT—latency sadness					
Baseline	2,116 (1,870–2,363%) *n* = 124	1,978 (1,832–2,123%) *n* = 126	–	–	–
Treatment cessation	1,736 (1,627–1,846%)	1,693 (1,582–1,803%)	−43.6 (77.7)	(−196.8–109.7%); 0.58	−0.07
Follow-up	1,775 (1,587–1,963%)	1,612 (1,432–1792%)	−162.8 (132.4)	(−424.6–99.1%); 0.22	−0.16
ERT—latency fear					
Baseline	2,120 (1,880–2,360%) *n* = 124	2,104 (1,898–2,309%) *n* = 126	–	–	–
Treatment cessation	1,848 (1,713–1,984%)	1,848 (1,699–1,998%)	−0.16 (102.0)	(−201.4–201.1%); 1.00	0
Follow-up	1,677 (1,533–1,822%)	1,651 (1,506–1,797%)	−26.2 (104.5)	(−232.9–180.5%); 0.80	−0.03
ERT—latency anger					
Baseline	1,452 (1,312–1,592%) *n* = 124	1,355 (1,266–1,443%) *n* = 126	–	–	–
Treatment cessation	1,300 (1,215–1,385%)	1,231 (1,141–1,320%)	−69.3 (62.2)	(−191.9–53.3%); 0.27	−0.14
Follow-up	1,321 (1,211–1,430%)	1,224 (1,109–1,339%)	−96.8 (80.7)	(−256.4–62.8%); 0.23	−0.15
ERT—latency surprise					
Baseline	1,336 (1,203–1,449%) *n* = 124	1,222.8 (1,140.2–1,305.3%) *n* = 126	–	–	–
Treatment cessation	1,135 (1,058–1,211%)	1,117 (1,040–1,195%)	−17.64 (55.73)	(−127.55–92.27%); 0.75	−0.04
Follow-up	1,118 (1,045–1,192%)	1,035 (962–1,108%)	−83.29 (53.37)	(−188.73–22.15%); 0.12	−0.20
ERT—latency disgust					
Baseline	2,145 (1,939–2,351%) *n* = 124	1,960 (1,804–2,116%) *n* = 126	–	–	–
Treatment cessation	1,686 (1,569–1,802%)	1,745 (1,619–1,871%)	59.6 (87.8)	(−113.6–232.9%); 0.50	0.09
Follow-up	1,631 (150–1,764%)	1,576 (1,449–1,703%)	−54.7 (92.3)	(−237.0–127.6%); 0.55	−0.07
ERT—accuracy overall					
Baseline	56.6 (54.8–58.3%) *n* = 124	56.1 (54.5–57.6%) *n* = 126	–	–	–
Treatment cessation	57.8 (56.4–59.3%)	58.1 (56.6–59.5%)	0.23 (1.03)	(−1.79–2.26%); 0.82	0.04
Follow-up	57.6 (56.1–59.0%)	58.5 (57.0–60.1%)	0.94 (1.05)	(−1.14–3.01%); 0.37	0.10
ERT—accuracy happiness					
Baseline	11.4 (11.0–11.7%) *n* = 124	11.5 (11.2–11.9%) *n* = 126	–	–	–
Treatment cessation	11.7 (11.4–12.1%)	11.5 (11.1–11.8%)	−0.26 (0.25)	(−0.75–0.23%); 0.30	−0.10
Follow-up	11.7 (11.3–12.1%)	11.5 (11.0–12.0%)	−0.14 (0.32)	(−0.78–0.50%); 0.67	−0.07
ERT—accuracy sadness					
Baseline	10.1 (9.5–10.6%) *n* = 124	9.4 (8.8–9.9%) *n* = 126	–	–	–
Treatment cessation	9.5 (9.0–9.9%)	10.1 (9.6–10.6%)	0.60 (0.35)	(−0.09–1.29%); 0.09	0.21
Follow-up	9.7 (9.1–10.2%)	10.5 (10.0–11.0%)	0.85 (0.40)	(0.06–1.63%); 0.03^c^	0.27
ERT—accuracy fear					
Baseline	6.3 (5.8–6.9%) *n* = 124	6.1 (5.6–6.7%) *n* = 126	–	–	–
Treatment cessation	6.7 (6.1–7.2%)	7.1 (6.5–7.6%)	0.40 (0.40)	(−0.39–1.19%); 0.32	0.13
Follow-up	6.4 (5.7–7.0%)	7.0 (6.3–7.7%)	0.62 (0.49)	(−0.35–1.58%); 0.21	0.15
ERT—accuracy anger					
Baseline	7.9 (7.4–8.4%) *n* = 124	7.9 (7.5–8.3%) *n* = 126	–	–	–
Treatment cessation	8.4 (8.1–8.8%)	8.4 (8.0–8.7%)	−0.03 (0.26)	(−0.54–0.47%); 0.90	−0.05
Follow-up	8.4 (7.9–8.9%)	8.7 (8.2–9.2%)	0.34 (0.36)	(−0.37–1.05%); 0.34	0.11
ERT—accuracy surprise					
Baseline	11.4 (11.1–11.8%) *n* = 124	11.4 (11.1–11.7%) *n* = 126	–	–	–
Treatment cessation	11.5 (11.2–11.9%)	11.2 (10.9–11.6%)	−0.32 (0.26)	(−0.83–0.19%); 0.22	−0.15
Follow-up	11.4 (11.0–11.8%)	11.1 (10.7–11.6%)	−0.24 (0.31)	(−0.86–0.39%); 0.46	−0.12
ERT—accuracy disgust					
Baseline	9.4 (8.8–10.1%) *n* = 124	9.8 (9.1–10.4%) *n* = 126	–	–	–
Treatment cessation	10.0 (9.5–10.6%)	9.8 (9.3–10.4%)	−0.20 (0.40)	(−0.99–0.58%); 0.61	−0.06
Follow-up	10.0 (9.5–10.6%)	9.7 (9.1–10.2%)	−0.38 (0.39)	(−1.15–0.38%); 0.33	−0.10
SIAS					
Baseline	46.8 (44.3–49.4%) *n* = 126	47.8 (45.2–50.5%) *n* = 127	–	–	–
Treatment cessation	41.7 (39.7–43.7%)	42.2 (40.0–44.5%)	0.52 (1.53)	(−2.50–3.53%); 0.74	0.04
Follow-up	39.6 (37.1–42.1%)	38.4 (35.6–41.2%)	−1.21 (1.88)	(−4.91–2.50%); 0.52	−0.08

Between-group adjusted mean difference after adjusting for biological sex assigned at birth, study site and dichotomized symptom severity of GPTS subscale Ideas of Persecution (≥45 or <45 at baseline), along with the estimated effect size. All analyses are linear regression models based on the ITT principle and handled with multiple imputations. All analyses are adjusted for baseline measurement of each outcome. All analyses are conducted without adjustment for baseline imbalances. Values are presented as *n* (*n–n*%), indicating the mean value and its 95% CI; *n* = indicates the number of observations. Time points: treatment cessation, mean = 4.5 months (95% CI 4.3–4.6) after baseline; follow-up, mean = 10.5 months (95% CI 10.3–10.7) after baseline.

^a^A log transformation was applied and the reported result is therefore an exponentiated, back-transformed value.

^b^Cohen’s *d* has been calculated by using the log-transformed mean and s.d. values for the two groups.

^c^*P* < 0.05.

### Safety

Cybersickness was measured using the Simulation Sickness Questionnaire^35^, which was administered in the VR-CBT group during sessions 1 and 2. We used an unweighted approach to calculate total scores. Mean total score at session 1 was 6.75 (95% CI 5.38–8.12) and mean total score at session 2 was 6.10 (95% CI 4.80–7.40). Total scores between 5 and 10 are considered indicative of ‘minimal symptoms’.

Serious adverse events were continuously monitored throughout the trial and reported to the Principal Investigator (PI), the lead therapist, the data monitoring committee and the research ethics committee. Neither deaths nor violent incidents involving law enforcement were reported during the study, and there were no instances in which participation in our study was linked to a suicide attempt. In terms of suicide attempts unrelated to the study, two attempts were reported in the VR-CBTp group and three in the CBTp group from baseline to treatment cessation, and from treatment cessation to follow-up, the VR-CBTp group had no attempts whereas the CBTp group had five. A total of 17 (13.6%) participants in the VR-CBTp group were hospitalized from baseline to treatment cessation, while in the CBTp group there were 15 (11.7%). Similarly, from treatment cessation to follow-up, 27 participants (21.6%) in the VR-CBTp group were hospitalized, while 20 participants (15.6%) in the CBTp group were hospitalized.

The proportion of participants who self-harmed in the past week was seven (6.3%) for the VR-CBTp group at treatment cessation whereas it was six (4.7%) for the CBTp group. At follow-up, the distribution was four (3.2%) for the VR-CBTp group and four (3.1%) for the CBTp group.

### Exploratory outcomes

Exploratory outcomes revealed a statistically significant between-group difference on one measure at treatment cessation in the primary analyses (Fig. 2 and Table 1). The VR-CBTp group demonstrated a lower total score in the Cognitive Disturbances Scale (COGDIS)^36^ (adjusted mean difference 2.58, 95% CI 0.55–4.62; Cohen’s *d* = 0.31; *P* = 0.013). However, this was not sustained in the sensitivity analysis adjusting for baseline imbalances (Supplementary Table 7). The Calgary Depression Symptom Scale (CDSS)^37^, the Suicidal Ideation Attributes Scale (SIDAS)^38^ and the Brief Core Schema Scale: belief about self and others (BCSS)^39^ subscale, Negative Core Beliefs of Others, were log transformed at treatment cessation, which improved their model fit’s. None of the three outcomes found statistically significant between-group differences in the log-transformed linear regression model but the following Mann–Whitney *U* test showed a statistically significant difference in the case of the BCSS subscale, Negative Core Beliefs of Others, in favor of the VR-CBTp group (CDSS *P* = 0.54, SIDAS *P* = 0.10), BCSS subscale, Negative Core Beliefs of Others (*P* = 0.04) (Supplementary Table 4).

No other exploratory outcomes showed statistically significant differences between groups neither at treatment cessation nor at follow-up, both without and with adjustment for baseline imbalances (Fig. 2, Table 1 and Supplementary Table 7). All time-by-group interactions were not statistically significant, except for the COGDIS total score (*P* = 0.04).

### Sensitivity analyses

We conducted a sensitivity analysis on GPTS using the revised GPTS (R-GPTS)^40^. At both treatment cessation and follow-up, no statistically significant differences were observed between groups for the R-GPTS subscales, Ideas of Persecution and Ideas of Social Self-reference, either without or with adjustment for baseline imbalances (Table 1 and supplementary Table 7). The R-GPTS Ideas of Persecution subscale was log transformed at treatment cessation to improve model fit, and the following Mann–Whitney *U* test further supported that there was no between-group difference (*P* = 0.76) (Supplementary Table 4). Additionally, the time-by-group interaction was not statistically significant.

Complete-case-only analyses yielded results largely consistent with our primary intention-to-treat (ITT) analyses and their sensitivity analyses adjusted for baseline imbalances, with one exception. ERT accuracy sadness showed a newly emerging statistically significant difference at treatment cessation in favor of CBTp in the adjusted analysis (*P* = 0.03). For full details, see Supplementary Tables 8–13.

Per-protocol analyses diverged from the primary ITT analyses and its sensitivity analyses on few outcome measures. The previously observed statistically significant difference in ERT overall latency at follow-up (in the ITT sensitivity analysis adjusted for baseline imbalances) was no longer evident in the per-protocol analyses. By contrast, ERT accuracy surprise showed a statistically significant difference at treatment cessation in the adjusted per-protocol analysis, favoring VR-CBTp (*P* = 0.032). Additionally, the EuroQol five-dimensions five-level questionnaire (EQ-5D-5L)^41^ revealed a statistically significant difference at treatment cessation in the unadjusted per-protocol analysis, also in favor of VR-CBTp (*P* = 0.025). For full details, see Supplementary Tables 14–19.

### Post hoc analyses

The amount of exposure in the two groups and the quality of exposure was investigated using a Mann–Whitney *U* test, where missing data were handled by imputing 0. Missing data were 16.3% in the VR-CBTp group and 23.7% in the CBTp group. The total exposure time during the treatment course in the two groups were statistically significantly different in favor of VR-CBTp (*P* < 0.001). Further, the quality of exposure was statistically significantly better in the VR-CBTp group (*P* = 0.035) (Supplementary Table 20).

Presence in the VR environment was measured by the Igroup Presence Questionnaire (IPQ)^42^, which was administered in the VR-CBT group during sessions 1 and 9. IPQ consists of three subscales, Spatial presence, Involvement and Realness, with scores ranging from 0 to 6 for each subscale. Mean score for Spatial presence at session 1 was 4.30 (95% CI 4.07–4.44) and 4.42 (95% CI 4.24–4.60) at session 9. Mean score for Involvement at session 1 was 3.31 (95% CI 3.05–3.47) and 3.35 (95% CI 3.19–3.52) at session 9. Mean score for Realness at session 1 was 3.15 (95% CI 2.92–3.37) and 3.36 (95% CI 3.15–3.57) at session 9. All scores are considered at least ‘sufficient’.

Given that both groups improved rather than remained unchanged, we conducted a post hoc analysis of within-group effects. As the treatment cessation data were not normally distributed, they were log transformed. VR-CBTp produced a large reduction (Cohen’s *d* = 0.97; 29.8% reduction), whereas CBTp yielded a moderate reduction (*d* = 0.75; 26.3% reduction). These effect sizes persisted at follow-up (VR-CBTp: *d* = 0.86; CBTp: *d* = 0.68). Because effect sizes at different time points were derived on different scales, their precise values are not directly comparable. A similar pattern emerged on the R-GPTS Ideas of Persecution subscale.

## Discussion

This study conducted a direct comparison of VR-CBTp and CBTp in patients with SSD. We hypothesized that VR-CBTp would be superior to CBTp on our primary outcome measure being the GPTS subscale Ideas of Persecution at treatment cessation. Contrary to expectations, our results did not support this hypothesis. Our primary ITT analysis without adjustment for baseline imbalances was followed by sensitivity analyses, including adjustments for baseline imbalances, complete-case-only analyses and per-protocol analyses, none of which revealed any statistically significant differences between groups for the primary outcome.

Our finding contrasts with a previous randomized controlled trial, which reported larger effect sizes for VR-CBTp over wait-list control^25^ exceeding those typically found for CBTp under similar conditions^16^. The lack of difference between VR-CBTp and CBTp in our randomized controlled trial emphasizes the importance of evaluation of new interventions not only against passive or enhanced TAU comparators but also against current best practices. Without such comparisons, evidence may overstate the advantages of new therapies, leading to premature clinical adoption or approval. Our findings are consistent with research in related fields, such as social anxiety disorders, where VR-based interventions outperform passive controls but do not consistently exceed the effects of active treatments such as in vivo exposure^43^. This finding suggests that while VR may offer logistical and engagement advantages, its clinical impact may not surpass standard methods, particularly when the comparator is a gold-standard CBTp.

We found a higher proportion of exposure in the VR-CBTp group compared to CBTp and participants in the VR-CBTp group rated the quality of exposure statistically significantly higher. While we believed these factors would enhance treatment efficacy, our findings did not support this. This contrasts with meta-analysis evidence^13^ suggesting that a stronger behavioral component in CBT for psychosis is associated with larger effects.

However, several limitations in our exposure data collection should be acknowledged. The VR-CBTp did not include structured homework. Participants were encouraged to attempt similar exposures in real-world settings, but it is unclear to what extent transfer of learning occurred from VR exposure to real-world situations. In contrast, the CBTp incorporated scheduled between-session homework, although adherence data were not collected. It is plausible that the CBTp group engaged more consistently in real-world exposure, limiting the difference in exposure-based learning between the two interventions. An ongoing qualitative study on participants’ and therapists’ experiences may offer insights into exposure engagement across both treatment arms.

Our study may have encountered a ‘floor effect’ on the primary outcome measure, the GPTS subscale Ideas of Persecution. Posttreatment mean scores in our sample ranged from 29.2 to 30.8, which is comparable to the mean score of 28.7 reported previously by Freeman et al.^40^ in a sample with nonpsychotic mental health conditions, and notably lower than the scores of 38.1 and 58.7 observed in individuals with psychotic disorders and persecutory delusions in the same study. Furthermore, our R-GPTS subscale scores for Ideas of Persecution remained at or below 8.7 across all posttreatment time points for both groups. This should be considered in light of the recommendation by Freeman et al.^40^ that a score of ≥11 is used to differentiate cases with persecutory delusions from nonclinical cases. Given these benchmarks, further symptom reduction on the GPTS or R-GPTS in our SSD population appears unlikely, reinforcing the possibility of a floor effect limiting additional treatment-related gains.

Regarding our secondary outcome measures, we unexpectedly found CBTp to outperform VR-CBTp on specific secondary outcomes, ERT overall latency and sadness accuracy at follow-up. This finding is counterintuitive as facial emotion recognition was not a target in CBTp, and the hypothesis was that VR-CBTp would yield greater benefits in social cognitive aspects, as VR enabled more social encounters (that is, encounters with avatars displaying different emotions). Only 2 out of 28 ERT measurements showed a statistically significant between-group difference, suggesting the possibility that these findings may be due to chance.

Taken together, these findings carry implications for clinical practice and regulatory evaluation. Although VR-CBTp may enhance certain therapeutic processes such as engagement and therapist control over stimuli, our study does not support its superiority over CBTp in clinical outcomes for paranoia. This underscores the need for further research into whether VR-CBTp can be optimized in its delivery to enhance efficacy, and it highlights the need to consider other factors such as cognitive interventions^44^, therapeutic alliance^45^ and patient preferences^46^, all of which may influence treatment outcomes. Therefore, VR-CBTp should be considered as a complementary or alternative option, particularly in contexts where CBTp is less feasible or less effective such as in patients with prominent negative symptoms or severe avoidance due to paranoid anxiety^23^. In such cases, VR-CBTp may improve access or adherence, while offering comparable clinical efficacy.

While we focused on exposure given its central role in reducing safety behaviors linked to paranoia, it is likely that other mechanisms of change and additional mediators also contributed to treatment effects^27,44^. Although a detailed examination of these mechanisms falls beyond the scope of the present Article, future research should investigate potential mediators within each intervention and assess whether mechanisms differ across treatments. This could guide optimization of future therapies. Additionally, our broad range of outcome and baseline measures, including sociodemographic characteristics, enables future moderator and predictor analyses to help identify which patients benefit most from each approach, ultimately supporting the development of more personalized interventions.

We speculate that several technological enhancements could further improve the efficacy of VR-CBTp: (1) site-specific exposure, allowing patients to engage with simulations tailored to their home or local environment; (2) features addressing bizarre delusions, such as the creation of supernatural entities or simulation of perceptual disturbances; and (3) adjustable levels of realism to accommodate individual differences in immersive capacity. Given the rapid pace of technological advancement, for instance, the exponential advancement of artificial intelligence-driven solutions, definitive conclusions about the long-term potential of VR in clinical care may be premature; however, its promise is likely to expand over time.

Turning to the strengths of the study, one notable aspect is the selection of a control treatment, which we consider the current gold standard: symptom-specific CBTp with case-formulation, targeting paranoia, and in vivo exposure provided when deemed beneficial and feasible. This optimized version of CBTp extends beyond what is typically provided as standard treatment, at least in a Danish context. Moreover, unlike usual clinical settings in Denmark, the psychologists involved in the study received specialized training in both treatment manuals, along with supervision throughout the study. As such, our control treatment is likely to be more effective than the TAU offered in outpatient care settings.

Another strength lies in the pragmatic study design, which enhances its relevance for clinical implementation. Both treatments consisted of 10 sessions, aligning with similar interventions at least in the Danish clinical practice. We also minimized selection bias by including participants regardless of antipsychotic medication use, and medication changes did not lead to exclusion. Participants with alcohol or other substance misuse were also eligible. As a result, our sample closely reflects the patient population seen in outpatient care settings, although patients unable to leave their homes were not included. All therapists in the North Denmark Region were employed in outpatient care settings and participated in the study on a part-time basis. Furthermore, the therapist who delivered most treatment courses had limited experience with CBT and psychosis-therapy interventions. This finding suggests that the interventions evaluated in our study could be feasibly implemented in current clinical settings, by less experienced therapists, if appropriate training and supervision are provided. Finally, interventions were feasible and well-received by participants. Participants reported moderate to high satisfaction with both VR-CBTp and CBTp, and acceptability was rated as high, with 81.0% and 75.8% of participants in each group, respectively, completing all sessions.

Several limitations must be considered when interpreting the findings of the study. First, the absence of an inactive control group complicates the interpretation of the lack of difference between the VR-CBTp and CBTp groups on the primary outcome. Especially the substantial within-group reduction in paranoia should be interpreted with caution as factors such as placebo, regression to the mean and spontaneous remission may have contributed to the observed improvements. However, Pot-Kolder et al.^25^ found a moderate between-group effect size (Cohen’s *d* = 0.70) on the GPTS subscale Ideas of Persecution when comparing VR-CBTp to a waiting list in a sample with comparable diagnoses and baseline severity of Ideas of Persecution, using an intervention similar to ours. This was a secondary outcome in their study, while their primary outcome, social participation, did not show a statistically significant between-group difference. This finding suggests that symptom reductions observed in our study may not be solely attributable to the natural course of paranoia, but rather to treatment effects, despite the lack of a statistically significant difference between groups in our trial.

Second, we did not specify a fixed amount of exposure in the CBTp treatment manual, which likely contributed to the observed difference in exposure time between treatment arms. However, a greater flexibility may have allowed CBTp to address a broader range of cognitive behavioral targets^44^ beyond those emphasized in VR-CBTp.

Third, we did not conduct any inter-rater reliability assessments for exploratory outcomes, which may limit confidence in the consistency of these findings. We did, however, conduct internal supervision upon request on all outcome measures throughout the trial.

Fourth, we also had to exclude data from five participants who withdrew their consent during the trial. As a result, we are neither able nor permitted to account for their reasons for withdrawal.

Fifth, the study lacked data on ethnicity and migrant status, preventing conclusions about the intervention’s effectiveness for minority populations, even though existing literature shows that CBTp outcomes vary by ethnicity^47^. Similarly, sex and gender were recorded solely in binary biological terms as sex assigned at birth, which limits our ability to explore potential gender differences in treatment outcomes.

Finally, due to the considerable number of outcomes included in the study, we cannot exclude the possibility of Type 1 errors due to the risk of multiplicity. To mitigate this risk, we focused on the primary outcome and adhered to prespecified analytical approaches. However, the potential for false positives remains a consideration in the interpretation of our secondary and exploratory findings.

In conclusion, we did not find a statistically significant difference between VR-CBTp and CBTp on the primary outcome measure, the GPTS subscale Ideas of Persecution, from baseline to treatment cessation. At the current stage of VR technology, VR-CBTp should be considered as a complementary option to standard CBTp, particularly in contexts where it may enhance treatment accessibility, engagement or adherence.

## Methods

### Study design and participants

The study was a two-site, assessor-masked, randomized parallel group superiority trial conducted in the Capital Region of Denmark and the North Denmark Region. Potential participants were referred from their outpatient care setting. These settings primarily included OPUS and F-ACT teams. Study assessors managed the enrollment process.

The study was approved by the Committee on Health Research Ethics of the Capital Region Denmark (H-20048806) and the Danish Data Protection Agency (*P*-2020-823). The final protocol and protocol update have been published in Trials^49,50^. The study was overseen by a trial steering committee, comprising of the PI and therapist, the leader of the study site of North Denmark Region, as well as the leading assessor and therapist from both study sites.

### Eligibility criteria

All referrals were screened for eligibility based on the following inclusion criteria: (1) 18 years or older, (2) diagnosis of an SSD (ICD-10, F20-29), (3) ability to give informed consent (for example, no acute psychotic exacerbation) and (4) a total GPTS score ≥ 40 (that is, the sum score of Ideas of Persecution and Ideas of Social Self-reference). Participants were excluded if (1) they were diagnosed with an organic brain disease, (2) they had an intelligence quotient ≤70 (assessed by medical record), (3) they had the inability to tolerate the assessment process or (4) they did not have an adequate command of spoken Danish or English for engaging in therapy assessed at baseline interview. All participants gave written informed consent. Notably, our trial included individuals with schizotypal disorder, classified within the mild spectrum of schizophrenia in the ICD-10 classification.

We aimed to minimize scheduled changes in antipsychotic medication or psychosocial treatments during the treatment period, but these were not exclusion criteria, as outpatient care providers retained responsibility for participants’ overall treatment. Hospitalizations for acute psychotic episodes led to suspension of project treatment. Discontinuation occurred if a participant opted out or if trial therapist or outpatient clinicians recommended it.

### Randomization and masking

Participants were randomly assigned (1:1) to VR-CBTp plus TAU or CBTp plus TAU with a variable block size created by an independent trial statistician, with no involvement in participant enrollment or trial management, and kept concealed from all study personnel, including assessors. Randomization was conducted following the baseline assessment using a centralized, computer-generated system created by the independent trial statistician. Nonmasked personnel informed participants of their assigned allocation.

Masking of assessors was preserved by separating assessors from therapists, and participants were instructed not to disclose their allocation. If unmasking occurred, patients were reassigned to a different masked assessor. Video recordings of interviews allowed masked assessment to be conducted later if unmasking occurred. If unmasking occurred during a nonrecorded interview, the interview was discontinued, and reassessment was scheduled with another masked assessor. Unmasking occurred altogether five times and remasking was successful in all cases.

### Procedure

The treatment manuals used were Danish adaptations of the VR-CBTp and CBTp manuals^25^ with a key revision being the reduced treatment period from 16 to 10 sessions. All subelements in the original manuals were preserved but condensed. Both treatments consisted of 10 individual sessions, a duration selected to align with previous studies that utilized both a single session and 16 sessions^24,25^, as well as the typical delivery format for similar interventions in Danish clinical settings. Additionally, symptom-specific interventions, particularly those that are digitally enhanced, do not appear to require the 16 sessions or more^51^ that are recommended for standard CBTp courses^52,53^.

Individuals with lived experience of psychosis were involved throughout the study period. Specifically, they provided structured feedback on both treatment protocols, which informed revisions to the therapy manuals to improve their relevance and acceptability. Furthermore, individuals with lived experience contributed to stakeholder engagement activities (for example, meetings with policymakers) and supported dissemination efforts, including public presentations and media communication.

Both VR-CBTp and CBTp were delivered by the same group of psychologists across our two study sites to minimize therapist effect. In the Capital Region of Denmark, three therapists, with 1 to 17 years of CBT experience, conducted 176 treatment courses, while in the North Denmark Region, five therapists, with 4 to 15 years of CBT experience, conducted 78 treatments. The therapist delivering most treatments (90 courses) had 1 year of CBT experience. Most therapists received a 2-day course in both manualized treatments, while two therapists became involved late in the study and received side-by-side training. Ongoing internal consultation was provided weekly during the first year of the trial and biweekly thereafter in the Capital Region of Denmark. In North Denmark Region, internal consultation was not scheduled until 1 year after the initial treatment course had begun. Monthly external online supervision by an international expert in both modalities was conducted during the trial’s first 18 months.

The experimental intervention, VR-CBTp, is a symptom-specific CBTp targeting paranoia, which employs VR as an advanced tool for exposure therapy, building on the foundational principles of CBTp described later on. This intervention therefore comprised core CBTp techniques. The expected difference was based on the assumption that VR-CBTp could provide a more effective and accessible behavioral component of CBTp^49^. Through exposure or behavioral experiments in VR, participants can gradually reduce avoidance and safety behaviors by confronting the triggers of their paranoia within a controlled, safe environment. In theory, this approach enables them to revisit original triggers in real-life, otherwise impossible to confront, and reinterpret them as nonthreatening, potentially facilitating cognitive restructuring. Our treatment manual involves 10 sessions with session 1 lasting 90 min and sessions 2 to 10 lasting 60 min. In session 1, participants are interviewed about their specific paranoid threats, short- and long-term consequences of inappropriate safety behavior are clarified, psychoeducation is provided and participants are introduced to the VR environments. In session 2, case-formulation and treatment goals are established. VR exposure therapy is initiated in session 3, with 15 min of exposure gradually increasing to 20 min to 30 min of exposure in sessions 4 to 9. The final session (session 10) focuses on evaluation of the therapy and planning future therapist-independent therapeutic work. Between sessions, therapists encourage participants to attempt similar exposure exercises in real-life settings to facilitate transfer of learning. The duration of VR exposure in each session is registered by the therapist. The quality of exposure is self-rated by the participant on a Likert scale from 1 to 10. The sense of being present in the immersive VR environment is measured by the IPQ^42^ during sessions 1 and 9. Cybersickness is measured by the Simulation Sickness Questionnaire^35^ during sessions 1 and 2. During VR exposure, both the participant and therapist are present in the same room for the entire therapy session. The participant is fully immersed both auditorily and visually, while the therapist communicates via a headset. Participants can immediately exit the VR environment by removing the headset themselves or by requesting a break, at which point the therapist assist them promptly. While we aim to maintain VR exposure within 20 min, breaks are allowed to accommodate individual tolerance. The VR program used is the CleVR Social Worlds, previously employed in other studies^25,54^. This program comprises an animated universe with five distinct environments—bus, café, shopping street, park and supermarket—typical social situations in everyday life that may trigger paranoia. Participants can walk around in these environments, engage in role-play exercises, test threat beliefs or explore worst-case scenarios. Each situation is customized to suit participants’ individual needs with the difficulty level adjustable from session to session. The program features a comprehensive catalog of animated characters, so-called avatars, which possess a diverse array of characteristics. In addition to gradually increasing exposure time from sessions 4 to 9, therapists adjust key variables within the VR environment, such as the number of people present, social interactions, eye contact and background noises. These adjustments are not predetermined but are made in real time based on the participant’s progress. If paranoid anxiety decreases substantially, the therapist can modify the exposure parameters to maintain an appropriate level of challenge. This individualized approach ensures that exposure remains effective while preventing excessive distress.

The comparator, symptom-specific CBTp, is based on three factors that contribute to the development and maintenance of paranoia: the aberrant salience theory^55^ (random events are perceived as important and/or meaningful), dysfunctional cognitive tendencies (for example, reasoning bias) and consolidating processes (selective attention to threats and safety behaviors). These changeable factors are part of a modified cognitive model developed for paranoia^56,57^. Also in this manual, session 1 lasts for 90 min and session 2 to 10 lasts for 60 min. In session 1, case-formulation and treatment goals are defined. In session 2, the participants receive psychoeducation in cognitive tendencies and are trained in doing cognitive analyses of situations that trigger paranoia. Sessions 3 to 4 explore paranoid beliefs and associated negative automatic thoughts as well as alternative thoughts. In sessions 5 to 8, negative automatic thoughts are challenged, and in vivo exposure and behavioral experiments are planned and conducted if feasible and deemed beneficial according to the individualized case-formulation. Lack of feasibility is often due to practical constraints such as transporting or ensuring the setting is appropriately tailored to the individual’s specific difficulties. If it is challenging for the participant to engage with cognitive interventions, the behavioral component is extended across additional sessions. Session 9 is dedicated to core beliefs about the self and self-esteem, and session 10 focuses on evaluating the therapy and defining future therapist-independent work. Therapists assist participants in planning homework assignments between sessions. The duration of potential in vivo exposure in each session is registered by the therapist. The quality of exposure is self-rated by the participant on a Likert scale from 1 to 10.

OPUS and F-ACT accounted for 98.8% of all TAU received by participants in the trial and their treatment frameworks are therefore briefly described. OPUS provides structured, multidisciplinary care, typically involving regular meetings, every 1 to 2 weeks, with a designated care coordinator. Coordinators are commonly trained nurses, occupational therapists, social workers or psychologists. Supplementary consultations with a medical doctor are available when needed. These sessions do not constitute formal psychotherapy. The core of OPUS care is pharmacological treatment when clinically indicated combined with psychosocial support. Group-based interventions are a standard component, addressing areas such as psychoeducation and self-esteem. Involvement of relatives is actively encouraged and individual therapy is occasionally offered in selected cases. F-ACT adopts a more flexible, need-based model. Meeting intervals with the care coordinator may exceed the 2 weeks for patients in stable conditions. A medical doctor is affiliated with the team and social worker involvement is available when relevant. Compared to OPUS, F-ACT provides group-based and individual therapy less frequently, with an emphasis on tailored support aligned with clinical status.

### Outcomes

The primary outcome was the GPTS subscale Ideas of Persecution (self-report questionnaire, score range 16–80), measured at treatment cessation^6^. The GPTS subscale Ideas of Persecution has demonstrated good psychometric properties overall in large clinical and nonclinical samples but the presence of, for instance, local dependence indicates a potential for measurement errors^40^.

The secondary outcomes of paranoia were GPTS subscale Ideas of Persecution, measured at follow-up, and GPTS subscale Ideas of Social Self-reference, measured at treatment cessation and follow-up^6^.

Other secondary outcomes, all measured at treatment cessation and follow-up, are as follows:

- The SBQ (semi-structured interview)^30^. In the original questionnaire, closed-ended questions are used to uncover common situations that patients with persecutory delusions tend to avoid, as avoidance was the most frequent safety behavior (92%) observed^30^. We decided to include, besides the original open-ended questions, supporting closed-ended questions for the second most frequent safety behavior, in-situation (68%)^30^, as people with lived experiences, who fulfilled eligibility criteria and provided us with feedback, gave us the impression that these behaviors would often be present in our sample but rarely recognized by patients themselves. Consequently, we selected a list consisting of the most common in-situation behaviors mentioned in the original study, which covered four themes: protection, invisibility, vigilance and resistance^30^. We further decided to calculate a subscore of avoidance as this safety behavior is considered especially challenging.
- The PSP (semi-structured interview)^31^.
- The SIAS (self-report questionnaire)^32^.
- The Cambridge Neuropsychological Test Automated Battery ERT long Caucasian version (social cognitive test)^33,34^. We decided to calculate latency and accuracy both as total scores and subscores for each emotion (happiness, sadness, fear, anger, surprise and disgust) as distinct emotions have shown to be differently impaired in SSD^58,59^.

Exploratory outcomes, measured at treatment cessation and follow-up, are as follows:

- SAPS (semi-structured interview)^60^. Items were assessed based on the past month.
- BNSS (semi-structured interview)^61^.
- COGDIS (semi-structured interview)^36^ was included to capture subtle, nonpsychotic anomalous experiences, particularly among participants with schizotypal disorder (F21 diagnosis). While traditionally associated with clinical high-risk groups, the inclusion of COGDIS aimed to provide a more nuanced assessment of symptomatology beyond what was captured by SAPS. One of the COGDIS items, tendencies of unstable self-reference, was given the highest score of six in our study, by default, if participants described paranoia as paranoid ideation (ideas of self-reference or persecution) or delusion on daily basis. We decided on this as less disturbing subtle experiences tend to recede and become obscured when more severe experiences within the same domain emerge^62^.
- CDSS (structured interview)^37^.
- BCSS (self-report questionnaire)^39^.
- GSE (self-report questionnaire)^63^.
- DACOBS (self-report questionnaire)^64^.
- SIDAS (self-report questionnaire)^38^.
- IBT (social cognitive test)^28^. Paradigm was built in E-prime^65^. We used the 24-item version from the SCOPE study and calculated total, automatic and control^28,33^.
- Trustworthiness task (social cognitive test)^48^.
- SFS (self-report questionnaire)^66^. The SFS prosocial activities subscale was excluded due to its sensitivity to COVID-19 restrictions in Denmark (March 2020–February 2022).
- SSPA (semi-structured role play)^33^. We used the SCOPE study version^33^.
- The WHO-5 well-being index (self-report questionnaire)^67^.
- EQ-5D-5L^41^ (self-report questionnaire).

A Big-5 personality traits 25 items 5-point Likert scale (self-report questionnaire)^68^ and TALE (self-report questionnaire)^29^ were measured at baseline.

The CSQ (self-report questionnaire)^69^ was measured at treatment cessation.

The R-GPTS (self-report questionnaire) was measured at treatment cessation and follow-up to conduct a sensitivity analysis on the GPTS^40^.

### Inter-rater reliability and fidelity to treatment manual

Assessments were conducted at baseline, treatment cessation (expected at 3 months postbaseline) and at follow-up (expected at 9 months postbaseline). Trained medical doctors or psychologists conducted the assessments. Internal supervision for assessors on outcome measures was provided monthly during the first 2 years and bimonthly thereafter. Interviews were videotaped to conduct inter-rater reliability ratings on the secondary outcome measures PSP and SBQ. A total of 14 randomly selected interviews were assessed using intraclass correlations with two-way mixed-effects model to evaluate internal consistency. For the total score across both treatment groups, intraclass correlations for single measures were 0.80 for PSP and 0.97 for SBQ corresponding to good and excellent agreement, respectively.

All treatment sessions were audio recorded to assess treatment fidelity. An independent experienced clinical psychologist evaluated fidelity to the treatment manuals by rating seven randomly selected treatment courses from each intervention using the Cognitive Therapy Rating Scale^70^. This scale comprises 11 items, each scored on a range from 0 to 6. The mean score for all 11 items was calculated for each session. For each of the two sets of seven treatment courses, one session was randomly selected from the beginning, middle and end of the course. That is, in total, 2 sets of 21 sessions were evaluated. In the VR-CBTp group, therapists demonstrated ‘good’ to ‘very good’ fidelity with a mean score of 4.4 (95% CI 4.0–4.8). In CBTp, therapists demonstrated ‘good’ to ‘very good’ fidelity to the treatment manual with a mean score of 4.7 (95% CI 4.1–5.2).

### Safety and adverse events

All adverse and serious adverse events were recorded in accordance with the published study protocol. A common reported side effect of VR-CBTp is cybersickness, which resembles motion sickness and typically diminishes with repeated exposure as tolerance develops. To monitor this, cybersickness was systematically assessed during session 1 and 2 in the VR-CBTp group to ensure early detection of any severe or problematic responses to VR.

The following prespecified serious adverse events were actively monitored: (1) hospital admissions, (2) suicide attempts, (3) incidents involving police intervention (regardless of whether the participant is the victim or accused), (4) self-harming behavior and (5) deaths from any cause. Therapist maintained ongoing communication with both participants and their care coordinator throughout the treatment course. Additionally, medical records were reviewed from the time of written informed consent until final follow-up. For participants who discontinued treatment but participated in follow-up assessments or provided access to medical records, monitoring continued according to protocol, with evaluations at 3-months and 9-months postbaseline. Self-harming during the prior week was assessed during the interviews-based follow-up as such events are often underreported in clinical records. Any reported adverse events were reviewed by a safety group consisting of the PI and the PT, and, if relevant, the site coordinator and lead therapist at the North Denmark Region study site. All adverse events, regardless of their relation to the intervention, were reported annually to the Committee on Health Research Ethics of the Capital Region of Denmark, which retained the authority to evaluate whether any events warranted modifications to the study protocol or its continuation. Serious adverse events were reported to the Ethics Committee within 1 week of identification, in accordance with regulatory requirements. Importantly, none of the serious adverse events reported during the study were assessed as related to the intervention. Hospitalizations that occurred during the trial were attributed to external psychosocial stressors, medication adjustments, or, in some cases within the Capital Region, long-term rehabilitative admissions aimed at improving negative symptoms and supporting daily functioning.

### Statistical analyses

The sample size calculation was based on the primary outcome, the GPTS subscale Ideas of Persecution and the between-group difference at treatment cessation. A clinically meaningful group difference was defined as Cohen’s *d* ≥ 0.33, corresponding to a difference of 6.0 or more on the GPTS subscale Ideas of Persecution. We utilized a pooled s.d. of 17.1, obtained from a previous study^25^. To achieve 80% power with a two-sided alpha level of 0.05, the trial required a total of 256 participants, with equal randomization of 128 participants to each of the two intervention arms. All analyses adhered to the ITT principle. Participants who withdrew their informed consent were excluded from analysis.

Statistical analyses were performed using STATA/SE v.18.5, SPSS v.29.0.1.0 (171) and R v.4.5.0. To compare all prespecified outcomes at each follow-up between the two groups, we conducted linear regression models adjusted for stratification variables. All analyses on prespecified outcomes were conducted by the independent trial statistician. Stratification variables were biological sex assigned at birth, study site and dichotomized symptom severity of GPTS subscale Ideas of Persecution of ≥45 or <45 at baseline. The cutoff score of 45 was chosen as it is recommended by the authors of the GPTS as the threshold “to identify severe persecutory ideation and the likely presence of a persecutory delusion”^40^. Baseline measure of each outcome was used for adjustment in all analyses. Sociodemographic characteristics and prespecified outcome measures were balanced at baseline, except for the following, where differences were evaluated as potentially clinically relevant: IBT automatic^28^, where VR-CBTp scored lower than CBTp, and two items from the TALE: items 4 (sudden change in life circumstances) and 8 (physical abuse—familiar perpetrator), where VR-CBTp scores were higher. As these imbalanced variables were not considered as plausible strong prognostic factors for the primary outcome, we followed the recommendation by Van Lancker et al.^71^ not to adjust for them in the primary analyses. However, to keep adherence to the predefined protocol and given their potential prognostic value, we conducted sensitivity analyses with adjustment for baseline imbalances. These analyses were highlighted together with the primary analyses in ʽResultsʼ when they displayed statistically significant differences between groups.

To evaluate the assumptions underlying the linear regression models, residual plots were examined. If they revealed a non-normal distribution, log transformation was applied to test if it could improve the model fit. Further, to account for potential non-normal distributions, a Mann–Whitney *U* nonparametric test was performed.

Missing data were handled by multiple imputations, incorporating variables associated with attrition at treatment cessation into the statistical model. Attrition at treatment cessation was statistically significantly associated with several variables. Participants lost to follow-up were more likely to report a history of sexual abuse before age 16 and distressing events during interaction with mental health services (TALE items 13 and 17). In addition, these participants had statistically significant higher scores on CDSS, lower scores on GSE and WHO-5, and lower conscientiousness scores alongside higher neuroticism scores on the Big-5 personality trait scale. We performed 100 Markov Chain Monte Carlo imputations for each variable using Jeffrey’s uninformative prior. Due to the high proportion of missing data, approaching 40% on certain outcome variables at follow-up, we performed sensitivity analyses based on complete-case-only data. No outcome measures presented missing data <5% (ref. ^72^). For details on percentage missing data, see Supplementary Tables 15–18.

To evaluate the development over the three time points, the interaction between time and intervention was evaluated using linear mixed-model analyses with repeated measurements and an unstructured covariance matrix with participant identification as random effect.

As outlined in the protocol, we performed sensitivity analyses to assess the robustness of our original GPTS scores by comparing it to the R-GPTS scores. Specifically, sensitivity analyses evaluated the primary outcome, Ideas of Persecution, at treatment cessation, and the secondary outcomes, Ideas of Persecution at follow-up and Ideas of Social Self-reference at treatment cessation and follow-up. This approach reflects the updated recommendation to use the R-GPTS as the preferred measure^40^. Results from the sensitivity analyses were compared to the primary analyses to determine the impact of using R-GPTS on outcome interpretations.

Completion of treatment was conservatively defined as attending all 10 sessions. Based on this definition, the completion rates were 75.8% and 81.0% for CBTp and VR-CBTp, respectively. Given that more than 20% of participants did not receive the full intervention, we conducted per-protocol sensitivity analyses to supplement the primary ITT analyses and its sensitivity analyses with adjustment for baseline imbalances. The per-protocol analyses aimed to provide an estimate of the interventions efficacy by including only participants completing all treatment sessions. Descriptive statistics are reported for each randomized group, including baseline values except in the complete-case-only and per-protocol analyses. Binary and categorical variables are presented as counts and percentages, while continuous variables are shown as means with 95% CIs or medians with 25th and 75th percentiles and accompanied by counts. The reported *P* values are two-sided.

The adjusted mean difference between groups should be interpreted consistently across all analyses: a positive value indicates that the CBTp group had a higher value than the VR-CBTp group, while a negative value indicates the opposite.

### Protocol deviations

As detailed in the protocol update^50^, we retained the original primary outcome measure, GPTS subscale Ideas of Persecution, instead of adopting the GPTS subscale Ideas of Social Self-reference, as the ethical committee did not approve the proposed change. We intended to replace Ideas of Persecution with Ideas of Social Self-reference based on clinical observations during baseline assessments making it evident that participants with schizotypal disorders frequently reported lower levels of persecutory ideation, sometimes to the extent that clinically meaningful change was improbable. To better capture the range of distressing experiences across diagnostic categories, the primary outcome was adjusted to focus on ideas of reference. This shift aimed to ensure the measure’s sensitivity and relevance to the study population. This potential change was subsequently found to have no effect, as no statistically significant differences were observed between the groups for either the GPTS subscale Ideas of Social Self-reference or Ideas of Persecution.

As outlined in the protocol, we initially planned to assess participants 3 months postbaseline, implicitly after completing treatment. However, completing treatment within this period proved unrealistic due to various challenges. Given this, we adopted a more pragmatic approach to ensure the study’s objectives were met. Our priority was completing treatment, followed by the assessment. When treatment was delayed, we prioritized the assessment after treatment cessation, with follow-up 6 months later. For participants who discontinued treatment but attended assessments or provided medical records, we adhered to the original 3-month and 9-month postbaseline time points.

Statistical analyses were carried out following the approach recommended by Van Lancker et al.^71^, which suggests that adjusting for baseline imbalances is only beneficial if the variables are prognostic for the outcome. Given the baseline imbalances observed in our study, we determined that none of the variables were plausibly prognostic for our primary outcome. As a result, the primary analysis was conducted without adjusting for these imbalances. However, to evaluate their potential impact, we performed sensitivity analyses on the primary ITT, adjusting for baseline imbalances in adherence to our predefined protocol. These sensitivity analyses have been given a prominent place in the ‘Results’ to remain consistent with the protocol.

We were unable to include patients who were not proficient in reading Danish due to insufficient resources for translating treatment manuals.

The exploratory outcome measures, the SFS and the BCSS, are not listed in the protocol, but prespecified and listed on www.ClinicalTrials.gov at trial registration release on 19 April 2021.

The SFS prosocial activities subscale was excluded due to its sensitivity to COVID-19 restrictions in Denmark (March 2020 to February 2022).

### Reporting summary

Further information on research design is available in the Nature Portfolio Reporting Summary linked to this article.

## Online content

Any methods, additional references, Nature Portfolio reporting summaries, source data, extended data, supplementary information, acknowledgements, peer review information; details of author contributions and competing interests; and statements of data and code availability are available at 10.1038/s41591-025-03880-8.

## Supplementary information

Supplementary Information Supplementary Tables 1–20.

Reporting Summary

## Source data

Source Data Fig. 1 Statistical source data.

## Data Availability

Open access information on the FaceYourFears trial is published on ClinicalTrials.gov (registration: NCT04902066). The final protocol and subsequent update are published in Trials. All deidentified trial data are available through the Danish National Archives (Rigsarkivet), a public data repository, for an unlimited period. Access requests can be submitted via www.rigsarkivet.dk. Due to the Danish Archives Act (Arkivloven), the Danish Archives Executive Order (Arkivbekendtgørelsen), the General Data Protection Regulation (Databeskyttelsesforordningen) and the Danish Data Protection Act (Databeskyttelsesloven), access is restricted. For the first 20 years, data access is subject to prior review by the research group. Access must always be approved by the Danish Data Protection Agency (Datatilsynet), as the data are considered sensitive personal information. After 75 years, the data will be openly accessible without the need for approval. In principle, the research group will grant access to academic or clinical researchers conducting noncommercial, ethically approved research. An initial response to access requests will be provided within 1 month. A Data Access Agreement must be signed before data sharing. Source data are provided with this paper.

## Code availability

The code is available at https://codeberg.org/VIRTU/faceyourfears.
