# Supplementary material for: Virtual reality-based versus standard cognitive behavioral therapy for paranoia in schizophrenia spectrum disorders: a randomized controlled trial
Source: Nat Med. 2025 Aug 13;31(10):3425–39. doi: 10.1038/s41591-025-03880-8 (PMC12532561; doi:10.1038/s41591-025-03880-8)
Supplement: Supplementary file 1 — Supplementary Tables 1–20. [file 41591_2025_3880_MOESM1_ESM.pdf]

nature medicine

Supplementary information https://doi.org/10.1038/s41591-025-03880-8

Virtual reality-based versus standard cognitive behavioral therapy for paranoia in schizophrenia spectrum disorders: a randomized controlled trial

In the format provided by the authors and unedited

Supplementary table 1| The distribution of completed treatment sessions within each group and overall

Number of sessions completed	0	1	2	3	4	5
CBTp	5 (3.9%)	6 (4.7%)	2 (1.6%)	6 (4.7%)	1 (0.8%)	0
VR-CBTp	0	2 (1.6%)	3 (2.4%)	4 (3.2%)	2 (1.6%)	6 (4.8%)
Total	5 (2.0%)	8 (3.1%)	5 (2.0%)	10 (3.9%)	3 (1.2%)	6 (2.4%)
Number of sessions completed	6	7	8	9	10	Total
CBTp	1 (0.8%)	3 (2.3%)	4 (3.1%)	3 (2.3%)	97 (75.8%)	128 (100%)
VR-CBTp	2 (1.6%)	1 (0.8%)	4 (3.2%)	0	102 (81.0%)	126 (100%)
Total	3 (1.2%)	4 (1.6%)	8 (3.1%)	3(1.2%)	199 (78.3%)	254 (100%)

Values are presented as n(n%), indicating number of participants who completed the specific number of treatment sessions and its corresponding percentage.

Supplementary table 2| Days between baseline, treatment cessation and follow-up assessments for both groups, the CBTp group without an extreme outlier and in total

Days between baseline and treatment cessation in each group				
	VR-CBTp mean (95%CI)	CBTp mean (95%CI)	Mean difference (standard error)	95%CI for the mean difference; p value
From baseline to treatment cessation	128 (123-134%)	140 (130-149%)	-11 (5.6)	(-22 to -0.05%); p=0.049*

Days between baseline and follow-up in each group				
	VR-CBTp mean (95%CI)	CBTp mean (95%CI)	Mean differences (standard error)	95%CI for the mean difference; p value
From baseline to follow-up	309 (302-316%)	322 (310-334%)	-13 (6.9)	(-27 to 0.17%); p=0.053

Days between baseline and treatment cessation after excluding one extreme outlier in the CBTp group				
	VR-CBTp mean (95%CI)	CBTp mean (95%CI)	Mean differences (standard error)	95%CI for the mean difference; p value
From baseline to treatment cessation	128 (123-134%)	136 (129-144%)	-8 (4.7)	(-17 to 1%); p=0.10

Days between baseline and treatment cessation in total		
	Total mean (95%CI)	SE
From baseline to treatment cessation	134 (128-139%)	2.8
From baseline to follow-up	315 (308-322%)	3.5

Two-sample t-test was applied to assess the difference in means between the two groups. Values are presented as n(n-n%), indicating the mean value and its 95 % confidence interval and * indicates p value < 0.05.

Supplementary table 3| Additional psychosocial interventions as part of TAU in each group

	VR-CBTp, n (%)	CBTp, n (%)	Total, n (%); p value
Psychoeducation from baseline to treatment cessation	14 (11.1%), n=126	10 (7.8%), n=128	24 (9.5 %); 0.37, n=254
Psychoeducation from treatment cessation to follow-up	9 (7.1%), n=126	6 (4.7%), n=128	15 (5.9%); 0.41, n=254
Social skills training from baseline to treatment cessation	11 (8.7%), n=126	14 (10.9%), n=128	25 (9.8%); 0.55, n=254
Social skills training from treatment cessation to follow-up	12 (9.5%), n=126	10 (7.8%), n=128	22 (8.7%); 0.63, n=254
Individual psychological counseling from baseline to treatment cessation	5 (4.0%), n=126	6 (4.7%), n=128	11 (4.3%); 0.78, n=254
Individual psychological counseling from treatment cessation to follow-up	11 (8.7%), n=126	10 (7.8%), n=128	21 (8.3%); 0.79, n=254
Other psychosocial intervention from baseline to treatment cessation	21 (16.7%), n=126	13 (10.2%), n=128	34 (13.4%); 0.13, n=254
Other psychosocial intervention from treatment cessation to follow-up	25 (19.8%), n=126	15 (10.7%), n=128	40 (15.8%); 0.08, n=254

Chi-square test assessed the association between binary categorial variables. Values are presented as n(n%), indicating the frequency and its corresponding percentage of the total and n= indicates number of observations. Time points: Treatment cessation, mean = 4.5 months (95%CI 4.3-4.6) after baseline; Follow-up, mean = 10.5 months (95%CI 10.3-10.7) after baseline.

Supplementary table 4| Mann–Whitney U test-results to test for between group differences

Analysis	Outcome (all at treatment cessation)	Mann–Whitney U p-value
Intention-to-treat	GPTS - Ideas of Persecution	0.70
Intention-to-treat	SBQ sub-score avoidance	0.89
Intention-to-treat	CDSS total	0.54
Intention-to-treat	SIDAS	0.10
Intention-to-treat	BCSS – negative others	0.04*
Intention-to-treat	R-GPTS - Ideas of Persecution	0.76
Complete-case-only	GPTS - Ideas of Persecution	0.70
Complete-case-only	SBQ sub-score avoidance	0.89
Complete-case-only	CDSS total	0.54
Complete-case-only	SIDAS	0.10
Complete-case-only	BCSS – negative others	0.04*
Complete-case-only	R-GPTS - Ideas of Persecution	0.76
Per-protocol	GPTS - Ideas of Persecution	0.66
Per-protocol	SBQ sub-score avoidance	0.96
Per-protocol	CDSS total	0.81
Per-protocol	SIDAS	0.13
Per-protocol	BCSS – negative others	0.18
Per-protocol	R-GPTS - Ideas of Persecution	0.73

Mann-Whitney U tests are conducted to test for statistical significant differences between-groups on outcome measures not adjusted for baseline imbalances, which have been log-transformed to improve model fit. * indicates p value < 0.05.

Supplementary table 5| Intention-to-treat sensitivity analyses: Between-group adjusted mean difference adjusted for baseline imbalances: A sensitivity analysis on the primary outcome

	VR-CBTp mean (95%CI)	CBTp mean (95%CI)	Adjusted mean difference (standard error)	95%CI for adjusted mean difference; p value
GPTS – Ideas of Persecution				
Baseline	41.1 (38.6-43.7%) n=126	41.1 (38.2-43.9%) n=128		
Treatment cessation	29.8 (27.8-31.9%)	30.7 (28.6-32.8%)	1.01* (0.07)	(0.87 to 1.16%)*; 0.94

Between-group adjusted mean difference after adjusting for biological sex assigned at birth, study site and dichotomised symptom severity of GPTS subscale *Ideas of Persecution* (≥45 or <45 at baseline). The analysis is a linear regression model based on the intention-to-treat principle and handled with multiple imputations. Analysis is adjusted for baseline measurement of GPTS, ideas of Persecution. Analysis is conducted with adjustment for baseline imbalances. *: Due to the non-normal distribution of the residual plots, a log transformation was applied, which improved the model fit; the reported result is therefore an exponentiated, back-transformed value. Values are presented as n(n-n%) indicating the mean value and its 95 % confidence interval, n= indicates number of observations at baseline. Time points: Treatment cessation, mean = 4.5 months (95%CI 4.3-4.6) after baseline; Follow-up, mean = 10.5 months (95%CI 10.3-10.7) after baseline. GPTS: Green Paranoid Thought Scale.

Supplementary table 6| Intention-to-treat sensitivity analyses: Between-group adjusted mean difference adjusted for baseline imbalances: Sensitivity analyses on the secondary outcomes

	VR-CBTp mean (95%CI)	CBTp mean (95%CI)	Adjusted mean difference (standard error)	95%CI for adjusted mean difference; p value
GPTS – Ideas of Persecution				
Follow-up	29.2 (26.7-31.6%)	30.5 (27.7-33.2%)	1.29 (1.94)	(-2.55 to 5.13%); 0.51
GPTS – Ideas of Social Self-Reference				
Baseline	44.0 (41.7-46.3%) n=126	45.2 (43.0-47.4%) n=128		
Treatment cessation	33.5 (31.7-35.4%)	34.1 (32.1-36.1%)	0.57 (1.38)	(-2.16 to 3.30%); 0.68
Follow-up	32.8 (30.6-35.0%)	33.6 (31.3-36.0%)	0.85 (1.71)	(-2.52 to 4.23%); 0.62
PSP total				
Baseline	42.7 (40.5-44.9%) n=126	45.3 (43.0-47.5%) n=128		
Treatment cessation	48.7 (47.4-50.0%)	49.1 (47.8-50.5%)	0.44 (0.97)	(-1.48 to 2.36%); 0.65
Follow-up	50.5 (48.5-52.5%)	52.3 (49.9-54.7%)	1.74 (1.64)	(-1.50 to 4.98%); 0.29
SBQ total				
Baseline	55.1 (51.0-59.2%) n=126	54.2 (50.0-58.4%) n=128		
Treatment cessation	36.7 (34.0-39.3%)	38.3 (35.4-41.2%)	1.63 (2.06)	(-2.43 to 5.70%); 0.43
Follow-up	34.9 (31.3-38.4%)	33.8 (29.4-38.1%)	-1.09 (2.86)	(-6.74 to 4.55%); 0.70
SBQ sub score avoidance				
Baseline	38.9 (37.1-40.8%) n=126	38.3 (36.4-40.2%) n=128		
Treatment cessation	9.4 (8.3-10.6%)	9.1 (7.8-10.5%)	0.92* (0.05)	(0.50 to 1.69%)*; 0.79
Follow-up	8.9 (7.5-10.4%)	8.1 (6.3-9.9%)	-0.81 (1.17)	(-3.12 to 1.49%); 0.49
ERT - latency overall				
Baseline	3219 (2833-3604%) n=124	2882 (2568-3196%) n=126		
Treatment cessation	2310 (2105-2514%)	2509 (2290-2728%)	199.7 (153.8)	(-103.8 to 503.2%); 0.20
Follow-up	2350 (2079-2620%)	1984 (1756-2213%)	-365.4 (181.3)	(-724.1 to -6.7%); 0.046**
ERT - latency happiness				
Baseline	1170 (1072-1267%) n=124	1077 (1027-1127%) n=126		

Treatment cessation	1010 (945-1074%)	1008 (942-1074%)	-1.60 (47.4)	(-95.19 to 91.99%); 0.97
Follow-up	1053 (974-1132%)	1005 (922-1088%)	-48.4 (60.6)	(-168.3 to 71.5%); 0.43
ERT - latency sadness				
Baseline	2116 (1870-2363%) n=124	1978 (1832-2123%) n=126		
Treatment cessation	1748 (1636-1860%)	1681 (1568-1794%)	-66.6 (81.2)	(-226.9 to 93.6%); 0.41
Follow-up	18001 (1611-1991%)	1587 (1403-1771%)	-213.9 (137.7)	(-486.3 to 58.6%); 0.12
ERT - latency fear				
Baseline	2120 (1880-2360%) n=124	2104 (1898-2309%) n=126		
Treatment cessation	1861 (1722-1999%)	1836 (1682-1991%)	-24.2 (107.4)	(-236.1 to 187.8%); 0.82
Follow-up	1691 (1543-1838%)	1638 (1488-1788%)	-53.2 (109.7)	(-270.3 to 163.9%); 0.63
ERT - latency anger				
Baseline	1452 (1312-1592%) n=124	1355 (1266-1443%) n=126		
Treatment cessation	1311 (1224-1397%)	1220 (1128-1311%)	-90.8 (64.6)	(-218.3 to 36.7%); 0.16
Follow-up	1330 (1219-1441%)	1215 (1097-1332%)	-115.5 (84.0)	(-281.6 to 50.6%); 0.17
ERT - latency surprise				
Baseline	1336.1 (1203.0-1449.2%) n=124	1222.8 (1140.2-1305.3%) n=126		
Treatment cessation	1128.8 (1050.5-1207.0%)	1124.1 (1044.6-1203.6%)	-4.64 (58.37)	(-119.82 to 110.54%); 0.94
Follow-up	1125.6 (1051.5-1199.6%)	1029.0 (955.0-1109.0%)	-96.58 (54.79)	(-204.86 to 11.69%); 0.08
ERT - latency disgust				
Baseline	2145 (1939-2351%) n=124	1960 (1804-2116%) n=126		
Treatment cessation	1663 (1547-1779%)	1768 (1639-1896%)	105.1 (91.0)	(-74.6 to 284.7%); 0.25
Follow-up	1638 (1504-1772%)	1569 (1438-1700%)	-68.9 (95.8)	(-258.3 to 120.4%); 0.47
ERT - accuracy overall				
Baseline	56.6 (54.8-58.3%) n=124	56.1 (54.5-57.6%) n=126		
Treatment cessation	57.8 (56.4-59.3%)	58.1 (56.6-59.5%)	0.23 (1.03)	(-1.79 to 2.26%); 0.82
Follow-up	57.4 (56.0-58.9%)	58.7 (57.0-60.3%)	1.23 (1.10)	(-0.93 to 3.40%); 0.26

ERT - accuracy happiness				
Baseline	11.4 (11.0-11.7%) n=124	11.5 (11.2-11.9%) n=126		
Treatment cessation	11.7 (11.4-12.1%)	11.5 (11.1-11.8%)	-0.25 (0.26)	(-0.77 to 0.26%); 0.33
Follow-up	11.7 (11.3-12.1%)	11.5 (11.0-12.0%)	-0.19 (0.34)	(-0.86 to 0.48%); 0.58
ERT - accuracy sadness				
Baseline	10.1 (9.5-10.6%) n=124	9.4 (8.8-9.9%) n=126		
Treatment cessation	9.4 (8.9-9.9%)	10.1 (9.6-10.6%)	0.66 (0.36)	(-0.06 to 1.37%); 0.07
Follow-up	9.6 (9.0-10.1%)	10.6 (10.0-11.1%)	1.01 (0.40)	(0.21 to 1.81%); 0.01**
ERT - accuracy fear				
Baseline	6.3 (5.8-6.9%) n=124	6.1 (5.6-6.7%) n=126		
Treatment cessation	6.6 (6.0-7.1%)	7.2 (6.6-7.7%)	0.59 (0.42)	(-0.23 to 1.42%); 0.16
Follow-up	6.4 (5.7-7.0%)	7.0 (6.3-7.7%)	0.62 (0.49)	(-0.35 to 1.58%); 0.21
ERT - accuracy anger				
Baseline	7.9 (7.4-8.4%) n=124	7.9 (7.5-8.3%) n=126		
Treatment cessation	8.4 (8.0-8.8%)	8.4 (8.0-8.8%)	-0.01 (0.27)	(-0.55 to 0.52%); 0.96
Follow-up	8.4 (7.9-8.9%)	8.8 (8.2-9.3%)	0.38 (0.38)	(-0.36 to 1.13%); 0.31
ERT - accuracy surprise				
Baseline	11.4 (11.1-11.8%) n=124	11.4 (11.1-11.7%) n=126		
Treatment cessation	11.6 (11.3-12.0%)	11.2 (10.8-11.5%)	-0.47 (0.27)	(-1.00 to 0.06%); 0.08
Follow-up	11.4 (11.0-11.8%)	11.1 (10.7-11.6%)	-0.31 (0.33)	(-0.96 to 0.33%); 0.34
ERT - accuracy disgust				
Baseline	9.4 (8.8-10.1%) n=124	9.8 (9.1-10.4%) n=126		
Treatment cessation	10.0 (9.5-10.6%)	9.8 (9.2-10.4%)	-0.20 (0.42)	(-1.02 to 0.62%); 0.63
Treatment cessation	57.8 (56.4-59.3%)	58.1 (56.6-59.5%)	0.23 (1.03)	(-1.79 to 2.26%); 0.82
Follow-up	57.4 (56.0-58.9%)	58.7 (57.0-60.3%)	1.23 (1.10)	(-0.93 to 3.40%); 0.26
ERT - accuracy happiness				
Baseline	11.4 (11.0-11.7%) n=124	11.5 (11.2-11.9%) n=126		

Treatment cessation	11.7 (11.4-12.1%)	11.5 (11.1-11.8%)	-0.25 (0.26)	(-0.77 to 0.26%); 0.33
Follow-up	11.7 (11.3-12.1%)	11.5 (11.0-12.0%)	-0.19 (0.34)	(-0.86 to 0.48%); 0.58
ERT - accuracy sadness				
Baseline	10.1 (9.5-10.6%) n=124	9.4 (8.8-9.9%) n=126		
Treatment cessation	9.4 (8.9-9.9%)	10.1 (9.6-10.6%)	0.66 (0.36)	(-0.06 to 1.37%); 0.07
Follow-up	9.6 (9.0-10.1%)	10.6 (10.0-11.1%)	1.01 (0.40)	(0.21 to 1.81%); 0.01**
ERT - accuracy fear				
Baseline	6.3 (5.8-6.9%) n=124	6.1 (5.6-6.7%) n=126		
Treatment cessation	6.6 (6.0-7.1%)	7.2 (6.6-7.7%)	0.59 (0.42)	(-0.23 to 1.42%); 0.16
Follow-up	6.4 (5.7-7.0%)	7.0 (6.3-7.7%)	0.62 (0.49)	(-0.35 to 1.58%); 0.21
ERT - accuracy anger				
Baseline	7.9 (7.4-8.4%) n=124	7.9 (7.5-8.3%) n=126		
Treatment cessation	8.4 (8.0-8.8%)	8.4 (8.0-8.8%)	-0.01 (0.27)	(-0.55 to 0.52%); 0.96
Follow-up	8.4 (7.9-8.9%)	8.8 (8.2-9.3%)	0.38 (0.38)	(-0.36 to 1.13%); 0.31
ERT - accuracy surprise				
Baseline	11.4 (11.1-11.8%) n=124	11.4 (11.1-11.7%) n=126		
Treatment cessation	11.6 (11.3-12.0%)	11.2 (10.8-11.5%)	-0.47 (0.27)	(-1.00 to 0.06%); 0.08
Follow-up	11.4 (11.0-11.8%)	11.1 (10.7-11.6%)	-0.31 (0.33)	(-0.96 to 0.33%); 0.34
ERT - accuracy disgust				
Baseline	9.4 (8.8-10.1%) n=124	9.8 (9.1-10.4%) n=126		
Treatment cessation	10.0 (9.5-10.6%)	9.8 (9.2-10.4%)	-0.20 (0.42)	(-1.02 to 0.62%); 0.63
Follow-up	10.0 (9.5-10.5%)	9.7 (9.1-10.3%)	-0.30 (0.40)	(-1.10 to 0.49%); 0.45
SIAS				
Baseline	46.8 (44.3-49.4%) n=126	47.8 (45.2-50.5%) n=127		
Treatment cessation	41.8 (39.8-43.9%)	42.1 (39.8-44.4%)	0.25 (1.60)	(-2.90 to 3.40%); 0.88
Follow-up	39.7 (37.1-42.3%)	38.3 (35.4-41.2%)	-1.37 (1.96)	(-5.24 to 2.51%); 0.49

Between-group adjusted mean difference after adjusting for biological sex assigned at birth, study site and dichotomised symptom severity of GPTS subscale *Ideas of Persecution* (≥45 or <45 at baseline). All analyses are linear regression models based on the intention-to-treat principle and handled with multiple imputations. All analyses are adjusted for baseline measurement of each outcome. All analyses are conducted with adjustment for baseline imbalances. Values

are presented as n(n-n%), indicating the mean value and its 95 % confidence interval, n= indicates number of observations at baseline. * indicates that a log transformation was applied, and the reported result is therefore an exponentiated, back-transformed value. ** indicates a p value < 0.05. Time points: Treatment cessation, mean = 4.5 months (95%CI 4.3-4.6) after baseline; Follow-up, mean = 10.5 months (95%CI 10.3-10.7) after baseline. GPTS: Green Paranoid Thought Scale. GPTS: Green Paranoid Thought Scale. PSP: Personal and Social Performance scale. SBQ: Safety Behavior Questionnaire. ERT: Emotion Recognition Task. SIAS: Social Interaction Anxiety Scale.

Supplementary table 7| Intention-to-treat sensitivity analyses: Between-group adjusted mean difference adjusted for baseline imbalances: Sensitivity analyses on the exploratory outcomes

	VR-CBTp mean (95%CI)	CBTp mean (95%CI)	Adjusted mean difference (standard error)	95%CI for adjusted mean difference; p value
SAPS Global				
Baseline	7.8 (7.4-8.3%) n=126	7.7 (7.1-8.2%) n=128		
Treatment cessation	6.4 (6.0-7.8%)	6.4 (6.0-6.8%)	-0.01 (0.27)	(-0.54 to 0.53%); 0.98
Follow-up	5.8 (5.2-6.3%)	5.8 (5.2-6.3%)	0.03 (0.39)	(-0.74 to 0.80%); 0.94
SAPS Composite				
Baseline	22.9 (20.6-25.0%) n=126	21.8 (19.7-23.9%) n=128		
Treatment cessation	16.6 (15.5-17.8%)	17.3 (16.0-18.6%)	0.66 (0.90)	(-1.12 to 2.44%); 0.47
Follow-up	14.5 (13.1-15.8%)	15.1 (13.6-16.7%)	0.70 (1.11)	(-1.49 to 2.89%); 0.53
BNSS total				
Baseline	23.4 (21.7-25.2%) n=126	23.4 (21.4-25.3%) n=126		
Treatment cessation	20.5 (19.4-21.7%)	21.2 (20.0-22.4%)	0.66 (0.85)	(-1.02 to 2.33%); 0.44
Follow-up	19.7 (18.2-21.3%)	18.3 (16.5-20.1%)	-1.42 (1.25)	(-3.88 to 1.05%); 0.26
CDSS total				
Baseline	6.1 (5.4-6.8%) n=124	5.7 (5.1-6.3%) n=126		
Treatment cessation	4.5 (3.9-5.0%)	4.9 (4.2-5.5%)	0.82(0.24)	(0.51 to 1.31%)**; 0.40
Follow-up	4.8 (4.1-5.5%)	4.8 (3.9-5.6%)	-0.01 (0.59)	(-1.17 to 1.16%); 0.99
COGDIS total				
Baseline	18.1 (16.3-19.8%) n=126	17.7 (16.0-19.3%) n=127		
Treatment cessation	16.2 (14.8-17.6%)	17.9 (16.4-19.5%)	1.72 (1.07)	(-0.39 to 3.84%); 0.11
Follow-up	16.2 (14.3-18.0%)	16.4 (14.3-18.5%)	0.21 (1.47)	(-2.70 to 3.12%); 0.89
Trustworthiness task				
Baseline	-0.22 (-0.33 to -0.13%) n=126	-0.30 (-0.42 to -0.18%) n=128		
Treatment cessation	-0.18 (-0.28 to -0.07%)	-0.10 (-0.21 to 0.02%)	0.08 (0.08)	(-0.08 to 0.23%); 0.32
Follow-up	-0.13 (-0.24 to -0.01%)	-1.11 (-0.25 to 0.02%)	0.02 (0.09)	(-0.17 to 0.20%); 0.86
SSPA (SCOPE variables)				
Baseline	4.09 (3.99-4.20%)	4.14 (4.05-4.24%)		

	n=125	n=126		
Treatment cessation	4.38 (4.30-4.46%)	4.30 (4.20-4.40%)	-0.08 (0.07)	(-0.21 to 0.5%); 0.24
Follow-up	4.42 (4.34-4.49%)	4.39 (4.29-4.50%)	-0.02 (0.07)	(-0.15 to 0.11%); 0.74
IBT total				
Baseline	0.53 (0.51-0.56%) n=124	0.57 (0.55-0.60%) n=127		
Treatment cessation	0.52 (0.50-0.54%)	0.54 (0.51-0.56%)	0.02 (0.02)	(-0.01 to 0.05%); 0.23
Follow-up	0.52 (0.49-0.54%)	0.53 (0.51-0.56%)	0.01 (0.02)	(-0.03 to 0.05%); 0.47
IBT Automatic				
Baseline	0.58 (0.54-0.63%) n=124	0.65 (0.61-0.70%) n=127		
Treatment cessation	0.56 (0.52-0.60%)	0.61 (0.56-0.65%)	0.05 (0.03)	(-0.01 to 0.11%); 0.37
Follow-up	0.57 (0.52-0.61%)	0.58 (0.52-0.63%)	0.01 (0.04)	(-0.06 to 0.08%); 0.85
IBT Control				
Baseline	0.38 (0.34-0.43%) n=124	0.38 (0.34-0.42%) n=127		
Treatment cessation	0.42 (0.37-0.46%)	0.40 (0.35-0.46%)	-0.01 (0.03)	(-0.08 to 0.05%); 0.68
Follow-up	0.39 (0.35-0.44%)	0.38 (0.33-0.42%)	-0.02 (0.03)	(-0.08 to 0.05%); 0.60
SIDAS				
Baseline	5 (1-15)* n=126	7 (0-14)* n=127		
Treatment cessation	6.4 (5.1-7.7%)	8.3 (6.9-9.8%)	1.51** (0.36)	(0.73 to 3.11%)**; 0.26
Follow-up	6.8 (5.2-8.4%)	8.5 (6.7-10.3%)	1.72 (1.25)	(-0.74 to 4.18%); 0.17
BCSS – negative self				
Baseline	10.1 (9.2-11.0%) n=126	10.2 (9.4-11.1%) n=128		
Treatment cessation	7.9 (7.2-8.5%)	8.5 (7.8-9.3%)	0.66 (0.52)	(-0.37 to 1.69%); 0.21
Follow-up	7.2 (6.4-8.0%)	8.0 (7.1-8.9%)	0.77 (0.62)	(-0.45 to 1.99%); 0.21
BCSS – negative others				
Baseline	7.5 (6.7-8.4%) n=126	8.7 (7.8-9.6%) n=128		
Treatment cessation	5.7 (5.0-6.5%)	6.6 (5.8-7.4%)	1.16** (0.24)	(0.72 to 1.87%)**; 0.54
Follow-up	6.3 (5.5-7.1%)	6.0 (5.1-6.8%)	-0.33 (0.60)	(-1.52 to 0.86%); 0.51
BCSS – positive self				
Baseline	8.1 (7.1-9.0%) n=126	7.5 (6.7-8.3%) n=128		
Treatment cessation	8.7 (8.0-9.4%)	8.8 (8.1-9.6%)	0.19 (0.52)	(-0.85 to 1.22%); 0.72

Follow-up	9.2 (8.3-10.0%)	9.9 (8.9-10.8%)	0.70 (0.67)	(-0.63 to 2.03%); 0.30
BCSS – positive others				
Baseline	9.1 (8.4-9.9%) n=126	8.5 (7.8-9.1%) n=128		
Treatment cessation	10.1 (9.3-10.8%)	9.9 (9.1-10.7%)	-0.14 (0.54)	(-1.21 to 0.93%); 0.80
Follow-up	10.3 (9.4-11.2%)	10.3 (9.3-11.3%)	-0.02 (0.70)	(-1.41 to 1.37%); 0.98
DACOBS - Jumping to conclusion				
Baseline	22.3 (21.3-23.2%) n=126	22.1 (21.2-23.0%) n=128		
Treatment cessation	21.9 (21.1-22.7%)	21.4 (20.5-22.3%)	-0.49 (0.64)	(-1.74 to 0.76%); 0.44
Follow-up	21.9 (21.0-22.8%)	21.7 (20.7 – 22.7%)	-0.19 (0.71)	(-1.60 to 1.21%); 0.79
DACOBS - Belief inflexibility				
Baseline	21.0 (20.1-22.0%) n=126	21.2 (20.3-22.2%) n=128		
Treatment cessation	20.6 (19.8-21.3%)	19.8 (19.0-20.6%)	-0.77 (0.59)	(-1.93 to 0.39%); 0.19
Follow-up	19.9 (19.1-20.8%)	19.6 (18.7-20.5%)	-0.32 (0.564)	(-1.58 to 0.94%); 0.62
DACOBS - Attention for threat				
Baseline	30.9 (30.0-31.8%) n=126	30.3 (29.4-31.3%) n=128		
Treatment cessation	27.1 (26.2-28.0%)	27.7 (26.7-28.6%)	0.61 (0.68)	(-1.74 to 1.96%); 0.38
Follow-up	26.3 (25.1-27.5%)	26.7 (25.4-28.1%)	0.45 (0.91)	(-1.35 to 2.25%); 0.62
DACOBS - External attribution				
Baseline	21.9 (20.8-22.9%) n=126	22.3 (21.3-23.4%) n=128		
Treatment cessation	20.2 (19.4-21.0%)	20.7 (19.9-21.5%)	0.47 (0.58)	(-0.68 to 1.61%); 0.42
Follow-up	20.0 (19.1-21.0%)	19.3 (18.4-20.3%)	-0.70 (0.68)	(-2.04 to 0.65%); 0.31
DACOBS - Social cognitive problems				
Baseline	29.4 (28.4-30.4%) n=126	29.7 (30.4%) n=128		
Treatment cessation	26.2 (25.3-27.1%)	26.5 (25.5-27.5%)	0.27 (0.70)	(-1.11 to 1.66%); 0.70
Follow-up	25.6 (24.4-26.7%)	26.2 (25.0-27.5%)	0.68 (0.87)	(-1.04 to 2.40%); 0.44
DACOBS - Subjective cognitive problems				
Baseline	28.4 (27.3-29.6%) n=126	29.4 (28.4-30.4%) n=128		

Treatment cessation	27.0 (26.1-28.0%)	27.1 (26.1-28.2%)	0.11 (0.74)	(-1.35 to 1.57%); 0.88
Follow-up	26.5 (25.4-27.7%)	26.9 (25.6-28.2%)	0.37 (0.91)	(-1.43 to 2.17%); 0.68
DACOBS - Safety behavior				
Baseline	23.0 (21.8-24.3%) n=126	22.7 (21.5-24.0%) n=128		
Treatment cessation	20.3 (19.3-21.4%)	20.5 (19.4-21.7%)	0.24 (0.79)	(-1.32 to 1.80%); 0.76
Follow-up	19.5 (18.3-20.6%)	18.2 (17.0-19.5%)	-1.22 (0.90)	(-3.00 to 0.56%); 0.18
SFS – Social engagement/withdrawal				
Baseline	94.9 (93.3-96.5%) n=126	95.5 (93.8-97.2%) n=126		
Treatment cessation	97.6 (96.3-99.0%)	96.7 (95.3-98.2%)	-0.93 (1.02)	(-2.94 to 1.08%); 0.37
Follow-up	97.9 (96.1-99.7%)	99.5 (97.5-101.4%)	1.53 (1.36)	(-1.16 to 4.23%); 0.26
SFS – Interpersonal behavior				
Baseline	109.3 (106.5-112.1%) n=126	111.0 (108.1-113.9%) n=127		
Treatment cessation	113.3 (111.0-115.6%)	112.5 (110.1-114.9%)	-0.79 (1.71)	(-4.15 to 2.58%); 0.65
Follow-up	113.6 (110.8-116.4%)	115.3 (112.1-118.5%)	1.7 (2.23)	(-2.71 to 6.10%); 0.45
SFS - Recreation				
Baseline	107.3 (104.7-110.0%) n=124	104.3 (101.8-106.8%) n=127		
Treatment cessation	107.5 (105.7-109.3%)	107.8 (105.8-109.8%)	0.27 (1.41)	(-2.51 to 3.05%); 0.85
Follow-up	107.3 (104.9-109.6%)	110.4 (107.6-113.2%)	3.13 (1.88)	(-0.58 to 6.85%); 0.10
SFS – Independence-competence				
Baseline	99.5 (97.5-101.5%) n=123	97.9 (96.0-99.8%) n=127		
Treatment cessation	103.4 (101.9-105.0%)	102.1 (100.4-103.8%)	-1.33 (1.19)	(-3.69 to 1.02%); 0.26
Follow-up	104.0 (101.9-106.0%)	104.2 (101.9%)	0.23 (1.57)	(-2.86 to 3.32%); 0.89
SFS – Independence-performance				
Baseline	97.0 (95.0-99.0%) n=126	96.8 (94.8-98.7%) n=127		
Treatment cessation	99.9 (98.5-101.3%)	99.8 (98.2-101.3%)	-0.23 (1.09)	(-2.28 to 2.02%); 0.91
Follow-up	101.2 (99.5-103.0%)	101.6 (99.6-103.6%)	0.34 (1.41)	(-2.43 to 3.12%); 0.81
SFS – Employment-occupation				
Baseline	101.6 (99.1-104.0%)	104.7 (102.4-107.0%)		

	n=120	n=126		
Treatment cessation	104.1 (102.3-105.8%)	104.3 (102.4-106.2%)	0.25 (1.34)	(-2.40 to 2.89%); 0.86
Follow-up	104.6 (102.4-106.8%)	105.1 (102.5-107.7%)	0.49 (1.79)	(-3.05 to 4.02%); 0.79
GSE				
Baseline	21.1 (20.0-22.2%) n=126	21.0 (20.0-21.9%) n=127		
Treatment cessation	24.3 (23.4-25.2%)	23.4 (22.4-24.3%)	-0.91 (0.68)	(-2.25 to 0.43%); 0.18
Follow-up	24.3 (23.2-25.3%)	24.8 (23.6-26.0%)	0.53 (0.81)	(-1.07 to 2.13%); 0.52
EQ-5D-5L				
Baseline	0.50 (0.45-0.55%) n=126	0.51 (0.46-0.56%) N=124		
Treatment cessation	0.61 (0.57-0.65%)	0.58 (0.53-0.63%)	-0.03 (0.03)	(-0.09 to 0.03%); 0.36
Follow-up	0.64 (0.59-0.68%)	0.65 (0.60-0.68%)	0.01 (0.03)	(-0.06 to 0.08%); 0.81
EQ5 VAS				
Baseline	53.8 (49.9-57.7%) n=121	50.5 (46.9-54.1%) n=124		
Treatment cessation	59.3 (55.5-63.0%)	56.3 (52.1-60.4%)	-3.0 (2.9)	(-8.7 to 2.7%); 0.30
Follow-up	60.7 (56.3-65.0%)	59.4 (54.7-64.1%)	-1.3 (3.3)	(-7.8 to 5.2%); 0.40
WHO 5				
Baseline	33.3 (30.2-36.4%) n=126	30.6 (27.6-33.5%) n=127		
Treatment cessation	39.5 (36.2-42.8%)	41.4 (37.7-45.1%)	1.88 (2.58)	(-3.20 to 6.97%); 0.47
Follow-up	42.8 (38.8 to 46.9%)	44.6 (39.8-49.4%)	1.76 (3.30)	(-4.77 to 8.29%); 0.59
GPTS-R – Ideas of Persecution				
Baseline	15.1 (13.5-16.6%) n=126	14.9 (13.2-16.6%) n=128		
Treatment cessation	8.4 (7.1-9.6%)	8.6 (7.2-10.0%)	0.87** (0.29)	(0.49 to 1.56%)**; 0.65
Follow-up	7.9 (6.4-9.4%)	8.4 (6.7-10.1%)	0.53 (1.16)	(-1.75 to 2.82%); 0.65
GPTS-R – Ideas of Social Self-Reference				
Baseline	13.3 (12.1-14.4%) n=126	14.4 (13.2-15.6%) n=128		
Treatment cessation	8.8 (7.9-9.7%)	8.8 (7.8-9.8%)	0.03 (0.70)	(-1.34 to 1.40%); 0.97
Follow-up	8.3 (7.5-9.4%)	8.8 (7.6-10.0%)	0.48 (0.83)	(-1.17 to 2.13%); 0.56
GPTS total				
Baseline	85.2 (80.7-89.6%) n=126	86.3 (81.3-91.0%) n=128		

Treatment cessation	63.5 (59.9-67.0%)	64.7 (60.9-68.5%)	1.25 (2.68)	(-4.03 to 6.53%); 0.64
Follow-up	61.9 (57.4-66.3%)	64.3 (59.3-69.2%)	2.40 (3.48)	(-4.47 to 9.26%); 0.49
GPTS-R total				
Baseline	28.3 (25.9-30.8%) n=126	29.3 (26.6-31.9%) n=128		
Treatment cessation	17.2 (15.3-19.1%)	17.4 (15.4-19.4%)	0.26 (1.43)	(-2.57 to 3.08%); 0.86
Follow-up	16.1 (13.9-18.4%)	17.3 (14.8-19.8%)	1.14 (1.71)	(-2.24 to 4.51%); 0.51
CSQ				
Treatment cessation	26.8 (26.0-27.5%)	26.2 (25.4-27.0%)	-0.58 (0.56)	(-1.68 to 0.52%); 0.30

Between-group adjusted mean difference after adjusting for biological sex assigned at birth, study site and dichotomised symptom severity of GPTS subscale *Ideas of Persecution* (≥45 or <45 at baseline). All analyses are conducted with adjustment for baseline imbalances. All analyses are linear regression models based on the intention-to-treat principle and handled with multiple imputations. All analyses are adjusted for baseline measurement of each outcome, except for the CSQ that was not administered at baseline. For CSQ, a linear regression model adjusted for biological sex assigned at birth, study site and dichotomised symptom severity of GPTS subscale *Ideas of Persecution* (≥45 or <45 at baseline) was used and the number of observations in the analysis were 212. Values are presented as n(n-n%), indicating the mean value and its 95 % confidence interval, n= indicates number of observations at baseline. * indicates that the numbers presented is the median and its 25th and 75th percentile in a non-normal distributed sample. ** indicates that a log transformation was applied, and the reported result is therefore an exponentiated, back-transformed value. Time points: Treatment cessation, mean = 4.5 months (95%CI 4.3-4.6) after baseline; Follow-up, mean = 10.5 months (95%CI 10.3-10.7) after baseline. SAPS: Scale for the Assessment of Positive Symptoms. BNSS: Brief Negative Symptoms Scale. CDSS: Calgary Depression Symptom Scale. COGDIS: Cognitive Disturbances scale. SSPA: Social Skills Performance Assessment. IBT: Intentionality Bias Task. SIDAS: Suicidal Ideation Attributes Scale. BCSS: Brief Core Schema Scale. DACOBS: Davos Assessment of Cognitive Biases Scale. SFS: Social Functioning Scale. GSE: General Self-Efficacy scale. EQ-5D-5L: 5-Level EuroQol 5 Dimensions version. EQ5-VAS: EuroQol Visual Analogue Scale. WHO 5: World Health Organization-Five well-being index. GPTS-R: Revised Green Paranoid Thought Scale. CSQ: Client Satisfaction Questionnaire.

Supplementary table 8| Complete-case-only sensitivity analysis: Between-group adjusted mean difference without adjustment for baseline imbalances: Sensitivity analysis on the primary outcome

	VR-CBTp mean (95%CI)	CBTp mean (95%CI)	Adjusted mean difference (standard error)	95 % CI for adjusted mean difference; p value	Number of observations, % missing data
GPTS – Ideas of Persecution					
Treatment cessation	29.3 (27.4-21.2%)	30.7 (28.7-32.8%)	1.04 (0.04)	(0.95 to 1.13%)*; 0.39	222, 12,6%

Complete-data-only with numbers of observation and the percentage of missing data. Between-group adjusted mean difference after adjusting for biological sex assigned at birth, study site and dichotomised symptom severity of GPTS subscale *Ideas of Persecution* (≥45 or <45 at baseline). A linear regression model is used for the analysis. Analysis is adjusted for baseline measurement of GPTS, *Ideas of Persecution*. The analysis is conducted without adjustment for baseline imbalances. *: Due to the non-normal distribution of the residual plots, a log transformation was applied, which improved the model fit; the reported result is therefore an exponentiated, back-transformed value. Values are presented as n(n-n%) indicating the mean value and its 95 % confidence. Time points: Treatment cessation, mean = 4.5 months (95%CI 4.3-4.6) after baseline; Follow-up, mean = 10.5 months (95%CI 10.3-10.7) after baseline. GPTS: Green Paranoid Thought Scale.

Supplementary table 9| Complete-case-only sensitivity analyses: Between-group adjusted mean difference without adjustment for baseline imbalances: Sensitivity analyses on the secondary outcomes

	VR-CBTp mean (95%CI)	CBTp mean (95%CI)	Adjusted mean difference (standard error)	95 % CI for the adjusted mean difference; p value	Number of observations, % missing data
GPTS – Ideas of Persecution					
Follow-up	28.9 (26.6-31.3%)	29.5 (27.0-32.0%)	0.55 (1.74)	(-2.89 to 3.99%); 0.75	200, 21.3 %
GPTS – Ideas of Social Self-Reference					
Treatment cessation	33.1 (31.3-34.9%)	33.7 (31.8-35.5%)	0.59 (1.31)	(-2.00 to 3.18%); 0.65	222, 12.6%
Follow-up	32.1 (29.9-34.3%)	32.9 (30.6-35.2%)	0.74 (1.61)	(-2.44 to 3.91%); 0.65	200, 21.3%
PSP total					
Treatment cessation	49.3 (48.1-50.6%)	49.0 (47.7-50.3%)	-0.33 (0.92)	(-2.15 to 1.50%); 0.73	219, 13.8%
Follow-up	51.3 (49.3-53.2%)	51.5 (49.4-53.7%)	0.27 (1.48)	(-2.66 to 3.20%); 0.86	181, 28.7%
SBQ total					
Treatment cessation	36.8 (34.3-39.3%)	39.6 (37.0-42.3%)	2.84 (1.87)	(-0.84 to 6.53%); 0.13	215, 15.4%
Follow-up	34.6 (31.0-38.2%)	33.6 (29.6-37.5%)	-1.05 (2.71)	(-6.39 to 4.30%); 0.70	173, 31.9%
SBQ sub score avoidance					
Treatment cessation	9.2 (8.0-10.3%)	9.5 (8.3-10.7%)	1.14* (0.28)	(0.65 to 2.00%)*; 0.64	215, 15.4%
Follow-up	8.9 (7.4-10.4%)	7.9 (6.2-9.5%)	-1.02 (1.13)	(-3.26 to 1.21%); 0.37	173, 31.9%
ERT - latency overall					
Treatment cessation	2249 (2056-2442%)	2428 (2228-2629%)	179.1 (141.2)	(-99.4 to 457.6%); 0.21	212, 16.5%
Follow-up	2282 (2062-2503%)	1989 (1756-2223%)	-292.7 (163.0)	(-614.8 to 29.3%); 0.075	159, 37.4%
ERT - latency happiness					
Treatment cessation	1011 (949-1072%)	1017 (953-1080%)	6.0 (45.0)	(-82.77 to 94.86%); 0.89	212, 16.5%
Follow-up	1053 (974-1132%)	1045 (962-1128%)	-8.3 (58.3)	(-123.5 to 106.9%); 0.88	159, 37.4%
ERT - latency sadness					

Treatment cessation	1705 (1604-1807%)	1699 (1594-1803%)	-6.68 (73.9)	(-152.5 to 139.1%); 0.93	211, 16.9%
Follow-up	1851 (1665-2037%)	1653 (1457-1849%)	-198.1 (137.0)	(-468.7 to 72.5%); 0.15	158, 37.8%
ERT - latency fear					
Treatment cessation	1820 (1688-1953%)	1860 (1724-1997%)	40.1 (96.7)	(-150.6 to 230.8%); 0.68	210, 17.3%
Follow-up	1713 (1577-1848%)	1686 (1543-1828%)	-27.3 (99.8)	(-224.4 to 169.9%); 0.79	156, 38.6%
ERT - latency anger					
Treatment cessation	1287 (1204-1369%)	1240 (1154-1326%)	-46.9 (60.63)	(-166.5 to 72.6%); 0.44	210, 17.3%
Follow-up	1353 (1241-1466%)	1301 (1182-1422%)	-51.8 (83.5)	(-216.7 to 113.1%); 0.54	158, 37.8%
ERT - latency surprise					
Treatment cessation	1148.1 (1073.3-1222.9%)	1128.6 (1050.9-1206.3%)	-19.50 (54.78)	(-127.51 to 88.50%); 0.72	212, 16.5%
Follow-up	1161.1 (1081.1-1241.1%)	1058.9 (974.2-1143.7%)	-102.16 (59.08)	(-218.88 to 14.55%); 0.086	159, 37.4%
ERT - latency disgust					
Treatment cessation	1709 (1596-1824%)	1747 (1629-1866%)	37.3 (83.5)	(-127.3 to 201.9%); 0.66	210, 17.3%
Follow-up	1665 (1536-1793%)	1644 (1507-1781%)	-21.2 (95.4)	(-209.7 to 167.3%); 0.83	158, 37.8%
ERT - accuracy overall					
Treatment cessation	57.9 (56.6-59.3%)	58.1 (56.7-59.5%)	0.14 (0.98)	(-1.80 to 2.08%); 0.89	212, 16.5%
Follow-up	56.8 (55.4-58.3%)	57.6 (56.0-59.1%)	0.73 (1.07)	(-1.39 to 2.84%); 0.50	159, 37.4%
ERT - accuracy happiness					
Treatment cessation	11.8 (11.4-12.1%)	11.5 (11.2-11.9%)	-0.24 (0.24)	(-0.73 to 0.24%); 0.32	212, 16.5%
Follow-up	11.6 (11.2-12.1%)	11.6 (11.1-12.0%)	-0.08 (0.31)	(-0.69 to 0.54%); 0.81	159, 37.4%
ERT - accuracy sadness					
Treatment cessation	9.6 (9.1-10.1%)	10.2 (9.8-10.7%)	0.66 (0.34)	(-0.02 to 1.33%); 0.057	212, 16.5%

Follow-up	9.6 (9.0-10.1%)	10.5 (9.9-11.1%)	0.89 (0.41)	(0.09 to 1.69%); 0.03**	159, 37.4%
ERT - accuracy fear					
Treatment cessation	6.7 (6.2-7.3%)	6.9 (6.3-7.4%)	0.12 (0.39)	(-0.64 to 0.88%); 0.75	212, 16.5%
Follow-up	6.2 (5.6-6.9%)	6.7 (6.0-7.3%)	0.43 (0.46)	(-0.49 to 1.34%); 0.36	159, 37.4%
ERT - accuracy anger					
Treatment cessation	8.5 (8.1-8.8%)	8.4 (8.0-8.7%)	-0.08 (0.25)	(-0.57 to 0.41%); 0.76	212, 16.5%
Follow-up	8.2 (7.7-8.7%)	8.3 (7.9-8.8%)	0.16 (0.34)	(-0.52 to 0.84%); 0.64	159, 37.4%
ERT - accuracy surprise					
Treatment cessation	11.5 (11.2-11.9%)	11.3 (10.9-11.6%)	-0.22 (0.25)	(-0.72 to 0.28%); 0.38	212, 16.5%
Follow-up	11.4 (11.0-11.8%)	11.3 (10.9-11.7%)	-0.10 (0.30)	(-0.69 to 0.49%); 0.73	159, 37.4%
ERT - accuracy disgust					
Treatment cessation	10.0 (9.5-10.5%)	9.7 (9.1-10.2%)	-0.29 (0.38)	(-1.05 to 0.47%); 0.46	212, 16.5%
Follow-up	9.8 (9.3-10.3%)	9.3 (8.7-9.8%)	-0.55 (0.39)	(-1.31 to 0.21%); 0.16	159, 37.4%
SIAS					
Treatment cessation	41.9 (39.9-43.9%)	42.3 (40.2-44.5%)	0.40 (1.48)	(-2.52 to 3.33%); 0.79	220, 13.4%
Follow-up	39.5 (36.9-42.1%)	38.8 (36.1-41.5%)	-0.69 (1.89)	(-4.43 to 3.04%); 0.71	198, 22.0%

Complete-data-only with numbers of observation and the percentage of missing data. Between-group adjusted mean difference after adjusting for biological sex assigned at birth, study site and dichotomised symptom severity of GPTS subscale *Ideas of Persecution* (≥45 or <45 at baseline). All analyses are linear regression models. All analyses are adjusted for baseline measurement of each outcome. All analyses are conducted without adjustment for baseline imbalances. Values are presented as n(n-n%) indicating the mean value and its 95 % confidence interval. * indicates that a log transformation was applied, and the reported result is therefore an exponentiated, back-transformed value. ** indicates a p value < 0.05. Time points: Treatment cessation, mean = 4.5 months (95%CI 4.3-4.6) after baseline; Follow-up, mean = 10.5 months (95%CI 10.3-10.7) after baseline. GPTS: Green Paranoid Thought Scale. PSP: Personal and Social Performance scale. SBQ: Safety Behavior Questionnaire. ERT: Emotion Recognition Task. SIAS: Social Interaction Anxiety Scale.

Supplementary table 10| Complete-case-only sensitivity analyses: Between-group adjusted mean difference without adjustment for baseline imbalances: Sensitivity analyses on the exploratory outcomes

	VR-CBTp mean (95%CI)	CBTp mean (95%CI)	Adjusted mean difference (standard error)	95 % CI for the adjusted mean difference; p value	Number of observations, % missing data
SAPS Global					
Treatment cessation	6.1 (5.8-6.5%)	6.4 (6.0-6.8%)	0.26 (0.25)	(-0.24 to 0.76%); 0.31	217, 14.6%
Follow-up	5.5 (5.0-5.9%)	5.6 (5.1-6.1%)	0.11 (0.36)	(-0.59 to 0.82%); 0.82	178, 29.9%
SAPS Composite					
Treatment cessation	16.0 (14.8-17.1%)	16.9 (15.8-18.1%)	0.98 (0.83)	(-0.66 to 2.63%); 0.24	217, 14.6%
Follow-up	13.7 (12.3-15.0%)	14.5 (13.0-16.0%)	0.83 (1.03)	(-1.21 to 2.87%); 0.42	178, 29.9%
BNSS total					
Treatment cessation	20.0 (18.9-21.1%)	21.0 (19.8-22.1%)	0.97 (0.81)	(-0.64 to 2.57%); 0.24	216, 15.0%
Follow-up	19.1 (17.5-20.7%)	18.5 (16.8-20.3%)	-0.53 (1.21)	(-2.92 to 1.86%); 0.66	176, 30.7%
CDSS total					
Treatment cessation	4.1 (3.6-4.7%)	4.8 (4.2-5.4%)	0.99* (0.24)	(0.61 to 1.61%)*; 0.98	210, 17.3%
Follow-up	4.5 (3.8-5.2%)	4.8 (4.0-5.5%)	0.33 (0.53)	(-0.71 to 1.37%); 0.53	174, 31.5%
COGDIS total					
Treatment cessation	15.4 (14.0-16.7%)	18.4 (17.0-19.8%)	3.01 (0.99)	(1.06 to 4.95%); 0.003**	209, 17.7%
Follow-up	14.6 (12.8-16.4%)	17.0 (15.0-18.9%)	2.35 (1.35)	(-0.31 to 5.02%); 0.08	176, 30.7%
Trustworthiness task					
Treatment cessation	-0.14 (-0.24 to -0.03%)	-0.09 (-0.20 to 0.02%)	0.05 (0.08)	(-0.10 to 0.20%); 0.52	213, 16.1%
Follow-up	-0.09 (-0.21 to -0.03%)	-0.04 (-0.17 to 0.09%)	0.05 (0.09)	(-0.13 to 0.23%); 0.58	173, 31.9%
SSPA (SCOPE variables)					
Treatment cessation	4.39 (4.31-4.47%)	4.29 (4.21-4.38%)	-0.10 (0.06)	(-0.21 to 0.02%); 0.09	208, 18.1%
Follow-up	4.43 (4.36-4.50%)	4.41 (4.33-4.48%)	-0.02 (0.05)	(-0.12 to 0.08%); 0.67	166, 34.6%
IBT total					
Treatment cessation	0.52 (0.50-0.54%)	0.54 (0.52-0.57%)	0.02 (0.02)	(-0.01 to 0.06%); 0.14	210, 17.3%
Follow-up	0.52 (0.50-0.55%)	0.54 (0.51-0.57%)	0.02 (0.02)	(-0.02 to 0.06%); 0.32	170, 33.1%
IBT Automatic					

Treatment cessation	0.56 (0.52-0.60%)	0.61 (0.57-0.65%)	0.05 (0.03)	(-0.01 to 0.11%); 0.12	210, 17.3%
Follow-up	0.56 (0.52-0.61%)	0.59 (0.54-0.64%)	0.02 (0.03)	(-0.05 to 0.09%); 0.55	170, 33.1%
IBT Control					
Treatment cessation	0.42 (0.38-0.46%)	0.39 (0.35-0.43%)	-0.03 (0.03)	(-0.09 to 0.03%); 0.33	210, 17.3%
Follow-up	0.38 (0.33-0.42%)	0.35 (0.31-0.40%)	-0.02 (0.03)	(-0.08 to 0.04%); 0.50	170, 33.1%
SIDAS					
Treatment cessation	6.0 (4.8-7.3%)	8.3 (7.0-9.7%)	1.51* (0.38)	(0.72 to 3.18%); 0.28*	221, 13.0%
Follow-up	6.1 (4.5-7.6%)	7.7 (6.1-9.3%)	1.66 (1.12)	(-0.54 to 3.86%); 0.14	193, 24.0%
BCSS – negative self					
Treatment cessation	7.6 (7.0-8.3%)	8.5 (7.8-9.2%)	0.85 (0.49)	(-0.11 to 1.81%); 0.08	220, 13.4%
Follow-up	7.0 (6.3-7.8%)	7.8 (7.0-8.6%)	0.79 (0.56)	(-0.31 to 1.89%); 0.16	191, 24.8%
BCSS – negative others					
Treatment cessation	5.7 (5.0-6.4%)	6.6 (5.8-7.3%)	1.13* (0.23)	(0.72 to 1.76%)*; 0.60	220, 13.4%
Follow-up	6.3 (5.5-7.1%)	5.9 (5.1-6.8%)	-0.37 (0.57)	(-1.50 to 0.76%); 0.52	191, 24.8%
BCSS – positive self					
Treatment cessation	8.8 (8.1-9.5%)	8.9 (8.2-9.6%)	0.06 (0.51)	(-0.94 to 1.06%); 0.91	220, 13.4%
Follow-up	9.5 (8.7-10.4%)	9.8 (8.9-10.7%)	0.26 (0.62)	(-0.96 to 1.47%); 0.68	191, 24.8%
BCSS – positive others					
Treatment cessation	10.2 (9.6-10.9%)	9.7 (9.0-10.4%)	-0.54 (0.50)	(-1.54 to 0.45%); 0.28	220, 13.4%
Follow-up	10.5 (9.6-11.4%)	10.4 (9.5-11.3%)	-0.11 (0.66)	(-1.42 to 1.20%); 0.87	191, 24.8%
DACOBS - Jumping to conclusion					
Treatment cessation	22.0 (21.2-22.7%)	21.7 (20.9-22.5%)	-0.23 (0.57)	(-1.36 to 0.89%); 0.68	219, 13.8%
Follow-up	22.1 (21.2-23.0%)	21.2 (20.3 – 22.2%)	-0.89 (0.65)	(-2.18 to 0.40%); 0.18	190, 25.2%
DACOBS - Belief inflexibility					
Treatment cessation	20.3 (19.5-21.0%)	19.9 (19.1-20.7%)	-0.40 (0.55)	(-1.49 to 0.69%); 0.47	219, 13.8%
Follow-up	19.9 (19.0-20.7%)	19.7 (18.8-20.6%)	-0.19 (0.61)	(-1.41 to 1.02%); 0.75	190, 25.2%

DACOBS - Attention for threat					
Treatment cessation	26.9 (26.0-27.8%)	27.6 (26.7-28.5%)	0.71 (0.65)	(-0.58 to 2.00%); 0.28	219, 13.8%
Follow-up	26.2 (25.0-27.4%)	26.8 (25.5-28.0%)	0.57 (0.86)	(-1.13 to 2.27%); 0.51	190, 25.2%
DACOBS - External attribution					
Treatment cessation	19.9 (19.1-20.6%)	20.5 (19.7-21.3%)	0.58 (0.55)	(-0.51 to 1.67%); 0.29	219, 13.8%
Follow-up	19.7 (18.8-20.6%)	19.5 (18.5-20.4%)	-0.23 (0.66)	(-1.54 to 1.07%); 0.73	190, 25.2%
DACOBS - Social cognitive problems					
Treatment cessation	25.8 (24.9-26.7%)	26.6 (25.7-27.6%)	0.78 (0.67)	(-0.55 to 2.11%); 0.25	219, 13.8%
Follow-up	25.3 (24.1-26.4%)	26.2 (25.0-27.4%)	0.92 (0.85)	(-0.75 to 2.59%); 0.28	190, 25.2%
DACOBS - Subjective cognitive problems					
Treatment cessation	26.6 (25.6-27.5%)	26.9 (26.0-27.9%)	0.38 (0.70)	(-1.00 to 1.76%); 0.59	219, 13.8%
Follow-up	26.0 (24.8-27.1%)	27.0 (25.8-28.2%)	1.06 (0.85)	(-0.61 to 2.73%); 0.21	190, 25.2%
DACOBS - Safety behavior					
Treatment cessation	20.0 (18.9-21.0%)	20.4 (19.4-21.5%)	0.47 (0.75)	(-1.01 to 1.96%); 0.53	219, 13.8%
Follow-up	19.2 (18.1-20.4%)	19.0 (17.7-20.2%)	-0.27 (0.86)	(-1.96 to 1.43%); 0.75	190, 25.2%
SFS – Social engagement/withdrawal					
Treatment cessation	97.8 (96.5-99.1%)	96.8 (95.4-98.1%)	-1.02 (0.95)	(-2.90 to 0.85%); 0.28	219, 13.8%
Follow-up	97.9 (96.2-99.6%)	99.2 (97.4-101.1%)	1.36 (1.28)	(-1.16 to 3.88%); 0.29	194, 23.6%
SFS – Interpersonal behavior					
Treatment cessation	114.2 (111.9-116.5%)	112.3 (109.8-114.7%)	-1.94 (1.68)	(-5.26 to 1.38%); 0.25	220, 13.4%
Follow-up	113.9 (111.2-116.5%)	114.6 (111.8-117.4%)	0.75 (1.97)	(-3.15 to 4.64%); 0.71	195, 23.2%
SFS - Recreation					
Treatment cessation	107.2 (105.5-109.0%)	107.2 (105.3-109.0%)	-0.05 (1.29)	(-2.61 to 2.50%); 0.97	218, 14.2%
Follow-up	107.4 (105.0-109.8%)	109.8 (107.3-112.4%)	2.46 (1.76)	(-1.02 to 5.94%); 0.17	193, 24.0%

SFS – Independence-competence					
Treatment cessation	104.2 (102.8-105.7%)	102.4 (100.9-104.0%)	-1.79 (1.08)	(-3.93 to 0.34%); 0.10	217, 14.6%
Follow-up	103.9 (101.9-105.9%)	104.6 (102.5-106.7%)	0.66 (1.48)	(-2.25 to 3.57%); 0.66	192, 24.4%
SFS – Independence-performance					
Treatment cessation	100.3 (98.9-101.7%)	99.7 (98.3-101.2%)	-0.60 (1.02)	(-2.61 to 1.42%); 0.56	220, 13.4%
Follow-up	101.3 (99.5-103.1%)	101.3 (99.4-103.1%)	-0.05 (1.32)	(-2.65 to 2.55%); 0.97	195, 23.2%
SFS – Employment-occupation					
Treatment cessation	104.0 (102.3-105.6%)	104.6 (103.0-106.5%)	0.78 (1.24)	(-1.67 to 3.23%); 0.53	211, 16.9%
Follow-up	104.7 (102.6-106.9%)	104.2 (101.9-106.5%)	-0.52 (1.62)	(-3.72 to 2.68%); 0.75	181, 28.7%
GSE					
Treatment cessation	24.8 (23.9-25.6%)	23.5 (22.6-24.5%)	-1.24 (0.65)	(-2.53 to 0.05%); 0.059	219, 13.8%
Follow-up	24.9 (23.8-25.9%)	24.9 (23.7-26.0%)	0.01 (0.79)	(-1.55 to 1.58%); 0.99	195, 23.2%
EQ-5D-5L					
Treatment cessation	0.63 (0.59-0.67%)	0.57 (0.52-0.61%)	-0.06 (0.03)	(-0.12 to 0.00%); 0.053	214, 15.7%
Follow-up	0.63 (0.59-0.68%)	0.63 (0.58-0.68%)	-0.005 (0.03)	(-0.07 to 0.06%); 0.90	189, 25.6%
EQ5 VAS					
Treatment cessation	60.7 (57.0-64.4%)	57.0 (53.1-61.0%)	-3.63 (2.74)	(-9.04 to 1.78%); 0.19	208, 18.1%
Follow-up	60.6 (56.4-64.9%)	59.5 (55.1-64.0%)	-1.10 (3.13)	(-7.28 to 5.07%); 0.73	181, 28.7%
WHO					
Treatment cessation	41.5 (38.3-44.7%)	40.6 (37.3-44.0%)	-0.89 (2.36)	(-5.54 to 3.76%); 0.71	219, 13.8%
Follow-up	43.9 (39.8 to 48.0%)	44.1 (39.7-48.4%)	0.16 (3.04)	(-5.85 to 6.16%); 0.96	194, 23.6%
GPTS-R – persecution					
Treatment cessation	8.0 (6.8-9.3%)	8.7 (7.4-10.0%)	1.06* (0.28)	(0.61 to 1.83%)*; 0.84	222, 12.6%
Follow-up	7.8 (6.4-9.2%)	7.8 (6.3-9.3%)	0.03 (1.05)	(-2.04 to 2.10%); 0.98	200, 21.3%
GPTS-R – social self-reference					
Treatment cessation	8.5 (7.7-9.4%)	8.6 (7.7-9.6%)	0.09 (0.65)	(-1.19 to 1.37%); 0.89	222, 12.6%
Follow-up	8.0 (6.9-9.1%)	8.5 (7.4-9.6%)	0.52 (0.78)	(-1.02 to 2.07%); 0.51	200, 21.3%

GPTS total					
Treatment cessation	62.5 (59.0-65.9%)	64.3 (60.7-67.9%)	1.84 (2.53)	(-3.15 to 6.83%); 0.47	222, 12.6%
Follow-up	61.2 (56.8-65.5%)	62.2 (57.6-66.8%)	1.03 (3.19)	(-5.27 to 7.33%); 0.75	200, 21.3%
GPTS-R total					
Treatment cessation	16.6 (14.8-18.5%)	17.2 (15.3-19.2%)	0.58 (1.36)	(-2.10 to 3.27%); 0.67	222, 12.6%
Follow-up	15.9 (13.6-18.2%)	16.2 (13.8-18.6%)	0.32 (1.69)	(-3.02 to 3.66%); 0.85	200, 21.3%

Complete-data-only with numbers of observation and the percentage of missing data. Between-group adjusted mean difference after adjusting for biological sex assigned at birth, study site and dichotomised symptom severity of GPTS subscale *Ideas of Persecution* (≥45 or <45 at baseline). All analyses are linear regression models. All analyses are adjusted for baseline measurement of each outcome. All analyses are conducted without adjustment for baseline imbalances. Values are presented as n(n-n%) indicating the mean value and its 95 % confidence interval. * indicates that a log transformation was applied, and the reported result is therefore an exponentiated, back-transformed value. ** indicates a p value < 0.05. Time points: Treatment cessation, mean = 4.5 months (95%CI 4.3-4.6) after baseline; Follow-up, mean = 10.5 months (95%CI 10.3-10.7) after baseline. SAPS: Scale for the Assessment of Positive Symptoms. BNSS: Brief Negative Symptoms Scale. CDSS: Calgary Depression Symptom Scale. COGDIS: Cognitive Disturbances scale. SSPA: Social Skills Performance Assessment. IBT: Intentionality Bias Task. SIDAS: Suicidal Ideation Attributes Scale. BCSS: Brief Core Schema Scale. DACOBS: Davos Assessment of Cognitive Biases Scale. SFS: Social Functioning Scale. GSE: General Self-Efficacy scale. EQ-5D-5L: 5-Level EuroQol 5 Dimensions version. EQ5-VAS: EuroQol Visual Analogue Scale. WHO 5: World Health Organization-Five well-being index. GPTS-R: Revised Green Paranoid Thought Scale. CSQ: Client Satisfaction Questionnaire.

Supplementary table 11| Complete-case-only sensitivity analysis: Between-group adjusted mean difference adjusted for baseline imbalances: Sensitivity analysis on the primary outcome

	VR-CBTp mean (95%CI)	CBTp mean (95%CI)	Adjusted mean difference (standard error)	95 % CI for adjusted mean difference; p value	Number of observations, % missing data
GPTS – Ideas of Persecution					
Treatment cessation	29.5 (27.5-31.5%)	30.7 (28.6-32.8%)	1.02* (0.05)	(0.93 to 1.12%)*; 0.62	219, 13.8%

Complete-data-only with numbers of observation and the percentage of missing data. Between-group adjusted mean difference after adjusting for biological sex assigned at birth, study site and dichotomised symptom severity of GPTS subscale *Ideas of Persecution* (≥45 or <45 at baseline). The analysis is a linear regression model. Analysis is adjusted for baseline measurement of GPTS, *Ideas of Persecution*. The analysis is conducted with adjustment for baseline imbalances. Values are presented as n(n-n%) indicating the mean value and its 95 % confidence. *: Due to the non-normal distribution of the residual plots, a log transformation was applied, which improved the model fit; the reported result is therefore an exponentiated, back-transformed value. Time points: Treatment cessation, mean = 4.5 months (95%CI 4.3-4.6) after baseline; Follow-up, mean = 10.5 months (95%CI 10.3-10.7) after baseline. GPTS: Green Paranoid Thought Scale.

Supplementary table 12| Complete-case-only sensitivity analyses: Between-group adjusted mean difference adjusted for baseline imbalances: Sensitivity analyses on the secondary outcomes

	VR-CBTp mean (95%CI)	CBTp mean (95%CI)	Adjusted mean difference (standard error)	95 % CI for the adjusted mean difference; p value	Number of observations, % missing data
GPTS – Ideas of Persecution					
Follow-up	28.9 (26.4-31.4%)	29.7 (27.1-32.3%)	0.84 (1.87)	(-2.86 to 4.53%); 0.66	197, 22.4%
GPTS – Ideas of Social Self-Reference					
Treatment cessation	33.5 (31.7-35.4%)	33.4 (31.5-35.3%)	-0.14 (1.38)	(-2.86 to 2.58%); 0.92	219, 13.8%
Follow-up	32.5 (30.3-34.8%)	32.7 (30.3-35.0%)	0.14 (1.70)	(-3.20 to 3.49%); 0.93	197, 22.4%
PSP total					
Treatment cessation	48.9 (47.6-50.2%)	49.3 (48.0-50.7%)	0.46 (0.97)	(-1.45 to 2.38%); 0.63	216, 15.0%%
Follow-up	50.5 (48.4-52.5%)	52.0 (49.8-54.2%)	1.53 (1.56)	(-1.54 to 4.60%); 0.33	178, 29.9%
SBQ total					
Treatment cessation	37.1 (34.5-39.8%)	39.2 (36.4-41.9%)	2.03 (1.98)	(-1.88 to 5.93%); 0.31	212, 16.5%
Follow-up	35.7 (32.0-39.4%)	33.0 (28.9-37.0%)	-2.74 (2.87)	(-8.40 to 2.92%); 0.34	170, 33.1%
SBQ sub score avoidance					
Treatment cessation	9.4 (8.2-10.5%)	9.3 (8.0-10.5%)	1.00* (0.30)	(0.55 to 1.80%)*; 1.00	212, 16.5%
Follow-up	9.2 (7.7-10.8%)	7.8 (6.1-9.5%)	-1.44 (1.20)	(-3.82 to 0.94%); 0.23	170, 33.1%
ERT - latency overall					
Treatment cessation	2229 (2034-2425%)	2424 (2222-2625%)	194.6 (146.0)	(-93.2 to 482.4%); 0.18	210, 17.3%
Follow-up	2306 (2075-2536%)	1991 (1751-2231%)	-314.5 (172.3)	(-655.1 to 26.0%); 0.07	156, 38.6%
ERT - latency happiness					
Treatment cessation	1001 (938-1064%)	1017 (952-1082%)	15.6 (47.0)	(-77.0 to 108.2%); 0.74	210, 17.3%
Follow-up	1073 (991-1156%)	1032 (946-1117%)	-41.8 (61.6)	(-163.5 to 79.9%); 0.50	156, 38.6%
ERT - latency sadness					

Treatment cessation	1703 (1598-1807%)	1691 (1584-1798%)	-12.0 (77.8)	(-165.3 to 141.4%); 0.88	209, 17.7%
Follow-up	1888 (1694-2081%)	1623 (1422-1823%)	-264.9 (144.6)	(-550.6 to 20.8%); 0.07	155, 39.0%
ERT - latency fear					
Treatment cessation	1827 (1688-1966%)	1848 (1706-1990%)	21.2 (103.4)	(-182.7 to 225.1%); 0.84	208, 18.1%
Follow-up	1739 (1597-1882%)	1680 (1532-1827%)	-59.3 (106.3)	(-296.5 to 150.8%); 0.59	153, 39.8%
ERT - latency anger					
Treatment cessation	1278 (1197-1360%)	1236 (1152-1320%)	-42.6 (60.93)	(-162.7 to 77.6%); 0.49	208, 18.1%
Follow-up	1350 (1234-1466%)	1303 (1181-1425%)	-47.3 (87.59)	(-220.4 to 125.8%); 0.59	155, 39.0%
ERT - latency surprise					
Treatment cessation	1128 (1053-1204%)	1129 (1052-1207%)	1.00 (56.36)	(-110.1 to 112.1%); 0.99	210, 17.3%
Follow-up	1160 (1077-1242%)	1050 (964-1136%)	-109.7 (61.76)	(-231.8 to 12.31%); 0.08	156, 38.6%
ERT - latency disgust					
Treatment cessation	1677 (1561-1792%)	1781 (1662-1901%)	104.6 (86.7)	(-66.4 to 275.6%); 0.23	208, 18.1%
Follow-up	1677 (1541-1813%)	1641 (1498-1784%)	-35.7 (102.7)	(-238.8 to 167.3%); 0.73	155, 39.0%
ERT - accuracy overall					
Treatment cessation	57.8 (56.4-59.2%)	58.2 (56.8-59.7%)	0.46 (1.05)	(-1.61 to 2.52%); 0.66	210, 17.3%
Follow-up	56.6 (55.0-58.1%)	57.7 (56.1-59.3%)	1.14 (1.14)	(-1.11 to 3.39%); 0.32	156, 38.6%
ERT - accuracy happiness					
Treatment cessation	11.8 (11.4-12.1%)	11.5 (11.2-11.9%)	-0.26 (0.26)	(-0.77 to 0.25%); 0.31	210, 17.3%
Follow-up	11.7 (11.2-12.1%)	11.5 (11.1-12.0%)	-0.14 (0.33)	(-0.80 to 0.52%); 0.68	156, 38.6%
ERT - accuracy sadness					
Treatment cessation	9.5 (9.0-10.0%)	10.3 (9.8-10.8%)	0.78 (0.36)	(0.07 to 1.49%); 0.03**	210, 17.3%

Follow-up	9.6 (9.0-10.1%)	10.6 (10.0-11.2%)	0.99 (0.41)	(0.17 to 1.80%); 0.02**	156, 38.6%
ERT - accuracy fear					
Treatment cessation	6.6 (6.0-7.1%)	7.0 (6.4-7.5%)	0.39 (0.41)	(-0.42 to 1.19%); 0.34	210, 17.3%
Follow-up	6.1 (5.4-6.7%)	6.7 (6.0-7.4%)	0.60 (0.49)	(-0.37 to 1.57%); 0.22	156, 38.6%
ERT - accuracy anger					
Treatment cessation	8.5 (8.1-8.8%)	8.4 (8.0-8.8%)	-0.07 (0.27)	(-0.59 to 0.45%); 0.80	210, 17.3%
Follow-up	8.1 (7.8-8.8%)	8.3 (7.8-8.8%)	0.21 (0.36)	(-0.51 to 0.93%); 0.56	156, 38.6%
ERT - accuracy surprise					
Treatment cessation	11.6 (11.2-11.9%)	11.2 (10.8-11.5%)	-0.40 (0.27)	(-0.93 to 0.12%); 0.13	210, 17.3%
Follow-up	11.4 (11.0-11.8%)	11.2 (10.8-11.7%)	-0.18 (0.32)	(-0.80 to 0.45%); 0.58	156, 38.6%
ERT - accuracy disgust					
Treatment cessation	10.0 (9.5-10.5%)	9.7 (9.1-10.2%)	-0.35 (0.41)	(-1.15 to 0.46%); 0.40	210, 17.3%
Follow-up	9.7 (9.2-10.3%)	9.3 (8.7-9.8%)	-0.49 (0.41)	(-1.29 to 0.32%); 0.24	156, 38.6%
SIAS					
Treatment cessation	42.0 (40.0-44.1%)	42.1 (39.9-44.1%)	0.10 (1.58)	(-3.00 to 3.21%); 0.95	217, 14.6%
Follow-up	39.9 (37.2-42.5%)	38.7 (35.9-41.5%)	-1.15 (2.00)	(-5.10 to 2.79%); 0.57	195, 23.2%

Complete-data-only with numbers of observation and the percentage of missing data. Between-group adjusted mean difference after adjusting for biological sex assigned at birth, study site and dichotomised symptom severity of GPTS subscale *Ideas of Persecution* (≥45 or <45 at baseline). All analyses are linear regression models. All analyses are adjusted for baseline measurement of each outcome. All analyses are conducted with adjustment for baseline imbalances. Values are presented as n(n-n%) indicating the mean value and its 95 % confidence interval. * indicates that a log transformation was applied, and the reported result is therefore an exponentiated, back-transformed value. ** indicates a p value < 0.05. Time points: Treatment cessation, mean = 4.5 months (95%CI 4.3-4.6) after baseline; Follow-up, mean = 10.5 months (95%CI 10.3-10.7) after baseline. GPTS: Green Paranoid Thought Scale. PSP: Personal and Social Performance scale. SBQ: Safety Behavior Questionnaire. ERT: Emotion Recognition Task. SIAS: Social Interaction Anxiety Scale.

Supplementary table 13| Complete-case-only sensitivity analyses: Between-group adjusted mean difference adjusted for baseline imbalances: Sensitivity analyses on the exploratory outcomes

	VR-CBTp mean (95%CI)	CBTp mean (95%CI)	Adjusted mean difference (standard error)	95 % CI for the adjusted mean difference; p value	Number of observations, % missing data
SAPS Global					
Treatment cessation	6.2 (5.8-6.5%)	6.3 (5.9-6.7%)	0.12 (0.27)	(-0.40 to 0.65%); 0.65	214, 15.7%
Follow-up	5.5 (5.0-6.0%)	5.5 (4.9-6.0%)	-0.02 (0.38)	(-0.78 to 0.73%); 0.95	175, 31.1%
SAPS Composite					
Treatment cessation	15.9 (14.7-17.1%)	16.9 (15.6-18.1%)	0.99 (0.89)	(-0.76 to 2.75%); 0.27	214, 15.7%
Follow-up	13.8 (12.3-15.2%)	14.4 (12.8-15.9%)	0.56 (1.10)	(-1.62 to 2.74%); 0.61	175, 31.1%
BNSS total					
Treatment cessation	20.2 (19.0-21.3%)	21.0 (19.8-22.1%)	0.83 (0.86)	(-0.86 to 2.52%); 0.34	213, 16.1%
Follow-up	19.5 (17.8-21.2%)	18.4 (16.6-20.2%)	-1.07 (1.28)	(-3.60 to 1.47%); 0.41	173, 31.9%
CDSS total					
Treatment cessation	4.4 (3.8-5.0%)	4.6 (4.0-5.2%)	0.74* (0.25)	(0.45 to 1.21%)*; 0.23	207, 18.5%
Follow-up	4.6 (3.9-5.4%)	4.7 (3.9-5.5%)	0.06 (0.57)	(-1.07 to 1.18%); 0.92	171, 32.7%
COGDIS total					
Treatment cessation	15.9 (14.5-17.3%)	17.8 (16.4-19.2%)	1.90 (1.02)	(-0.12 to 3.91%); 0.06	207, 18.5%
Follow-up	15.4 (13.6-17.3%)	16.3 (14.4-18.3%)	0.93 (1.41)	(-1.85 to 3.71%); 0.51	173, 31.9%
Trustworthiness task					
Treatment cessation	-0.16 (-0.27 to --0.05%)	-0.08 (-0.19 to 0.03%)	0.08 (0.08)	(-0.08 to 0.24%); 0.32	210, 17.3%
Follow-up	-0.10 (-0.23 to -0.02%)	-0.04 (-0.18 to 0.10%)	0.06 (0.10)	(-0.13 to 0.26%); 0.51	170, 33.1%
SSPA (SCOPE variables)					
Treatment cessation	4.38 (4.30-4.46%)	4.30 (4.21-4.38%)	-0.09 (0.06)	(-0.21 to 0.04%); 0.17	205, 19.3%
Follow-up	4.42 (4.35-4.49%)	4.41 (4.33-4.49%)	-0.01 (0.06)	(-0.12 to 0.10%); 0.87	163, 35.8%
IBT total					
Treatment cessation	0.52 (0.50-0.54%)	0.54 (0.52-0.57%)	0.02 (0.02)	(-0.01 to 0.06%); 0.19	209, 17.7%
Follow-up	0.52 (0.50-0.55%)	0.54 (0.51-0.57%)	0.02 (0.02)	(-0.03 to 0.06%); 0.47	169, 33.5%
IBT Automatic					

Treatment cessation	0.56 (0.52-0.61%)	0.61 (0.57-0.65%)	0.05 (0.03)	(-0.02 to 0.11%); 0.14	209, 17.7%
Follow-up	0.57 (0.52-0.61%)	0.58 (0.53-0.63%)	0.01 (0.04)	(-0.06 to 0.08%); 0.73	169, 33.5%
IBT Control					
Treatment cessation	0.42 (0.38-0.47%)	0.39 (0.35-0.44%)	-0.03 (0.03)	(-0.10 to 0.03%); 0.29	209, 17.7%
Follow-up	0.38 (0.34-0.43%)	0.34 (0.30-0.39%)	-0.04 (0.03)	(-0.11 to 0.02%); 0.20	169, 33.5%
SIDAS					
Treatment cessation	6.3 (4.9-7.6%)	8.2 (6.8-9.6%)	1.51* (0.38)	(0.72 to 3.18%)*; 0.28	218, 14.2%
Follow-up	6.0 (4.5-7.6%)	7.8 (6.1-9.4%)	1.73 (1.19)	(-0.62 to 4.09%); 0.15	190, 25.2%
BCSS – negative self					
Treatment cessation	7.8 (7.1-8.5%)	8.4 (7.7-9.1%)	0.59 (0.52)	(-0.42 to 1.61%); 0.25	217, 14.6%
Follow-up	7.2 (6.4-8.0%)	7.7 (6.9-8.5%)	0.49 (0.59)	(-0.68 to 1.65%); 0.41	189, 25.6%
BCSS – negative others					
Treatment cessation	5.7 (5.0-6.5%)	6.6 (5.8-7.3%)	1.13* (0.24)	(0.71 to 1.82%)*; 0.60	217, 14.6%
Follow-up	6.4 (5.6-7.2%)	5.9 (5.0-6.7%)	-0.51 (0.61)	(-1.71 to 0.70%); 0.41	189, 25.6%
BCSS – positive self					
Treatment cessation	8.5 (7.8-9.2%)	9.0 (8.3-9.7%)	0.48 (0.53)	(-0.56 to 1.53%); 0.36	217, 14.6%
Follow-up	9.3 (8.5-10.2%)	9.9 (9.0-10.8%)	0.60 (0.65)	(-0.67 to 1.87%); 0.35	189, 25.6%
BCSS – positive others					
Treatment cessation	10.0 (9.3-10.7%)	9.8 (9.1-10.5%)	-0.22 (0.53)	(-1.27 to 0.82%); 0.67	217, 14.6%
Follow-up	10.4 (9.5-11.4%)	10.5 (9.5-11.4%)	0.05 (0.71)	(-1.34 to 1.45%); 0.94	189, 25.6%
DACOBS - Jumping to conclusion					
Treatment cessation	21.9 (21.1-22.8%)	21.7 (20.8-22.5%)	-0.29 (0.60)	(-1.48 to 0.90%); 0.63	216, 15.0%
Follow-up	21.8 (20.9-22.7%)	21.4 (20.4 – 22.3%)	-0.46 (0.68)	(-1.80 to 0.89%); 0.50	188, 26.0%
DACOBS - Belief inflexibility					
Treatment cessation	20.3 (19.6-21.1%)	19.8 (19.0-20.6%)	-0.55 (0.58)	(-1.70 to 0.60%); 0.35	216, 15.0%
Follow-up	19.9 (19.0-20.7%)	19.7 (18.8-20.6%)	-0.18 (0.64)	(-1.45 to 1.09%); 0.78	188, 26.0%

DACOBS - Attention for threat					
Treatment cessation	27.0 (26.1-28.0%)	27.5 (26.5-28.4%)	0.43 (0.69)	(-0.93 to 1.79%); 0.53	216, 15.0%
Follow-up	26.3 (25.1-27.5%)	26.8 (25.5-28.0%)	0.44 (0.91)	(-1.37 to 2.24%); 0.63	188, 26.0%
DACOBS - External attribution					
Treatment cessation	19.9 (19.1-20.7%)	20.5 (19.7-21.3%)	0.63 (0.59)	(-0.53 to 1.79%); 0.29	216, 15.0%
Follow-up	19.7 (18.8-20.7%)	19.5 (18.5-20.4%)	-0.27 (0.70)	(-1.66 to 1.12%); 0.70	188, 26.0%
DACOBS - Social cognitive problems					
Treatment cessation	26.0 (25.0-27.0%)	26.4 (25.4-27.4%)	0.42 (0.71)	(-0.99 to 1.82%); 0.56	216, 15.0%
Follow-up	25.6 (24.4-26.8%)	26.0 (24.8-27.3%)	0.42 (0.90)	(-1.35 to 2.19%); 0.64	188, 26.0%
DACOBS - Subjective cognitive problems					
Treatment cessation	26.9 (25.9-27.9%)	26.7 (25.7-27.7%)	-0.17 (0.74)	(-1.62 to 1.29%); 0.82	216, 15.0%
Follow-up	26.3 (25.1-27.5%)	26.8 (25.6-28.1%)	0.51 (0.89)	(-1.25 to 2.27%); 0.57	188, 26.0%
DACOBS - Safety behavior					
Treatment cessation	20.0 (19.0-21.1%)	20.4 (19.3-21.5%)	0.39 (0.79)	(-1.17 to 1.96%); 0.62	216, 15.0%
Follow-up	19.4 (18.2-20.7%)	18.8 (17.6-20.1%)	-0.62 (0.91)	(-2.43 to 1.18%); 0.50	188, 26.0%
SFS – Social engagement/withdrawal					
Treatment cessation	97.6 (96.3-99.0%)	96.8 (95.4-98.2%)	-0.79 (1.01)	(-2.78 to 1.19%); 0.43	216, 15.0%
Follow-up	97.4 (95.6-99.2%)	99.4 (97.5-101.3%)	1.96 (1.33)	(-0.67 to 4.60%); 0.14	191, 24.8%
SFS – Interpersonal behavior					
Treatment cessation	113.4 (111.1-115.7%)	112.8 (110.3-115.2%)	-0.64 (1.76)	(-4.11 to 2.83%); 0.72	217, 14.6%
Follow-up	113.2 (110.4-116.0%)	114.5 (111.6-117.5%)	1.31 (2.09)	(-2.82 to 5.44%); 0.53	192, 24.4%
SFS - Recreation					
Treatment cessation	106.9 (105.1-108.7%)	107.0 (105.1-108.9%)	0.09 (1.35)	(-2.58 to 2.76%); 0.95	215, 15.4%
Follow-up	107.3 (104.8-109.8%)	109.8 (107.2-112.3%)	2.48 (1.87)	(-1.21 to 6.16%); 0.19	190, 25.2%

SFS – Independence-competence					
Treatment cessation	104.2 (102.7-105.7%)	102.4 (100.9-104.0%)	-1.75 (1.15)	(-4.00 to 0.51%); 0.13	214, 15.7%
Follow-up	103.6 (101.5-105.7%)	104.6 (102.4-106.7%)	0.92 (1.56)	(-2.15 to 3.99%); 0.56	189, 25.6%
SFS – Independence-performance					
Treatment cessation	100.0 (98.6-101.4%)	99.7 (98.5-101.5%)	-0.04 (1.08)	(-2.17 to 2.09%); 0.97	217, 14.6%
Follow-up	101.0 (99.2-102.8%)	101.3 (99.4-103.3%)	0.34 (1.37)	(-2.37 to 3.05%); 0.80	192, 24.4%
SFS – Employment-occupation					
Treatment cessation	103.9 (102.2-105.7%)	104.6 (102.8-106.4%)	0.66 (1.31)	(-1.93 to 3.26%); 0.61	208, 18.1%
Follow-up	104.5 (102.2-106.7%)	104.4 (102.1-106.7%)	-0.07 (1.70)	(-3.43 to 3.29%); 0.97	178, 29.9%
GSE					
Treatment cessation	24.5 (23.6-25.4%)	23.6 (22.7-24.6%)	-0.88 (0.68)	(-2.22 to 0.47%); 0.20	216, 15.0%
Follow-up	24.4 (23.3-25.5%)	25.1 (23.9-26.2%)	0.65 (0.82)	(-0.97 to 2.27%); 0.43	192, 24.4%
EQ-5D-5L					
Treatment cessation	0.62 (0.57-0.66%)	0.58 (0.54-0.63%)	-0.04 (0.03)	(-0.10 to 0.03%); 0.27	211, 16.9%
Follow-up	0.62 (0.58-0.67%)	0.64 (0.59-0.69%)	0.01 (0.04)	(-0.06 to 0.08%); 0.74	186, 26.8%
EQ5 VAS					
Treatment cessation	60.4 (56.6-64.2%)	57.6 (53.6-61.6%)	-2.86 (2.90)	(-8.58 to 2.86%); 0.33	205, 19.3%
Follow-up	60.0 (55.6-64.5%)	59.9 (55.4-64.5%)	-0.10 (3.35)	(-6.72 to 6.51%); 0.98	178, 29.9%
WHO					
Treatment cessation	40.3 (37.1-43.5%)	41.5 (38.1-44.8%)	-1.19 (2.42)	(-3.58 to 5.96%); 0.62	216, 15.0%
Follow-up	43.0 (38.7 to 47.2%)	44.5 (40.1-48.9%)	1.54 (3.18)	(-4.73 to 7.82%); 0.63	191, 24.8%
GPTS-R – persecution					
Treatment cessation	8.2 (6.9-9.4%)	8.6 (7.3-10.0%)	0.92 (0.29)	(0.52 to 1.63%)*; 0.78	219, 13.8%
Follow-up	7.7 (6.2-9.2%)	8.0 (6.4-9.5%)	0.24 (1.12)	(-1.98 to 2.46%); 0.83	197, 22.4%
GPTS-R – social self-reference					
Treatment cessation	8.8 (7.9-9.7%)	8.5 (7.6-9.5%)	-0.23 (0.68)	(-1.60 to 1.09%); 0.71	219, 13.8%
Follow-up	8.2 (7.1-9.3%)	8.4 (7.3-9.6%)	0.29 (0.83)	(-1.35 to 1.92%); 0.73	197, 22.4%

GPTS total					
Treatment cessation	63.1 (59.5-66.7%)	64.0 (60.3-67.7%)	0.92 (2.68)	(-4.36 to 6.21%); 0.73	219, 13.8%
Follow-up	61.5 (57.0-66.1%)	62.3 (57.6-67.0%)	0.73 (3.41)	(-5.99 to 7.46%); 0.83	197, 22.4%
GPTS-R total					
Treatment cessation	17.0 (15.1-18.9%)	17.1 (15.1-19.1%)	0.06 (1.44)	(-2.78 to 2.90%); 0.97	219, 13.8%
Follow-up	16.0 (13.6-18.4%)	16.3 (13.8-18.8%)	0.32 (1.81)	(-3.24 to 3.88%); 0.86	197, 22.4%

Complete-data-only with numbers of observation and the percentage of missing data. Between-group adjusted mean difference after adjusting for biological sex assigned at birth, study site and dichotomised symptom severity of GPTS subscale *Ideas of Persecution* (≥45 or <45 at baseline). All analyses are linear regression models. All analyses are adjusted for baseline measurement of each outcome. All analyses are conducted with adjustment for baseline imbalances. Values are presented as n(n-n%) indicating the mean value and its 95 % confidence interval. * indicates that a log transformation was applied, and the reported result is therefore an exponentiated, back-transformed value. Time points: Treatment cessation, mean = 4.5 months (95%CI 4.3-4.6) after baseline; Follow-up, mean = 10.5 months (95%CI 10.3-10.7) after baseline. SAPS: Scale for the Assessment of Positive Symptoms. BNSS: Brief Negative Symptoms Scale. CDSS: Calgary Depression Symptom Scale. COGDIS: Cognitive Disturbances scale. SSPA: Social Skills Performance Assessment. IBT: Intentionality Bias Task. SIDAS: Suicidal Ideation Attributes Scale. BCSS: Brief Core Schema Scale. DACOBS: Davos Assessment of Cognitive Biases Scale. SFS: Social Functioning Scale. GSE: General Self-Efficacy scale. EQ-5D-5L: 5-Level EuroQol 5 Dimensions version. EQ5-VAS: EuroQol Visual Analogue Scale. WHO 5: World Health Organization-Five well-being index. GPTS-R: Revised Green Paranoid Thought Scale. CSQ: Client Satisfaction Questionnaire.

Supplementary table 14| Per-protocol sensitivity analysis: Between-group adjusted mean difference without adjustment for baseline imbalances: Sensitivity analysis on the primary outcome

	VR-CBTp mean (95%CI)	CBTp mean (95%CI)	Adjusted mean difference, (standard error)	95%CI for adjusted mean difference; p value
GPTS – ideas of persecution				
Treatment cessation	29.3 (27.2-31.3%)	31.2 (29.1-33.3%)	1.06* (0.05)	(0.96 to 1.16%)*; 0.27

Only participants receiving full treatment (10 sessions) are analysed (VR-CBTp: n=102, CBTp: n=97). Between-group adjusted mean difference after adjusting for biological sex assigned at birth, study site and dichotomised symptom severity of GPTS subscale *Ideas of Persecution* (≥45 or <45 at baseline). The analysis is a linear regression model handled with multiple imputations. Analysis is adjusted for baseline measurement of GPTS, *Ideas of Persecution*. Analysis is conducted without adjustment for baseline imbalances. *: Due to the non-normal distribution of the residual plots, a log transformation was applied, which improved the model fit; the reported result is therefore an exponentiated, back-transformed value. Values are presented as n(n-n%) indicating the mean value and its 95 % confidence interval. Time points only for participants receiving full treatment: Treatment cessation, mean = 4.5 months (95%CI 4.3-4.7) after baseline; Follow-up, mean = 10.5 months (95%CI 10.2-10.7) after baseline. GPTS: Green Paranoid Thought Scale.

Supplementary table 15| Per-protocol sensitivity analyses: Between-group adjusted mean difference without adjustment for baseline imbalances: Sensitivity analyses on the secondary outcomes

	VR-CBTp mean (95%CI)	CBTp mean (95%CI)	Adjusted mean difference (standard error)	95%CI for adjusted mean difference; p value
GPTS – Ideas of Persecution				
Follow-up	28.8 (26.2-31.4%)	30.9 (28.1-33.6%)	2.04 (1.91)	(-1.72 to 5.80%); 0.29
GPTS – Ideas of Social Self-Reference				
Treatment cessation	32.6 (30.7-34.4%)	33.9 (31.9-35.8%)	1.30 (1.35)	(-1.37 to 3.97%) ; 0.34
Follow-up	32.3 (29.9-34.6%)	33.7 (31.2-36.2%)	1.43 (1.74)	(-2.01 to 4.87%) ; 0.41
PSP total				
Treatment cessation	49.8 (48.5-51.2%)	49.4 (48.0-50.8%)	-0.42 (1.00)	(-2.40 to 1.56%) ; 0.68
Follow-up	51.9 (49.8-54.1%)	52.4 (50.0-54.8%)	0.48 (1.63)	(-2.74 to 3.70%) ; 0.77
SBQ total				
Treatment cessation	36.5 (33.8-39.3%)	39.0 (36.1-41.9%)	2.48 (2.01)	(-1.51 to 6.48%) ; 0.22
Follow-up	34.4 (30.8-38.1%)	34.5 (30.3-38.7%)	0.07 (2.85)	(-5.57 to 5.70%) ; 0.98
SBQ sub-score avoidance				
Treatment cessation	9.1 (7.8-10.3%)	9.4 (8.1-10.7%)	1.13* (0.30)	(0.63 to 2.02%)* ; 0.69
Follow-up	8.5 (7.0-10.1%)	8.1 (6.4-9.9%)	-0.40 (1.18)	(-2.73 to 1.94%) ; 0.74
ERT - latency overall				
Treatment cessation	2257 (2062-2452%)	2422 (2221-2622%)	164.6 (142.2)	(-116.0 to 445.2%) 0.25
Follow-up	2331 (2055-2606%)	2019 (1764-2275%)	-311.1 (188.8)	(-684.5 to 62.4%) ; 0.10
ERT - latency happiness				
Treatment cessation	1013 (945-1081%)	1019 (950-1088%)	5.87 (49.4)	(-91.7 to 103.4%) ; 0.91
Follow-up	1039 (963-1115%)	1009 (930-1089%)	-29.9 (55.1)	(-138.8 to 80.0%); 0.59
ERT - latency sadness				
Treatment cessation	1762 (1646-1879%)	1749 (1631-1868%)	-13.1 (84.0)	(-178.8 to 152.6%); 0.88
Follow-up	1792 (1593-1991%)	1643 (1445-1841%)	-148.7 (142.6)	(-430.6 to 133.2%); 0.30
ERT - latency fear				

Treatment cessation	1872 (1726-2018%)	1919 (1766-2073%)	47.3 (107.0)	(-163.9 to 258.4%); 0.66
Follow-up	1680 (1537-1824%)	1717 (1569-1864%)	36.2 (104.0)	(-169.2 to 241.6%); 0.73
ERT - latency anger				
Treatment cessation	1313 (1222-1404%)	1273 (1179-1366%)	-40.4 (66.5)	(-171.7 to 90.8%); 0.54
Follow-up	1353 (1236-1469%)	1254 (1133-1376%)	-98.0 (86.9)	(-269.9 to 73.8%); 0.30
ERT - latency surprise				
Treatment cessation	1163 (1079-1247%)	1145 (1059-1230%)	-18.2 (61.1)	(-138.8 to 102.4%); 0.77
Follow-up	1139 (1059-1219%)	1055 (974-1135%)	-84.4 (57.4)	(-197.8 to 28.9%); 0.14
ERT - latency disgust				
Treatment cessation	1750 (1625-1875%)	1756 (1624-1888%)	6.19 (92.2)	(-175.8 to 188.2%); 0.95
Follow-up	1669 (1525-1812%)	1627 (1485-1770%)	-41.2 (103.0)	(-245.0 to 162.5%); 0.69
ERT - accuracy overall				
Treatment cessation	57.7 (56.2-59.1%)	57.5 (56.1-59.0%)	-0.12 (1.04)	(-2.16 to 1.93%); 0.92
Follow-up	57.0 (55.5-58.5%)	58.3 (56.7-59.9%)	1.31 (1.09)	(-0.84 to 3.46%); 0.23
ERT - accuracy happiness				
Treatment cessation	11.6 (11.3-12.0%)	11.5 (11.1-11.9%)	-0.13 (0.26)	(-0.64 to 0.37%); 0.60
Follow-up	11.5 (11.1-12.0%)	11.6 (11.1-12.1%)	0.10 (0.34)	(-0.57 to 0.78%); 0.76
ERT - accuracy sadness				
Treatment cessation	9.5 (9.0-10.1%)	10.1 (9.6-10.7%)	0.60 (0.37)	(-0.13 to 1.33%); 0.10
Follow-up	9.5 (8.9-10.1%)	10.6 (10.0-11.2%)	1.10 (0.42)	(0.28 to 1.93%); 0.009**
ERT - accuracy fear				
Treatment cessation	6.6 (6.1-7.2%)	6.9 (6.3-7.4%)	0.24 (0.40)	(-0.55 to 1.04%); 0.55
Follow-up	6.3 (5.7-7.0%)	6.9 (6.1-7.6%)	0.53 (0.51)	(-0.49 to 1.55%); 0.30
ERT - accuracy anger				
Treatment cessation	8.4 (8.0-8.8%)	8.2 (7.9-8.6%)	-0.14 (0.27)	(-0.67 to 0.40%); 0.61
Follow-up	8.3 (7.8-8.9%)	8.4 (7.9-9.0%)	0.09 (0.38)	(-0.66 to 0.83%); 0.81

ERT - accuracy surprise				
Treatment cessation	11.6 (11.2-12.0%)	11.2 (10.8-11.5%)	-0.44 (0.27)	(-0.98 to 0.09%); 0.10
Follow-up	11.2 (10.8-11.7%)	11.2 (10.7-11.6%)	-0.06 (0.31)	(-0.68 to 0.56%); 0.85
ERT - accuracy disgust				
Treatment cessation	9.9 (9.3-10.4%)	9.6 (9.0-10.1%)	-0.29 (0.41)	(-1.10 to 0.52%); 0.48
Follow-up	9.9 (9.4-10.5%)	9.6 (9.0-10.2%)	-0.32 (0.42)	(-1.14 to 0.50%); 0.45
SIAS				
Treatment cessation	41.6 (39.4-43.7%)	42.1 (39.8-44.4%)	0.53 (1.60)	(-2.64 to 3.70%); 0.74
Follow-up	40.1 (37.4-42.7%)	38.7 (35.9-41.4%)	-1.39 (1.95)	(-5.23 to 2.45%); 0.48

Only participants receiving full treatment (10 sessions) are analysed (VR-CBTp: n=102, CBTp: n=97). Between-group adjusted mean difference after adjusting for biological sex assigned at birth, study site and dichotomised symptom severity of GPTS subscale *Ideas of Persecution* (≥45 or <45 at baseline). All analyses are linear regression models handled with multiple imputations. All analyses are adjusted for baseline measurement of each outcome. All analyses are conducted without adjustment for baseline imbalances. Values are presented as n(n-n%), indicating the mean value and its 95 % confidence interval. * indicates that a log transformation was applied, and the reported result is therefore an exponentiated, back-transformed value. ** indicates a p value < 0.05. Time points only for participants receiving full treatment: Treatment cessation, mean = 4.5 months (95%CI 4.3-4.7) after baseline; Follow-up, mean = 10.5 months (95%CI 10.2-10.7) after baseline. GPTS: Green Paranoid Thought Scale. PSP: Personal and Social Performance scale. SBQ: Safety Behavior Questionnaire. ERT: Emotion Recognition Task. SIAS: Social Interaction Anxiety Scale.

Supplementary table 16| Per-protocol sensitivity analyses: Between-group adjusted mean difference without adjustment for baseline imbalances: Sensitivity analyses on the exploratory outcomes

Variable	VR-CBTp mean (95%CI)	CBTp mean (95%CI)	Adjusted mean difference (standard error)	95%CI for adjusted mean difference; p value
SAPS Global				
Treatment cessation	6.3 (5.9-6.6%)	6.5 (6.1-6.9%)	0.27 (0.26)	(-0.24 to 0.79%); 0.30
Follow-up	5.5 (5.0-6.0%)	5.8 (5.3-6.4%)	0.34 (0.39)	(-0.44 to 1.12%); 0.39
SAPS Composite				
Treatment cessation	16.1 (14.9-17.3%)	17.5 (16.2-18.8%)	1.40 (0.90)	(-0.38 to 3.17%); 0.12
Follow-up	13.8 (12.4-15.3%)	14.9 (13.3-16.5%)	1.05 (1.09)	(-1.10 to 3.20%); 0.34
BNSS total				
Treatment cessation	20.0 (18.8-21.2%)	21.0 (19.7-22.2%)	1.00 (0.87)	(-0.72 to 2.72%); 0.25
Follow-up	19.3 (17.6-21.0%)	18.8 (16.9-20.7%)	-0.54 (1.29)	(-3.09 to 2.01%); 0.68
CDSS total				
Treatment cessation	4.3 (3.7-4.9%)	4.8 (4.2-5.5%)	0.95* (0.25)	(0.58 to 1.56%)*; 0.85
Follow-up	4.8 (4.1-5.6%)	4.8 (3.9-5.7%)	0.01 (0.58)	(-1.14 to 1.15%); 0.99
COGDIS total				
Treatment cessation	15.4 (14.0-16.8%)	17.9 (16.4-19.4%)	2.48 (1.05)	(0.42 to 4.55%) 0.019**
Follow-up	15.7 (13.8-17.5%)	16.4 (14.3-18.5%)	0.72 (1.42)	(-2.09 to 3.53%); 0.61
Trustworthiness task				
Treatment cessation	-0.14 (-0.25 to -0.04%)	-0.12 (-0.23 to -0.01%)	0.03 (0.08)	(-0.13 to 0.18%); 0.74
Follow-up	-0.12 (-0.23 to 0.00%)	-0.12 (-0.25 to 0.01%)	-0.002 (0.09)	(-0.17 to 0.17%); 0.98
SSPA (SCOPE variables)				
Treatment cessation	4.40 (4.31-4.49%)	4.29 (4.20-4.38%)	-0.11 (0.06)	(-0.24 to 0.02%); 0.09
Follow-up	4.44 (4.36-4.52%)	4.38 (4.28-4.47%)	-0.07 (0.06)	(-0.19 to 0.06%); 0.29
IBT total				
Treatment cessation	0.52 (0.49-0.54%)	0.54 (0.51-0.56%)	0.02 (0.02)	(-0.02 to 0.05%); 0.27
Follow-up	0.53 (0.51-0.56%)	0.53 (0.50-0.56%)	-0.002 (0.02)	(-0.04 to 0.04%); 0.93
IBT Automatic				
Treatment cessation	0.56 (0.52-0.60%)	0.60 (0.56-0.65%)	0.04 (0.03)	(-0.02 to 0.11%); 0.16

Follow-up	0.59 (0.54-0.63%)	0.57 (0.52-0.62%)	-0.02 (0.04)	(-0.09 to 0.05%); 0.65
IBT Control				
Treatment cessation	0.42 (0.37-0.46%)	0.39 (0.35-0.44%)	-0.02 (0.03)	(-0.09 to 0.04%); 0.49
Follow-up	0.39 (0.35-0.43%)	0.38 (0.33-0.43%)	-0.01 (0.03)	(-0.08 to 0.06%); 0.77
SIDAS				
Treatment cessation	6.3 (4.9-7.7%)	8.6 (7.1-10.0%)	1.62* (0.40)	(0.73 to 3.60%); 0.24*
Follow-up	6.8 (5.2-8.3%)	8.7 (7.1-10.4%)	1.96 (1.16)	(-0.33 to 4.24%); 0.09
BCSS – negative self				
Treatment cessation	7.7 (7.1-8.3%)	8.2 (7.5-8.9%)	0.51 (0.47)	(-0.42 to 1.44%); 0.28
Follow-up	7.3 (6.5-8.2%)	7.8 (7.0-8.7%)	0.49 (0.60)	(-0.70 to 1.67%); 0.42
BCSS – negative others				
Treatment cessation	5.5 (4.8-6.3%)	6.4 (5.6-7.2%)	1.08* (0.25)	(0.66 to 1.77%)*; 0.76
Follow-up	6.3 (5.4-7.1%)	6.1 (5.2-7.0%)	-0.14 (0.63)	(-1.38 to 1.11%); 0.83
BCSS – positive self				
Treatment cessation	8.8 (8.0-9.5%)	8.8 (8.1-9.6%)	0.06 (0.53)	(-0.98 to 1.11%); 0.90
Follow-up	9.2 (8.4-10.1%)	9.6 (8.7-10.6%)	0.39 (0.64)	(-0.87 to 1.65%); 0.54
BCSS – positive others				
Treatment cessation	10.2 (9.5-11.0%)	9.9 (9.1-10.7%)	-0.33 (0.54)	(-1.40 to 0.75%); 0.55
Follow-up	10.5 (9.6-11.5%)	10.3 (9.3-11.3%)	-0.19 (0.71)	(-1.59 to 1.20%); 0.78
DACOBS - Jumping to conclusion				
Treatment cessation	21.8 (21.0-22.7%)	21.8 (20.9-22.7%)	-0.01 (0.62)	(-1.24 to 1.22%); 0.98
Follow-up	22.0 (21.0-23.0%)	21.6 (20.6-22.6%)	-0.41 (0.72)	(-1.84 to 1.02%); 0.57
DACOBS - Belief inflexibility				
Treatment cessation	20.5 (19.7-21.3%)	19.9 (19.1-20.7%)	-0.65 (0.58)	(-1.80 to 0.51%); 0.27
Follow-up	19.9 (19.0-20.7%)	19.8 (18.9-20.7%)	-0.11 (0.64)	(-1.38 to 1.16%); 0.87
DACOBS - Attention for threat				
Treatment cessation	27.0 (26.0-27.9%)	27.7 (26.8-28.7%)	0.78 (0.68)	(-0.55 to 2.11%); 0.25
Follow-up	26.1 (24.9-27.3%)	28.8 (25.5-28.2%)	0.73 (0.92)	(-1.08 to 2.54%); 0.43
DACOBS - External attribution				
Treatment cessation	19.8 (18.9-20.6%)	20.6 (19.7-21.4%)	0.82 (0.59)	(-0.36 to 1.99%); 0.17
Follow-up	19.6 (18.6-20.6%)	19.6 (18.6-20.6%)	-0.01 (0.72)	(-1.44 to 1.41%); 0.98

DACOBS - Social cognitive problems				
Treatment cessation	25.9 (24.9-26.9%)	26.5 (25.5-27.6%)	0.62 (0.72)	(-0.81 to 2.04%); 0.40
Follow-up	25.3 (24.1-26.5%)	26.4 (25.1-27.7%)	1.08 (0.89)	(-0.68 to 2.83%); 0.23
DACOBS - Subjective cognitive problems				
Treatment cessation	26.4 (25.4-27.5%)	26.9 (25.8-27.9%)	0.45 (0.76)	(-1.05 to 1.94%); 0.55
Follow-up	26.3 (25.1-27.5%)	26.8 (25.5-28.0%)	0.51 (0.88)	(-1.23 to 2.25%); 0.56
DACOBS - Safety behavior				
Treatment cessation	20.2 (19.1-21.3%)	20.2 (19.1-21.3%)	0.03 (0.80)	(-1.55 to 1.62%); 0.97
Follow-up	19.3 (18.0-20.5%)	1868 (17.3-20.0%)	-0.64 (0.94)	(-2.49 to 1.22%); 0.50
SFS – Social engagement/withdrawal				
Treatment cessation	97.7 (96.3-99.0%)	96.8 (95.4-98.2%)	-0.85 (0.97)	(-2.76 to 1.06%); 0.38
Follow-up	97.5 (95.7-99.3%)	99.0 (97.1-100.9%)	1.51 (1.33)	(-1.11 to 4.13%); 0.26
SFS – Interpersonal behavior				
Treatment cessation	114.5 (112.0-116.9%)	112.0 (109.4-114.5%)	-2.49 (1.80)	(-6.06 to 1.07%); 0.17
Follow-up	114.1 (111.2-116.9%)	114.6 (111.6-117.5%)	0.50 (2.08)	(-3.62 to 4.61%); 0.81
SFS - Recreation				
Treatment cessation	107.4 (105.5-109.2%)	107.3 (105.4-109.3%)	-0.03 (1.37)	(-2.74 to 2.67%); 0.98
Follow-up	107.0 (104.5-109.5%)	109.6 (106.9-112.2%)	2.53 (1.85)	(-1.12 to 6.18%); 0.17
SFS – Independence-competence				
Treatment cessation	103.6 (102.1-105.2%)	103.0 (101.3-104.6%)	-0.68 (1.16)	(-2.96 to 1.61%); 0.56
Follow-up	104.2 (102.2-106.3%)	104.4 (102.2-106.6%)	0.14 (1.54)	(-2.90 to 3.17%); 0.93
SFS – Independence-performance				
Treatment cessation	100.3 (98.9-101.8%)	99.6 (98.0-101.1%)	-0.78 (1.07)	(-2.90 to 1.34%); 0.47
Follow-up	101.4 (99.5-103.3%)	101.5 (99.4-103.6%)	0.05 (1.42)	(-2.74 to 2.85%); 0.97
SFS – Employment-occupation				
Treatment cessation	104.2 (102.5-105.8%)	104.8 (103.1-106.5%)	0.64 (1.23)	(-1.79 to 3.07%); 0.61
Follow-up	105.8 (103.6-108.1%)	104.6 (102.2-107.0%)	-1.29 (1.66)	(-4.57 to 2.00%); 0.44
GSE				
Treatment cessation	24.4 (23.5-25.3%)	23.4 (22.4-24.4%)	-1.05 (0.69)	(-2.40 to 0.31%); 0.13

Follow-up	24.5 (23.4-25.6%)	24.7 (23.6-25.9%)	0.26 (0.82)	(-1.37 to 1.88%); 0.76
EQ-5D-5L				
Treatment cessation	0.64 (0.59-0.68%)	0.56 (0.52-0.61%)	-0.07 (0.03)	(-0.14 to -0.01%); 0.025**
Follow-up	0.65 (0.60-0.69%)	0.65 (0.60-0.69%)	0.001 (0.03)	(-0.06 to 0.07%); 0.97
EQ5 VAS				
Treatment cessation	61.3 (57.3-65.3%)	56.1 (51.9-60.3%)	-5.20 (2.94)	(-11.00 to 0.60%); 0.08
Follow-up	61.7 (57.3-66.1%)	59.3 (54.7-64.0%)	-2.36 (3.27)	(-8.81 to 4.09%); 0.47
WHO 5				
Treatment cessation	41.6 (38.2-45.1%)	40.8 (37.2-44.4%)	-0.80 (2.55)	(-5.83 to 4.22%); 0.75
Follow-up	43.4 (39.1-47.7%)	43.2 (38.6-47.7%)	-0.20 (3.18)	(-6.47 to 6.07%); 0.95
R-GPTS – Ideas of Persecution				
Treatment cessation	8.0 (6.7-9.3%)	8.8 (7.5-10.2%)	1.08 (0.29)	(0.60 to 1.93%)*; 0.80
Follow-up	7.8 (6.2-9.3%)	8.7 (7.1-10.3%)	0.93 (1.16)	(-1.36 to 3.22%); 0.42
R-GPTS – Ideas of Social Self-Reference				
Treatment cessation	8.3 (7.4-9.2%)	8.7 (7.8-9.7%)	0.44 (0.67)	(-0.88 to 1.77%); 0.51
Follow-up	8.1 (6.9-9.2%)	8.9 (7.7-10.1%)	0.81 (0.85)	(-0.88 to 2.50%); 0.35
GPTS total				
Treatment cessation	61.9 (58.3-65.5%)	64.9 (61.2-68.6%)	2.98 (2.63)	(-2.22 to 8.17%); 0.26
Follow-up	61.0 (56.3-65.7%)	64.4 (59.4-69.4%)	3.38 (3.50)	(-3.53 to 10.30%); 0.34
R-GPTS total				
Treatment cessation	16.4 (14.4-18.3%)	17.5 (15.5-19.5%)	1.12 (1.41)	(-1.67 to 3.90%); 0.43
Follow-up	15.9 (13.3-18.4%)	17.5 (14.8-20.2%)	1.64 (1.87)	(-2.05 to 5.33%); 0.38
CSQ				
Treatment cessation	27.3 (26.6-28.0%)	26.2 (25.5-27.0%)	-0.09 (0.54)	(-1.16 to 0.97%); 0.86

Only participants receiving full treatment (10 sessions) are analysed (VR-CBTp: n=102, CBTp: n=97). Between-group adjusted mean difference after adjusting for biological sex assigned at birth, study site and dichotomised symptom severity of GPTS subscale *Ideas of Persecution* (≥45 or <45 at baseline). All analyses are conducted without adjustment for baseline imbalances. All analyses are linear regression models handled with multiple imputations. All analyses are adjusted for baseline measurement of each outcome, except for the CSQ that was not administered at baseline. For CSQ, a linear regression model adjusted for biological sex assigned at birth, study site and dichotomised symptom severity of GPTS subscale *Ideas of Persecution* (≥45 or <45 at baseline) was used. Values are presented as n(n-n%), indicating the mean value and its 95 % confidence interval. * indicates that a log transformation was applied, and the reported result is therefore an exponentiated, back-transformed value. ** indicates a p value < 0.05. Time points only for participants receiving full treatment: Treatment cessation, mean = 4.5 months (95%CI 4.3-4.7) after baseline; Follow-up, mean = 10.5 months (95%CI 10.2-10.7) after baseline. SAPS: Scale for the Assessment of Positive Symptoms. BNSS: Brief Negative Symptoms Scale. CDSS: Calgary Depression Symptom Scale. COGDIS: Cognitive Disturbances scale. SSPA:

Social Skills Performance Assessment. IBT: Intentionality Bias Task. SIDAS: Suicidal Ideation Attributes Scale. BCSS: Brief Core Schema Scale. DACOBS: Davos Assessment of Cognitive Biases Scale. SFS: Social Functioning Scale. GSE: General Self-Efficacy scale. EQ-5D-5L: 5-Level EuroQol 5 Dimensions version. EQ5-VAS: EuroQol Visual Analogue Scale. WHO 5: World Health Organization-Five well-being index. R-GPTS: Revised Green Paranoid Thought Scale. CSQ: Client Satisfaction Questionnaire.

Supplementary table 17| Per-protocol sensitivity analyses: Between-group adjusted mean difference adjusted for baseline imbalances: Sensitivity analysis on the primary outcome

	VR-CBTp mean (95%CI)	CBTp mean (95%CI)	Adjusted mean difference (standard error)	95%CI for adjusted mean difference; p value
GPTS – Ideas of Persecution				
Treatment cessation	29.3 (27.3-31.4%)	31.1 (28.9-33.3%)	1.04* (0.05)	(0.94 to 1.15%)*; 0.46

Only participants receiving full treatment (10 sessions) are analysed (VR-CBTp: n=102, CBTp: n=97). Between-group adjusted mean difference after adjusting for biological sex assigned at birth, study site and dichotomised symptom severity of GPTS subscale *Ideas of Persecution* (≥45 or <45 at baseline). The analysis is a linear regression handled with multiple imputations. Analysis is adjusted for baseline measurement of GPTS, ideas of Persecution. Analysis is conducted with adjustment for baseline imbalances. Values are presented as n(n-n%) indicating the mean value and its 95 % confidence interval. *: Due to the non-normal distribution of the residual plots, a log transformation was applied, which improved the model fit; the reported result is therefore an exponentiated, back-transformed value. Time points only for participants receiving full treatment: Treatment cessation, mean = 4.5 months (95%CI 4.3-4.7) after baseline; Follow-up, mean = 10.5 months (95%CI 10.2-10.7) after baseline. GPTS: Green Paranoid Thought Scale.

Supplementary table 18| Per-protocol sensitivity analyses: Between-group adjusted mean difference adjusted for baseline imbalances: Sensitivity analyses on the secondary outcomes

	VR-CBTp mean (95%CI)	CBTp mean (95%CI)	Adjusted mean difference (standard error)	95%CI for adjusted mean difference; p value
GPTS – Ideas of Persecution				
Follow-up	28.7 (26.1-31.4%)	31.0 (28.2-33.8%)	2.25 (1.99)	(-1.68 to 6.18%); 0.26
GPTS – Ideas of Social Self-Reference				
Treatment cessation	32.8 (30.9-34.6%)	33.7 (31.7-35.6%)	0.90 (1.41)	(-1.89 to 3.68%); 0.53
Follow-up	32.4 (30.1-34.8%)	33.5 (31.0-36.1%)	1.08 (1.80)	(-2.48 to 4.63%); 0.55
PSP total				
Treatment cessation	49.5 (48.2-50.9%)	49.8 (48.3-51.2%)	0.23 (1.04)	(-1.81 to 2.28%); 0.82
Follow-up	51.5 (49.3-53.6%)	52.9 (50.5-55.3%)	1.44 (1.68)	(-1.89 to 4.76%); 0.39
SBQ total				
Treatment cessation	36.8 (34.0-39.6%)	38.7 (35.8-41.7%)	1.96 (2.10)	(-2.17 to 6.10%); 0.35
Follow-up	34.7 (31.0-38.5%)	34.2 (29.9-38.6%)	-0.53 (3.00)	(-6.45 to 5.39%); 0.86
SBQ sub score avoidance				
Treatment cessation	9.2 (7.9-10.5%)	9.2 (7.9-10.6%)	1.02* (0.31)	(0.56 to 1.86%)*; 0.95
Follow-up	8.6 (7.0-10.2%)	8.1 (6.3-9.9%)	-0.51 (1.24)	(-2.97 to 1.95%); 0.68
ERT - latency overall				
Treatment cessation	2268 (2069-2467%)	2410 (2206-2615%)	142.4 (148.0)	(-149.6 to 434.5%); 0.34
Follow-up	2328 (2052-2603%)	2023 (1757-2288%)	-305.1 (195.8)	(-692.4 to 82.3%); 0.12
ERT - latency happiness				
Treatment cessation	1010 (940-1080%)	1023 (951-1094%)	12.7 (52.0)	(-89.9 to 115.2%); 0.81
Follow-up	1051 (975-1127%)	997 (916-1077%)	-54.5 (56.4)	(-166.0 to 57.1%); 0.34
ERT - latency sadness				
Treatment cessation	1779 (1659-1899%)	1732 (1611-1853%)	-47.1 (88.1)	(-221.0 to 126.8%); 0.59
Follow-up	1824 (1626-2021%)	1610 (1410-1810%)	-213.8 (144.6)	(-499.5 to 72.0%); 0.14
ERT - latency fear				

Treatment cessation	1883 (1732-2033%)	1909 (1750-2067%)	26.0 (113.2)	(-197.5 to 249.4%); 0.82
Follow-up	1697 (1553-1842%)	1699 (1547-1851%)	1.83 (107.8)	(-211.2 to 214.8%); 0.99
ERT - latency anger				
Treatment cessation	1318 (1225-1411%)	1268 (1172-1363%)	-50.3 (69.3)	(-187.1 to 86.5%); 0.47
Follow-up	1352 (1234-1469%)	1255 (1130-1381%)	-96.5 (90.0)	(-274.6 to 81.7%); 0.29
ERT - latency surprise				
Treatment cessation	1150 (1065-1236%)	1158 (1071-1245%)	7.53 (63.5)	(-117.8 to 132.8%); 0.91
Follow-up	1148 (1067-1229%)	1045 (964-1126%)	-103.1 (58.9)	(-219.3 to 13.2%); 0.08
ERT - latency disgust				
Treatment cessation	1720 (1596-1844%)	1788 (1655-1920%)	67.5 (94.4)	(-118.8 to 253.9%); 0.48
Follow-up	1677 (1532-1822%)	1618 (1472-1764%)	-59.3 (105.9)	(-268.7 to 150.2%); 0.58
ERT - accuracy overall				
Treatment cessation	57.6 (56.1-59.1%)	57.6 (56.1-59.1%)	0.00 (1.09)	(-2.14 to 2.15%); 1.00
Follow-up	56.9 (55.4-58.4%)	58.4 (56.7-60.0%)	1.50 (1.13)	(-0.73 to 3.74%); 0.19
ERT - accuracy happiness				
Treatment cessation	11.7 (11.3-12.0%)	11.5 (11.1-11.8%)	-0.20 (0.27)	(-0.73 to 0.33%); 0.46
Follow-up	11.6 (11.1-12.0%)	11.6 (11.1-12.1%)	0.04 (0.35)	(-0.66 to 0.74%); 0.91
ERT - accuracy sadness				
Treatment cessation	9.5 (9.0-10.0%)	10.2 (9.7-10.7%)	0.72 (0.38)	(-0.03 to 1.48%); 0.06
Follow-up	9.4 (8.9-10.0%)	10.6 (10.1-11.2%)	1.20 (0.42)	(0.37 to 2.03%); 0.005**
ERT - accuracy fear				
Treatment cessation	6.5 (5.9-7.0%)	7.0 (6.4-7.6%)	0.52 (0.42)	(-0.30 to 1.34%); 0.22
Follow-up	6.3 (5.6-7.0%)	6.9 (6.2-7.7%)	0.64 (0.53)	(-0.41 to 1.69%); 0.23
ERT - accuracy anger				
Treatment cessation	8.4 (8.0-8.8%)	8.2 (7.8-8.6%)	-0.19 (0.29)	(-0.75 to 0.37%); 0.51
Follow-up	8.3 (7.8-8.9%)	8.4 (7.9-9.0%)	0.10 (0.39)	(-0.67 to 0.87%); 0.80

ERT - accuracy surprise				
Treatment cessation	11.7 (11.3-12.1%)	11.1 (10.7-11.5%)	-0.62 (0.28)	(-1.18 to -0.05%); 0.03**
Follow-up	11.3 (10.8-11.7%)	11.1 (10.7-11.6%)	-0.13 (0.32)	(-0.77 to 0.52%); 0.70
ERT - accuracy disgust				
Treatment cessation	9.9 (9.3-10.5%)	9.5 (8.9-10.1%)	-0.42 (0.43)	(-1.27 to 0.42%); 0.32
Follow-up	9.9 (9.4-10.5%)	9.6 (9.0-10.2%)	-0.31 (0.43)	(-1.15 to 0.53%); 0.47
SIAS				
Treatment cessation	41.6 (39.4-43.8%)	42.0 (39.7-44.4%)	0.42 (1.68)	(-2.90 to 3.74%); 0.80
Follow-up	39.9 (37.2-42.6%)	38.8 (36.0-41.6%)	-1.07 (2.01)	(-5.04 to 2.90%); 0.59

Only participants receiving full treatment (10 sessions) are analysed (VR-CBTp: n=102, CBTp: n=97). Between-group adjusted mean difference after adjusting for biological sex assigned at birth, study site and dichotomised symptom severity of GPTS subscale *Ideas of Persecution* (≥45 or <45 at baseline). All analyses are linear regression models handled with multiple imputations. All analyses are adjusted for baseline measurement of each outcome. All analyses are conducted with adjustment for baseline imbalances. Values are presented as n(n-n%), indicating the mean value and its 95 % confidence interval. * indicates that a log transformation was applied, and the reported result is therefore an exponentiated, back-transformed value. ** indicates a p value < 0.05. Time points only for participants receiving full treatment: Treatment cessation, mean = 4.5 months (95%CI 4.3-4.7) after baseline; Follow-up, mean = 10.5 months (95%CI 10.2-10.7) after baseline. GPTS: Green Paranoid Thought Scale. GPTS: Green Paranoid Thought Scale. PSP: Personal and Social Performance scale. SBQ: Safety Behavior Questionnaire. ERT: Emotion Recognition Task. SIAS: Social Interaction Anxiety Scale.

Supplementary table 19| Per-protocol sensitivity analyses: Between-group adjusted mean difference adjusted for baseline imbalances: Sensitivity analyses on the exploratory outcomes

	VR-CBTp mean (95%CI)	CBTp mean (95%CI)	Adjusted mean difference (standard error)	95%CI for adjusted mean difference; p value
SAPS Global				
Treatment cessation	6.3 (6.0-6.7%)	6.4 (6.1-6.8%)	0.11 (0.27)	(-0.43 to 0.64%); 0.70
Follow-up	5.6 (5.1-6.1%)	5.8 (5.2-6.3%)	0.18 (0.41)	(-0.62 to 0.99%); 0.65
SAPS Composite				
Treatment cessation	16.1 (14.9-17.4%)	17.4 (16.1-18.7%)	1.30 (0.94)	(-0.56 to 3.15%); 0.17
Follow-up	13.9 (12.4-15.4%)	14.8 (13.2-16.4%)	0.94 (1.13)	(-1.30 to 3.18%); 0.41
BNSS total				
Treatment cessation	19.9 (18.7-21.1%)	21.0 (19.8-22.3%)	1.12 (0.90)	(-0.66 to 2.89%); 0.22
Follow-up	19.4 (17.7-21.1%)	18.7 (16.8-20.6%)	-0.68 (1.33)	(-3.31 to 1.95%); 0.61
CDSS total				
Treatment cessation	4.5 (3.9-5.1%)	4.6 (4.0-5.3%)	0.72* (0.25)	(0.44 to 0.19%)*; 0.20
Follow-up	4.9 (4.1-5.6%)	4.8 (3.9-5.7%)	-0.08 (0.61)	(-1.28 to 1.12%); 0.90
COGDIS total				
Treatment cessation	15.8 (14.4-17.3%)	17.4 (15.9-18.9%)	1.60 (1.07)	(-0.51 to 3.72%); 0.14
Follow-up	16.2 (14.3-18.0%)	15.9 (13.8-18.0%)	-0.25 (1.47)	(-3.16 to 2.67%); 0.87
Trustworthiness task				
Treatment cessation	-0.15 (-0.26 to -0.05%)	-0.11 (-0.22 to 0.00%)	0.05 (0.08)	(-0.11 to 0.20%); 0.56
Follow-up	-0.10 (-0.22 to 0.01%)	-0.13 (-0.26 to 0.00%)	-0.03 (0.09)	(-0.21 to 0.15%); 0.76
SSPA (SCOPE variables)				
Treatment cessation	4.39 (4.31-4.48%)	4.30 (4.20-4.39%)	-0.10 (0.07)	(-0.23 to 0.03%); 0.15
Follow-up	4.44 (4.36-4.52%)	4.38 (4.28-4.48%)	-0.06 (0.06)	(-0.19 to 0.07%); 0.36
IBT total				
Treatment cessation	0.52 (0.50-0.54%)	0.54 (0.51-0.56%)	0.02 (0.02)	(-0.02 to 0.05%); 0.35
Follow-up	0.53 (0.51-0.56%)	0.53 (0.50-0.56%)	-0.004 (0.02)	(-0.04 to 0.04%); 0.84
IBT Automatic				
Treatment cessation	0.56 (0.52-0.60%)	0.60 (0.56-0.65%)	0.05 (0.03)	(-0.02 to 0.11%); 0.16

Follow-up	0.59 (0.54-0.64%)	0.57 (0.51-0.62%)	-0.02 (0.04)	(-0.09 to 0.05%); 0.58
IBT Control				
Treatment cessation	0.41 (0.37-0.46%)	0.39 (0.35-0.44%)	-0.02 (0.03)	(-0.09 to 0.05%); 0.58
Follow-up	0.40 (0.35-0.44%)	0.37 (0.32-0.43%)	-0.02 (0.04)	(-0.10 to 0.05%); 0.51
SIDAS				
Treatment cessation	6.4 (5.0-7.8%)	8.5 (7.0-9.9%)	1.62 (0.41)	(0.73 to 3.61%)*; 0.23
Follow-up	6.7 (5.1-8.3%)	8.8 (7.1-10.6%)	2.17 (1.21)	(-0.22 to 4.55%); 0.07
BCSS – negative self				
Treatment cessation	7.8 (7.1-8.4%)	8.1 (7.5-8.8%)	0.36 (0.49)	(-0.61 to 1.33%); 0.47
Follow-up	7.4 (6.6-8.2%)	7.8 (6.9-8.7%)	0.36 (0.63)	(-0.88 to 1.60%); 0.57
BCSS – negative others				
Treatment cessation	5.6 (4.8-6.3%)	6.3 (5.5-7.1%)	1.09* (0.25)	(0.66 to 1.80%)*; 0.73
Follow-up	6.3 (5.5-7.2%)	6.0 (5.1-7.0%)	-0.31 (0.66)	(-1.60 to 0.99%); 0.64
BCSS – positive self				
Treatment cessation	8.6 (7.9-9.4%)	9.0 (8.2-9.7%)	0.35 (0.55)	(-0.73 to 1.43%); 0.53
Follow-up	9.1 (8.2-10.0%)	9.8 (8.8-10.7%)	0.65 (0.66)	(-0.66 to 1.96%); 0.33
BCSS – positive others				
Treatment cessation	10.1 (9.4-10.9%)	10.0 (9.2-10.8%)	-0.14 (0.57)	(-1.26 to 0.98%); 0.80
Follow-up	10.5 (9.5-11.5%)	10.3 (9.3-11.4%)	-0.15 (0.74)	(-1.61 to 1.31%); 0.84
DACOBS - Jumping to conclusion				
Treatment cessation	21.9 (21.0-22.7%)	21.8 (20.9-22.7%)	-0.04 (0.66)	(-1.33 to 1.26%); 0.96
Follow-up	21.8 (20.9-22.8%)	21.8 (20.7 – 22.8%)	-0.08 (0.75)	(-1.56 to 1.40%); 0.92
DACOBS - Belief inflexibility				
Treatment cessation	20.6 (19.8-21.5%)	19.8 (18.9-20.6%)	-0.88 (0.61)	(-2.08 to 0.32%); 0.15
Follow-up	19.9 (19.0-20.7%)	19.8 (18.8-20.7%)	-0.08 (0.67)	(-1.40 to 1.23%); 0.90
DACOBS - Attention for threat				
Treatment cessation	27.0 (26.0-27.9%)	27.7 (26.7-28.7%)	0.71 (0.71)	(-0.68 to 2.10%); 0.32
Follow-up	26.1 (24.9-27.3%)	26.8 (25.5-28.2%)	0.74 (0.95)	(-1.14 to 2.62%); 0.44
DACOBS - External attribution				
Treatment cessation	19.7 (18.8-20.5%)	20.7 (19.8-21.5%)	0.98 (0.62)	(-0.25 to 2.20%); 0.12

Follow-up	19.6 (18.6-20.6%)	19.6 (18.6-20.6%)	-0.01 (0.75)	(-1.50 to 1.48%); 0.99
DACOBS - Social cognitive problems				
Treatment cessation	26.1 (25.1-27.1%)	26.3 (25.3-27.4%)	0.21 (0.75)	(-1.28 to 1.70%); 0.78
Follow-up	25.5 (24.3-26.8%)	26.2 (24.9-27.5%)	0.67 (0.93)	(-1.17 to 2.50%); 0.48
DACOBS - Subjective cognitive problems				
Treatment cessation	26.6 (25.6-27.7%)	26.7 (25.6-27.7%)	0.02 (0.78)	(-1.52 to 1.56%); 0.98
Follow-up	26.5 (25.3-27.7%)	26.6 (25.3-27.9%)	0.11 (0.91)	(-1.69 to 1.91%); 0.91
DACOBS - Safety behavior				
Treatment cessation	20.2 (19.1-21.3%)	20.2 (19.0-21.3%)	0.01 (0.83)	(-1.63 to 1.65%); 0.99
Follow-up	19.5 (18.2-20.7%)	18.5 (17.1-19.8%)	-0.99 (0.98)	(-2.92 to 0.93%); 0.31
SFS – Social engagement/withdrawal				
Treatment cessation	97.7 (96.3-99.0%)	96.8 (95.4-98.2%)	-0.84 (1.01)	(-2.84 to 1.15%); 0.41
Follow-up	97.4 (95.6-99.2%)	99.1 (97.2-101.1%)	1.73 (1.37)	(-0.98 to 4.44%); 0.21
SFS – Interpersonal behavior				
Treatment cessation	114.1 (111.6-116.5%)	112.4 (109.8-115.0%)	-1.65 (1.86)	(-5.33 to 2.02%); 0.38
Follow-up	114.2 (111.3-117.0%)	114.4 (111.4-117.4%)	0.24 (2.14)	(-3.98 to 4.46%); 0.91
SFS - Recreation				
Treatment cessation	107.5 (105.6-109.3%)	107.2 (105.2-109.2%)	-0.24 (1.42)	(-3.04 to 2.57%); 0.87
Follow-up	106.9 (104.4-109.4%)	109.7 (106.9-112.4%)	2.75 (1.92)	(-1.03 to 6.53%); 0.15
SFS – Independence-competence				
Treatment cessation	103.6 (102.0-105.2%)	103.0 (101.3-104.7%)	-0.56 (1.21)	(-2.95 to 1.82%); 0.64
Follow-up	104.2 (102.0-106.3%)	104.5 (102.2-106.7%)	0.30 (1.60)	(-2.85 to 3.45%); 0.85
SFS – Independence-performance				
Treatment cessation	100.2 (98.7-101.6%)	99.8 (98.2-101.3%)	-0.40 (1.12)	(-2.61 to 1.80%); 0.72
Follow-up	101.3 (99.4-103.2%)	101.6 (99.5-103.7%)	0.28 (1.46)	(-2.61 to 3.17%); 0.85
SFS – Employment-occupation				
Treatment cessation	104.4 (102.7-106.1%)	104.5 (102.8-106.3%)	0.12 (1.28)	(-2.41 to 2.65%); 0.93

Follow-up	105.6 (103.3-107.9%)	104.8 (102.4-107.3%)	-0.77 (1.71)	(-4.15 to 2.61%); 0.65
GSE				
Treatment cessation	24.3 (23.4-25.3%)	23.5 (22.5-24.4%)	-0.85 (0.70)	(-2.23 to 0.54%); 0.23
Follow-up	24.4 (23.3-25.5%)	24.8 (23.6-26.0%)	0.40 (0.84)	(-1.27 to 2.07%); 0.64
EQ-5D-5L				
Treatment cessation	0.63 (0.58-0.67%)	0.58 (0.53-0.62%)	-0.05 (0.03)	(-0.12 to 0.02%); 0.13
Follow-up	0.64 (0.60-0.69%)	0.65 (0.61-0.70%)	0.01 (0.03)	(-0.06 to 0.08%); 0.74
EQ5 VAS				
Treatment cessation	60.8 (56.7-64.8%)	56.6 (52.3-60.9%)	-4.15 (3.06)	(-10.19 to 1.90%); 0.18
Follow-up	61.4 (56.9-65.8%)	59.7 (54.9-64.5%)	-1.66 (3.37)	(-8.32 to 4.99%); 0.62
WHO 5				
Treatment cessation	40.8 (37.4-44.2%)	41.7 (38.1-45.2%)	0.84 (2.55)	(-4.20-5.87%); 0.74
Follow-up	42.9 (38.6-47.3%)	43.6 (39.1-48.2%)	0.70 (3.24)	(-5.69 to 7.10%); 0.83
GPTS-R – Ideas of Persecution				
Treatment cessation	8.1 (6.7-9.4%)	8.7 (7.4-10.1%)	0.92* (0.30)	(0.51 to 1.67%)*; 0.79
Follow-up	7.8 (6.2-9.4%)	8.7 (7.0-10.4%)	0.93 (1.21)	(-1.45 to 3.32%); 0.44
GPTS-R – Ideas of Social Self-Reference				
Treatment cessation	8.3 (7.4-9.3%)	8.6 (7.7-9.6%)	0.30 (0.70)	(-1.08 to 1.68%); 0.67
Follow-up	8.2 (7.0-9.3%)	8.8 (7.6-10.0%)	0.64 (0.89)	(-1.11 to 2.39%); 0.47
GPTS total				
Treatment cessation	61.2 (58.6-65.9%)	64.6 (60.8-68.4%)	2.38 (2.75)	(-3.04 to 7.80%); 0.39
Follow-up	61.1 (56.3-65.9%)	64.3 (59.1-69.4%)	3.19 (3.65)	(-4.01 to 10.39%); 0.38
GPTS-R total				
Treatment cessation	16.5 (14.6-18.5%)	17.3 (15.3-19.4%)	0.78 (1.47)	(-2.12 to 3.69%); 0.60
Follow-up	15.9 (13.4-18.5%)	17.4 (14.7-20.2%)	1.48 (1.94)	(-2.36 to 5.31%); 0.45
CSQ				
Treatment cessation	27.2 (26.5-28.0%)	26.2 (25.5-27.0%)	-0.99 (0.56)	(-2.10 to 0.12%); 0.08

Only participants receiving full treatment (10 sessions) are analysed (VR-CBTp: n=102, CBTp: n=97). Between-group adjusted mean difference after adjusting for biological sex assigned at birth, study site and dichotomised symptom severity of GPTS subscale *Ideas of Persecution* (≥45 or <45 at baseline). All analyses are conducted with adjustment for baseline imbalances. All analyses are linear regression models handled with multiple imputations. All analyses are adjusted for baseline measurement of each outcome, except for the CSQ that was not administered at baseline. For CSQ, a linear regression model adjusted for biological sex assigned at birth, study site and dichotomised symptom

severity of GPTS subscale *Ideas of Persecution* (≥45 or <45 at baseline) was used. Values are presented as n(n-n%), indicating the mean value and its 95 % confidence interval. * indicates that a log transformation was applied, and the reported result is therefore an exponentiated, back-transformed value. Time points only for participants receiving full treatment: Treatment cessation, mean = 4.5 months (95%CI 4.3-4.7) after baseline; Follow-up, mean = 10.5 months (95%CI 10.2-10.7) after baseline. SAPS: Scale for the Assessment of Positive Symptoms. BNSS: Brief Negative Symptoms Scale. CDSS: Calgary Depression Symptom Scale. COGDIS: Cognitive Disturbances scale. SSPA: Social Skills Performance Assessment. IBT: Intentionality Bias Task. SIDAS: Suicidal Ideation Attributes Scale. BCSS: Brief Core Schema Scale. DACOBS: Davos Assessment of Cognitive Biases Scale. SFS: Social Functioning Scale. GSE: General Self-Efficacy scale. EQ-5D-5L: 5-Level EuroQol 5 Dimensions version. EQ5-VAS: EuroQol Visual Analogue Scale. WHO 5: World Health Organization-Five well-being index. GPTS-R: Revised Green Paranoid Thought Scale. CSQ: Client Satisfaction Questionnaire.

Supplementary table 20| Average amount and quality of exposure in each group during a treatment course

Exposure quantity			
	VR-CBTp	CBTp	p value
Exposure in minutes	84.5 (50.75-120), 83.5 (74.7-92.3%)	0 (0-0), 8.0 (4.3-11.6%)	P<0.001*

Exposure quality			
	VR-CBTp	CBTp	p value
Quality from 0-10	7.6 (6.5-8.4), 7.3 (7.0-7.6%)	7 (4.9-8.0), 6.4 (5.6-7.2%)	P=0.035*

Mann-Whitney U test was used to compare differences between groups for non-normally distributed numerical variables. Missing data were handled by imputing 0. Missing data were 16.3 % in the VR-CBTp group and 23.7 % in the CBTp group. Values are presented as n(n), indicating the median and its 25th and 75th percentile, (n(n%) indicates the mean and the corresponding 95%CI and * indicates p value < 0.05.
