# Supplementary material for: Virtual reality-based versus standard cognitive behavioral therapy for paranoia in schizophrenia spectrum disorders: a randomized controlled trial
Source: Nat Med. 2025 Aug 13;31(10):3425–39. doi: 10.1038/s41591-025-03880-8 (PMC12532561; doi:10.1038/s41591-025-03880-8)
Supplement: Supplementary file 2 — Reporting Summary [file 41591_2025_3880_MOESM2_ESM.pdf]

nature portfolio

Corresponding author(s): Louise Birkedal Glenthøj

Last updated by author(s): Jun 4, 2025

Reporting Summary

Nature Portfolio wishes to improve the reproducibility of the work that we publish. This form provides structure for consistency and transparency in reporting. For further information on Nature Portfolio policies, see our Editorial Policies and the Editorial Policy Checklist.

Statistics

For all statistical analyses, confirm that the following items are present in the figure legend, table legend, main text, or Methods section.

n/a	Confirmed	
☐	☒	The exact sample size (*n*) for each experimental group/condition, given as a discrete number and unit of measurement
☐	☒	A statement on whether measurements were taken from distinct samples or whether the same sample was measured repeatedly
☐	☒	The statistical test(s) used AND whether they are one- or two-sided<br>*Only common tests should be described solely by name; describe more complex techniques in the Methods section.*
☐	☒	A description of all covariates tested
☐	☒	A description of any assumptions or corrections, such as tests of normality and adjustment for multiple comparisons
☐	☒	A full description of the statistical parameters including central tendency (e.g. means) or other basic estimates (e.g. regression coefficient) AND variation (e.g. standard deviation) or associated estimates of uncertainty (e.g. confidence intervals)
☐	☒	For null hypothesis testing, the test statistic (e.g. *F*, *t*, *r*) with confidence intervals, effect sizes, degrees of freedom and *P* value noted<br>*Give P values as exact values whenever suitable.*
☐	☒	For Bayesian analysis, information on the choice of priors and Markov chain Monte Carlo settings
☒	☐	For hierarchical and complex designs, identification of the appropriate level for tests and full reporting of outcomes
☐	☒	Estimates of effect sizes (e.g. Cohen's *d*, Pearson's *r*), indicating how they were calculated

Our web collection on statistics for biologists contains articles on many of the points above.

Software and code

Policy information about availability of computer code

Data collection	For data entry and storage, an online data collection system for clinical trials (REDCap) was used. REDCap is an electronic data capture tool hosted at CIMT in the Capital Region of Denmark. REDCap has a complete audit trail on all data transactions, detailed user rights and access control management and thereby complies with the General Data Protection Regulation (Databeskyttelsesforordningen).
Data analysis	Research data was exported from REDCap without personal identifiers. Data was exported to well-known software packages: (SPSS and Stata) and put in logged folders on a network drive under the control of the Capital Region of Denmark, CIMT. A data manager ensured that all variables were properly defined with variable and value labels. All derived variables were properly defined, and algorithms were kept in special files. All data was examined carefully to identify errors in data entry. Data analysis comprising linear regression model and linear mixed model was carried out using Stata (Version 18.5). Descriptive analyses were carried out using Stata (Version 18.5) or IBM SPSS Statistics (Version 29.0.1.0). The forest plot was created using R version 4.5.0. The code is available at https://codeberg.org/VIRTU/faceyourfears.

For manuscripts utilizing custom algorithms or software that are central to the research but not yet described in published literature, software must be made available to editors and reviewers. We strongly encourage code deposition in a community repository (e.g. GitHub). See the Nature Portfolio guidelines for submitting code & software for further information.

Data

Policy information about availability of data

All manuscripts must include a data availability statement. This statement should provide the following information, where applicable:

- Accession codes, unique identifiers, or web links for publicly available datasets
- A description of any restrictions on data availability
- For clinical datasets or third party data, please ensure that the statement adheres to our policy

Open access information on the FaceYourFears trial is published on ClinicalTrials.gov (identifier: NCT04902066). The final protocol and subsequent update are published in Trials. All de-identified trial data are available through the Danish National Archives (Rigsarkivet), a public data repository, for an unlimited period. Access requests can be submitted via: https://www.rigsarkivet.dk.
Due to the Danish Archives Act (Arkivloven), the Danish Archives Executive Order (Arkivbekendtgørelsen), the General Data Protection Regulation (Databeskyttelsesforordningen), and the Danish Data Protection Act (Databeskyttelsesloven), access is restricted. For the first 20 years, data access is subject to prior review by the research group. Access must always be approved by the Danish Data Protection Agency (Datatilsynet), as the data are considered sensitive personal information. After 75 years, the data will be openly accessible without the need for approval.
In principle, the research group will grant access to academic or clinical researchers conducting non-commercial, ethically approved research. An initial response to access requests will be provided within one month. A Data Access Agreement must be signed prior to data sharing.

Research involving human participants, their data, or biological material

Policy information about studies with human participants or human data. See also policy information about sex, gender (identity/presentation), and sexual orientation and race, ethnicity and racism.

Reporting on sex and gender	Sex and gender were recorded solely in binary biological terms as sex assigned at birth, which limits our ability to explore potential gender differences in treatment outcomes. More attention should have been dedicated to this aspect as it limited our ability to explore potential gender differences in treatment outcomes.<br>More women than men participated in the study (CBTp (control): N=74 (57.8%, VR-CBTp (experimental): 72 (57.1%)).
Reporting on race, ethnicity, or other socially relevant groupings	Participants were classified by self-report. Socially relevant categorization variables were educational background, participants occupational status and type of outpatient routine care setting where participant received treatment.<br>The study lacks data on ethnicity or migrant status, preventing conclusions about the intervention's effectiveness for minority populations, even though existing literature shows that CBTp outcomes vary by ethnicity. Unfortunately, we were unable to include patients who spoke adequate English if they did not read adequate Danish, due to a lack of resources for translating treatment manuals. This has likely limited the participation of minority populations.
Population characteristics	All participants are patients in outpatient care facilities specialized in psychotic disorders in the Danish Public Healthcare system. All participants have a diagnosis of a schizophrenia spectrum disorder according to the ICD-10 classification (F20-29) and the most common diagnosis in both arms was schizophrenia (CBTp: N = 90 (70.3%), VR-CBTp: N= 94 (74.6%)). Median age was 26.5 years in the CBTp group and 27.7 years in the VR-CBTp group. The majority of participants in both groups were prescribed antipsychotic medication (CBTp: N = 102 (80.0%), VR-CBTp: N = 106 (84.0%)).
Recruitment	Patients were referred to the trial by their outpatient care settings. Participants were recruited in several ways: either by mutual agreement between the clinician and the potential participant for whom the project might would be of interest; through requests from participants or their relatives who had heard about the project in the medias or through word-of-mouth; or by assessors offering to review candidates who clinicians were uncertain whether they fulfilled eligibility criteria. Participants were included regardless of antipsychotic medication use, and medication changes did not lead to exclusion as well as a current history of alcohol or other psycho-active substance use were eligible. This has limited the extent of selection bias and our sample therefore reflects a broad group of patients in Danish outpatient care settings seeking therapeutic treatment. However, self-selection bias is likely to have occurred due to the following circumstances: 1) VR equipment is still not suitable for transportation and patients unable to leave their homes could therefore not participate. Thus, patients with such extreme avoidance behaviour due to paranoid anxiety had difficulties in participating; 2) The project was primarily introduced as an investigation of virtual reality-based therapy. This may have generated particular interest among young people and technology enthusiasts and older patients may, on this basis, have chosen not to participate.
Ethics oversight	The study was approved by the Committee on Health Research Ethics of the Capital Region of Denmark and the Danish Data Protection Agency. Project ID: H-20048806.

Note that full information on the approval of the study protocol must also be provided in the manuscript.

Field-specific reporting

Please select the one below that is the best fit for your research. If you are not sure, read the appropriate sections before making your selection.

☒ Life sciences ☐ Behavioural & social sciences ☐ Ecological, evolutionary & environmental sciences

For a reference copy of the document with all sections, see nature.com/documents/nr-reporting-summary-flat.pdf

Life sciences study design

All studies must disclose on these points even when the disclosure is negative.

Sample size	The sample size calculation was based on the primary outcome, the GPTS subscale ideas of persecution, and the between-group difference at treatment cessation. A clinically significant group difference was defined as Cohen's d ≥ 0.33, which corresponds to a difference of 6.0 or more on the GPTS subscale Ideas of Persecution. We utilized a pooled standard deviation (SD) of 17.1, obtained from a previous study. To achieve 80% power with a two-sided alpha level of 0.05, the trial required a total of 256 participants, with 128 participants randomly assigned to each of the two intervention arms.
Data exclusions	Five participants withdrew their informed consent after being included, and all data we had collected on these five participants was deleted, resulting in the exclusion of their data.
Replication	All treatment sessions were audio recorded to assess treatment fidelity. An independent experienced clinical psychologist evaluated fidelity to the treatment manuals by rating seven randomly selected treatment courses from each intervention using the Cognitive Therapy Rating Scale. This scale comprises 11 items, each scored on a range from 0 to 6. The mean score for all 11 items was calculated for each session. For each of the two sets of seven treatment courses, one session was randomly selected from the beginning, middle, and end of the course. I.e., in total, two sets of 21 sessions were evaluated. In the VR-CBTp group, therapists demonstrated "good" to "very good" fidelity to the treatment manual. In CBTp, therapists demonstrated "good" to "very good" fidelity to the treatment manual. We calculated inter-rater reliability for those primary and secondary outcomes, which consisted of interviews by using intra-class correlations. Intra-class correlations corresponded to good and excellent agreement. Unfortunately, we did not calculate Intra-class correlation for explorative outcomes. All therapist in the North Denmark Region were employed in outpatient care settings and participated in the study on a part-time basis. Most therapists received a 2-day course in both manualized treatments, while two therapists became involved late in the study and received side-by-side training. Ongoing internal consultation was provided weekly during the first year of the trial and bi-weekly thereafter in the Capital Region of Denmark. In North Denmark Region, internal consultation was not scheduled until one year after the initial treatment course had begun. Monthly external online supervision by an international expert in both modalities was conducted during the trial's first 18 months. The therapist who delivered most treatment courses had limited experience with CBT (one year) and psychosis-therapy interventions (one year). This indicates that the interventions evaluated in our study can be feasibly implemented in current clinical settings, by less experienced therapists, if appropriate training and supervision is provided. The intervention is complex and intended for the treatment of severe psychopathology. Proper use of the manual requires formal training and supervised practice to ensure patient safety and treatment fidelity. Please contact the corresponding author, LBG (Louise.Birkedal.Glenthoej@regionh.dk), for access to, and guidance on the use of the treatment manuals.
Randomization	Participants were randomly assigned (1:1) to VR-CBTp plus TAU or CBTp plus TAU with a variable block size created by an independent statistician and kept concealed from all study personnel, including assessors. Randomization was conducted following the baseline assessment using a centralised, computer-generated system. Non-masked personnel informed participants of their assigned allocation.
Blinding	Therapists, participants, and clinicians in outpatint care settings were aware of treatment allocation, but assessment of outcome was conducted by research assistants masked to treatment allocation. Masking of assessors was preserved by separating assessors from therapists and participants was instructed not to disclose their allocation. If unmasking occurred, patients were reassigned to a different masked assessor. Video recordings of interviews allowed masked assessment to be conducted later if unmasking occurred. If unmasking occurred during a non-recorded interview, the interview was discontinued, and reassessment was scheduled with another masked assessor. Unmasking occurred altogether five times and re-masking were successful in all cases. The initial dropout analysis was performed by UNJ and CH. CH conducted the linear regression model, and linear mixed model analyses. Both UNJ and CH were masked to group in the data analysis phase. The conclusion on treatment efficacy was written and agreed upon amongst UNJ, DLV, ASD, LM, AP, SA, MJC, NKH, LCS, CH, WV, MN and LBG while remaining blinded to group allocation.

Reporting for specific materials, systems and methods

We require information from authors about some types of materials, experimental systems and methods used in many studies. Here, indicate whether each material, system or method listed is relevant to your study. If you are not sure if a list item applies to your research, read the appropriate section before selecting a response.

Materials & experimental systems

n/a	Involved in the study	
☒	☐	Antibodies
☒	☐	Eukaryotic cell lines
☒	☐	Palaeontology and archaeology
☒	☐	Animals and other organisms
☐	☒	Clinical data
☒	☐	Dual use research of concern
☒	☐	Plants

Methods

n/a	Involved in the study	
☒	☐	ChIP-seq
☒	☐	Flow cytometry
☒	☐	MRI-based neuroimaging

Clinical data

Policy information about clinical studies

All manuscripts should comply with the ICMJE guidelines for publication of clinical research and a completed CONSORT checklist must be included with all submissions.

Clinical trial registration	NCT04902066
Study protocol	DOI: 10.1186/s13063-022-06614-0. Protocol update: DOI: 10.1186/s13063-023-07069-7

Data collection

Participants underwent assessment procedures in two regions of Denmark (i.e., Capital Region and North Denmark Region). The first participant was enrolled on April 9th, 2021. The final follow-up assessment was conducted on August 10th, 2024, marking the conclusion of the data collection phase.

When necessary, data collection was done in paper and later entered electronically. Data on paper was stored locally and secured and only assigned researchers could access REDCap In REDCap, self-report questionnaire could, if needed, be digitally sent to E-boks and the completed form was returned digitally reducing risk of data loss and leak. E-boks is a public Danish mailing system all inhabitants are in possession of where person sensitive information can be sent safety.

Outcomes

Primary, secondary, and exploratory outcome measures were pre-specified and described in the protocol and on Clinicaltrials.org. Assessments were carried out at baseline, treatment cessation and follow-up (6 months after treatment cessation) by trained raters, all of which were either psychologists or medical doctors. Assessment included both structured as well as semi-structured interviewing and social cognitive measurements. In cases of self-report measures, participants functioned as assessors themselves with guidance from masked assessors if participants requested it. The first author of this article (UNJ) is a medical doctor. UNJ was responsible for the training of assessors with guidance from the last author, LBG, who is a psychologist with specialisation in psychiatry. In the training process supervised co-interviews were conducted. During the data collection phase, videotaped assessments were distributed among assessors for monthly consensus meetings the first two years and thereafter bi-monthly, where inter-rater reliability ratings on the secondary outcome measures Personal and Social Performance scale and Safety behavior questionnaire were performed or other measurements were discussed to reach consensus and to clarify psychopathology in case of doubts.

-The primary outcome was the GPTS subscale Ideas of Persecution (self-report questionnaire, score range 16-80), measured at treatment cessation. The GPTS subscale Ideas of Persecution has demonstrated good psychometric properties overall in large clinical and non-clinical samples but the presence of i.e. local dependence highlights that there is a potential for measurement errors.
-The secondary outcomes of paranoia were GPTS subscale Ideas of Persecution, measured at follow-up, and GPTS subscale Ideas of Social Self-Reference, measured at treatment cessation and follow-up.
Other secondary outcomes, all measured at treatment cessation and follow-up:
-The Safety behaviour Questionnaire (SBQ) (semi-structured interview). In the original questionnaire, closed-ended questions are used to uncover common situations that patients with persecutory delusions tend to avoid. Avoidance was the most frequent safety behaviour (92%) observed. We decided to include, besides the original open-ended question, supporting closed-ended questions for the second most frequent safety behaviour, In-situation (68 %), as people with lived experiences, fulfilling eligibility criteria, who provided us with feedback, gave us the impression that these behaviours would often be present in our sample but rarely recognised by patients themselves. Consequently, we selected a list consisting of the most common in-situation behaviours mentioned in the original study, which covered four themes: protection, invisibility, vigilance, and resistance. We further decided to calculate a sub-score of avoidance as this safety behaviour is considered especially challenging.
-The Personal and Social Performance Scale (PSP) (semi-structured interview).
-The Social Interaction Anxiety Scale (SIAS) (self-report questionnaire).
-The CANTAB Emotion Recognition Task (ERT) long Caucasian version (social-cognitive test). We decided to calculate latency and accuracy both as total scores and sub-scores for each emotion (happiness, sadness, fear, anger, surprise, and disgust) as distinct emotions have shown to be differently impaired in SSD.
Exploratory outcomes, measured at treatment cessation and follow-up:
-The Scale for the Assessment of Positive Symptoms (SAPS) (semi-structured interview). Items were assessed based on the recent month.
-The Brief Negative Symptoms Scale (BNSS) (semi-structured interview).
-The Cognitive Disturbances scale (COGDIS) (semi-structured interview). COGDIS was included to capture subtle, non-psychotic anomalous experiences, particularly among participants with schizotypal disorder (F21 diagnosis). While traditionally associated with clinical high-risk (CHR) groups, the inclusion of COGDIS aimed to provide a more nuanced assessment of symptomatology beyond what was captured by SAPS. One of the COGDIS items, “tendencies of unstable self-reference,” was in our study by default given the highest score of six if participants described paranoia as paranoid ideation (ideas of self-reference or persecution) or delusion on daily basis. We decided this as less disturbing subtle experiences tend to recede and become obscured when more severe experiences within the same domain emerge.
-The Calgary Depression Symptom Scale (CDSS) (sstructured interview).
-The Brief Core Schema Scale: belief about self and others (BCSS) (self-report questionnaire).
-The General Self-Efficacy scale (GSE) (self-report questionnaire).
-The Davos Assessment of Cognitive Biases Scale (DACOBS) (self-report questionnaire).
-The Suicidal Ideation Attributes Scale (SIDAS) (self-report questionnaire).
-The Intentionality Bias Task (IBT) (social-cognitive test). Paradigm was built in E-prime. We used the 24-item version from the SCOPE study and calculated total, automatic and control.
-The Trustworthiness task (social-cognitive test).
-The Social Functioning Scale (SFS) (self-report questionnaire). The SFS prosocial activities subscale was excluded due to its sensitivity to COVID-19 restrictions in Denmark (March 2020-February 2022).
-The Social Skills Performance Assessment (SSPA) (semi-structured roleplay). We used the SCOPE study version.
-The WHO-5 Well Being Index (self-report questionnaire).
-The EuroQOL five dimensions questionnaire (EQ-5D-5L) (self-report questionnaire).

A Big-5 personality traits 25 items 5-point Likert scale (self-report questionnaire) and the Trauma And Life Events checklist (TALE) (self-report questionnaire) were measured at baseline.
The Client Satisfaction Questionnaire (CSQ) (self-report questionnaire) was measured at treatment cessation.
The Revised GPTS (R-GPTS) (self-report questionnaire) was measured at treatment cessation and follow-up to conduct a sensitivity analysis on the GPTS.
